# The chromosome-level draft genome of *Dalbergia odorifera*

**DOI:** 10.1093/gigascience/giaa084

**Published:** 2020-08-18

**Authors:** Zhou Hong, Jiang Li, Xiaojin Liu, Jinmin Lian, Ningnan Zhang, Zengjiang Yang, Yongchao Niu, Zhiyi Cui, Daping Xu

**Affiliations:** State Key Laboratory of Tree Genetics and Breeding, Research Institute of Tropical Forestry, Chinese Academy of Forestry, Guangzhou 510520, China; Biozeron Shenzhen Inc., Shenzhen 518000, China; State Key Laboratory of Tree Genetics and Breeding, Research Institute of Tropical Forestry, Chinese Academy of Forestry, Guangzhou 510520, China; Biozeron Shenzhen Inc., Shenzhen 518000, China; State Key Laboratory of Tree Genetics and Breeding, Research Institute of Tropical Forestry, Chinese Academy of Forestry, Guangzhou 510520, China; State Key Laboratory of Tree Genetics and Breeding, Research Institute of Tropical Forestry, Chinese Academy of Forestry, Guangzhou 510520, China; Biozeron Shenzhen Inc., Shenzhen 518000, China; State Key Laboratory of Tree Genetics and Breeding, Research Institute of Tropical Forestry, Chinese Academy of Forestry, Guangzhou 510520, China; State Key Laboratory of Tree Genetics and Breeding, Research Institute of Tropical Forestry, Chinese Academy of Forestry, Guangzhou 510520, China

**Keywords:** *Dalbergia odorifera* T. Chen, *de novo* sequencing, chromosome-level genome assembly, annotation, phylogeny

## Abstract

**Background:**

*Dalbergia odorifera* T. Chen (Fabaceae) is an International Union for Conservation of Nature red-listed tree. This tree is of high medicinal and commercial value owing to its officinal, insect-proof, durable heartwood. However, there is a lack of genome reference, which has hindered development of studies on the heartwood formation.

**Findings:**

We presented the first chromosome-scale genome assembly of *D. odorifera* obtained on the basis of Illumina paired-end sequencing, Pacific Biosciences single-molecule real-time sequencing, 10x Genomics linked reads, and Hi-C technology. We assembled 97.68% of the 653.45 Mb *D. odorifer*a genome with scaffold N50 and contig sizes of 56.16 and 5.92 Mb, respectively. Ten super-scaffolds corresponding to the 10 chromosomes were assembled, with the longest scaffold reaching 79.61 Mb. Repetitive elements account for 54.17% of the genome, and 30,310 protein-coding genes were predicted from the genome, of which ~92.6% were functionally annotated. The phylogenetic tree showed that *D. odorifera* diverged from the ancestor of *Arabidopsis thaliana* and *Populus trichocarpa* and then separated from *Glycine max* and *Cajanus cajan*.

**Conclusions:**

We sequence and reveal the first chromosome-level *de novo* genome of *D. odorifera*. These studies provide valuable genomic resources for the research of heartwood formation in *D. odorifera* and other timber trees. The high-quality assembled genome can also be used as reference for comparative genomics analysis and future population genetic studies of *D. odorifera*.

## Background

*Dalbergia odorifera* T. Chen (NCBI:txid499988), formerly named *Dalbergia hainanensis* Merr. et Chun, is a medium-sized evergreen tree belonging to the Fabaceae family. *D. odorifera* originated in Hainan, China, and has been gradually introduced and cultivated in Guangdong, Fujian, Zhejiang, Guangxi, and Yunnan, China. *D. odorifera* is an ideal biological model to study the mechanism underlying high-quality heartwood (HW) formation owing to its insect-proof, durable, fragrant, beautiful HW [1]. HW is defined as the central wood layers of a tree (Supplementary Fig. S1). This tissue, containing nonliving cells and nonfunctioning xylem tissue, can affect tree health, with broader implications for forest health [2]. The natural durability of wood as well as the biological, technological, and aesthetic parameters of wood and wood products depend on the presence, quality, and quantity of HW, which is strongly affected by external stimuli [3]. Flavonoids, which are the major compounds found in *D. odorifera*, are a main class of secondary metabolites that strongly affect various properties of HW, including durability and the color of wood products [2]. Besides, flavonoids are crucial for plant resistance against pathogenic bacteria and fungi, and flavonoid production can be induced by fungal invasion [4]. It is worth noting that carbohydrates can also affect flavonoid accumulation and the formation of phenolic extractives, which contribute to the natural durability of wood during HW formation [5]. Apart from its excellence as a wood product, the HW of *D. odorifera*, which is known as “JiangXiang” in traditional Chinese medicine, has been included in the Chinese Pharmacopoeia for decades and is widely used to dissipate stasis, stop bleeding, and relieve pain. *D. odorifera* HW is also used to treat blood stagnation syndrome, ischemia, swelling, necrosis, and rheumatic pain in Korea [6]. Owing to its great medicinal and commercial value, *D. odorifera* is becoming more and more rare: only limited numbers of individuals are found in parts of their original habitat, with highly fragmented populations present in the remaining forests of Hainan Island [7]. It is worth noting that *D. odorifera* has been listed on the International Union for Conservation of Nature red list by the World Conservation Monitoring Centre since 1998 [8].

Despite the commercial interest and increasing demand for *D. odorifera*, the lack of a genome sequence for this species has limited analysis of the mechanism underlying HW formation in *D. odorifera*, which has seriously hampered conservation and breeding efforts. Advances in sequencing and assembly technology have made it possible to obtain chromosome-level reference genome sequences for organisms once thought to be intractable, including forest trees, which always have high heterozygosity.

Herein, we used Illumina short reads, Pacific Biosciences (PacBio) single-molecule real-time (SMRT) sequencing long reads, Hi-C data, and 10x Genomics linked-reads data to assemble the first chromosome-level genome of *D. odorifera*. We revealed the genomic features of *D. odorifera*, including repeat sequences, gene annotation, and evolution. This high-quality genome could provide the fundamental genetic information to study the durable HW formation of *D. odorifera* and related species.

## Data Description

### Sample collection and sequencing

An individual plant of cultivar *D. odorifera* from Tianhe district (113 39.134 E,23 19.705 N), Guangzhou, China, was chosen as a tissue source for sequencing. After collection healthy, fresh leaves were snap-frozen in liquid nitrogen, followed by preservation at −80°C in the laboratory prior to DNA extraction. To obtain the whole-genome sequences, genomic DNA was extracted using the cetyltrimethylammonium bromide (CTAB) method [9]. The quality and quantity of the isolated DNA were checked by electrophoresis on a 1% agarose gel and a NanoPhotometer® spectrophotometer (IMPLEN, CA, USA), and the DNA was then accurately quantified using Flurometer (Life Technologies, CA, USA).

To generate a chromosome-scale assembly, 4 different technologies were applied: Illumina paired-end (PE) sequencing, PacBio SMRT sequencing, 10x Genomics link-reads, and Hi-C technology. NEB Next® Ultra DNA Library Prep Kit (USA) was used to construct the Illumina PE library. A 0.5-μg aliquot of genomic DNA molecules was fragmented, end-paired, and ligated to adaptor. The ligated fragments were fractionated on agarose gels and purified by PCR amplification to produce sequencing libraries. Two PE libraries were constructed with insert length of 350 bp according to the manufacturer's protocol (Illumina, San Diego, CA, USA) and 73.88 Gb (113.06× coverage) sequencing data were produced on the Illumina HiSeq 4000 System (Illumina HiSeq 3000/HiSeq 4000 System, RRID:SCR_016386). The preparation of the PacBio library followed the protocol “Preparing *Arabidopsis* Genomic DNA for Size-Selected ~20 kb SMRTbell™ Libraries” [10]. PacBio reads were sequenced by the Sequel platform (PacBio Sequel System, RRID:SCR_017989), which gained 67.80 Gb (103.76× coverage) sequencing data. Subreads were filtered with the default parameters. Finally, we obtained 67.74 Gb of subreads dat. The mean and N50 length of long subreads reached 11.20 and 17.40 kb, respectively (Supplementary Table S2). For 10x Genomics library preparation, purified high molecular weight genomic DNA of high quality was incubated with Proteinase K and RNaseA for 30 minutes at 25°C. DNA was further purified, indexed, and partitioned into bar-coded libraries that were prepared using the GemCode kit (10x Genomics, Pleasanton, CA). Following the GemCode procedure, 1.0 ng of DNA was used for gel beads in emulsion (GEM) reactions in which DNA fragments were partitioned into molecular reactors to extend the DNA and to introduce specific 14-bp partition bar codes. Subsequently, GEM reactions were PCR amplified. The PCR cycling protocol was as follows: 95°C for 5 minutes; cycled 18×: 4°C for 30 seconds, 45°C for 1 second, 70°C for 20 seconds, and 98°C for 30 seconds; held at 4°C. The PCR products were purified as described in the GemCode protocol. Purified DNA was sheared, end-repaired, adenylation tailed, and universal adapter ligated, and samples were indexed according to the manufacturer's recommendations. The whole-genome GemCode library with an insert size of 500 bp was sequenced using 150 bp PE mode sequencing on Illumina HiSeq 4000 System, which produced 118.27 Gb (180.99× coverage) sequencing data. The whole-genome Hi-C library was constructed as described in the manufacturer's recommendations [11] and sequenced with 150 bp PE sequencing on Illumina Hiseq 4000 System, of which 156.84 Gb (240.02× coverage) data were generated. The enzyme used in the Hi-C library was DpnII, and it used cuts at “GATC.” All the raw sequence data generated by the Illumina platform were filtered by the following criteria: filtered reads with adapters, filtered reads with N bases >10%, and filtered reads with low-quality bases (≤5) >50%. All sequence data are summarized in Table 1.

**Table 1:** Sequencing data used for the *D. odorifera* genome assembly

Libraries	Insert size (bp)	Raw data (Gb)	Clean data (Gb)	Read length (bp)	Sequence coverage (×)
Illumina reads	350	73.88	73.80	150	113.06
PacBio reads	20,000	67.80	67.74	11,201	103.76
10x Genomics	500	118.27	116.06	150	180.99
Hi-C	350	156.84	155.86	150	240.02
Total		416.79	413.46		637.83

The coverage was calculated using an estimated genome size of 653.45 Mb.

To fully assist genome annotation, 5 tissues (flower, leaf, root, seed, and stem) were collected. Total RNA from each tissue was isolated using the NEBNext Poly (A) messenger RNA (mRNA) Magnetic Isolation Module and treated with DNaseI (Thermo Fisher Scientific, Wilmington, DE, USA) to remove genomic DNA. The RNA integrity of each tissue was confirmed with a Bioanalyzer 2100 (Agilent Technologies, Santa Clara, CA, USA). For each tissue, an RNA-sequencing (RNA-seq) library was prepared using the NEBNext Ultra RNA Library Prep Kit for Illumina with an insert size of 300 bp. An Illumina HiSeq 4000 System was used to perform 150 -bp PE mode sequencing. After removing reads containing adapters, reads containing poly-N, and low-quality reads from the raw data, 44.85 Gb of clean data were generated (Supplementary Table S11).

### Genome size estimation and genome assembly

The genome size of *D. odorifera* was estimated with the following formula: genome size = [Num (total *k*-mer) − Num (erroneous *k*-mer)]/mean depth of *k*-mer. 17-mers were counted for a total of 55,992,417,644 from short clean reads. The total count of error *k*-mers was 449,157,644, and the *k*-mer depth was 85 (Supplementary Table S3; Supplementary Fig. S2). The genome size of *D. odorifera* was estimated to be ~653.45 Mb, and the heterozygosity was ~0.54%. Besides, we used ~25× Illumina PE sequencing clean reads to perform a GenomeScope (GenomeScope, RRID:SCR_017014) v1.0.0 [12] analysis. The rate of genome heterozygosity estimated by GenomeScope was ~0.59% and the genome size was ~623.46 Mb (Supplementary Fig. S3).

**Figure 1:**
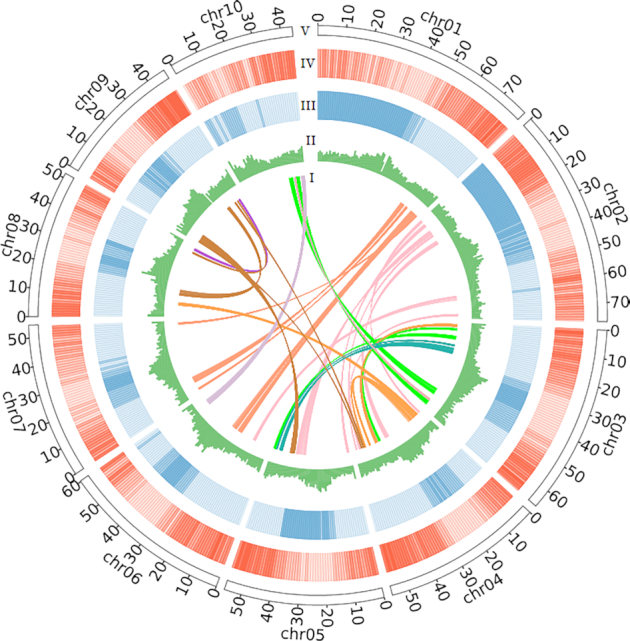
Circos plot shows the characterization of the *D.odorifera* genome. I: Syntenic regions within *D. odorifera* assembly based on homology searches were found with MCscan [51] requiring ≥30 genes per block (links); II: GC content in non-overlapping 1-Mb windows (histograms); III: Percent coverage of TEs in non-overlapping 1-Mb windows (heat maps); IV: Gene density calculated on the basis of the number of genes in non-overlapping 1 Mb windows (heat maps); V: Length of super-scaffolds in megabase pairs.

Falcon (Falcon, RRID:SCR_016089) v2.0.5 [13] assembler was used to assemble the contig of the *D. odorifera* genome, with parameters as follows: –max_diff 100 –max_cov 100 –min_cov 2 –min_len 5000. After Falcon assembly, the genome was polished by Quiver v5.0 [14]. Illumina short reads were used to produce a more contiguous genome with fewer errors with Pilon (Pilon, RRID:SCR_014731) v1.18 [15]. Initial assembly of the PacBio data alone resulted in a contig N50 of 6.19 Mb. The PacBio contigs were first scaffolded on the basis of 10x Genomics linked-read data using fragScaff v140324 [16] software, and the resulting scaffolds were further connected to super-scaffolds by Hi-C technology using the methods described by Bickhart et al. [17]. According to the Hi-C clustering results, the genome sequences were divided into 10 chromosome clusters (Fig. 1; Supplementary Table S7; Supplementary Fig. S6). Each technology greatly improved the assembly quality (Supplementary Table S9). All these processes yielded a final draft *D. odorifera* genome assembly with a total length of 638.26 Mb, scaffold N50 of 56.16 Mb, and longest scaffold of 79.61 Mb (Table 2). The N50 of this assembly is amongst the best quality Fabaceae family genomes that have been completed to date (Supplementary Fig. S7).

**Table 2:** Statistics for the *D. odorifera* genome

Assembly feature	Value
Estimated genome size (by *k*-mer analysis)	653.45 Mb
No. of scaffolds	384
Contig N50	5.92 Mb
Scaffold N50	56.16 Mb
Longest scaffold	79.61 Mb
Assembly length	638.26 Mb
Assembly % of genome	97.68
Repeat region % of assembly	54.17
Predicted gene models	30,310
Mean coding sequence length	1121.36 bp
Mean exons per gene	4.93

To assess the quality of the genome assembly, we mapped PE reads with short insert sizes onto the assembly using BWA (BWA, RRID:SCR_010910) v 0.7.17-r1188 [18] with the mem algorithm. Nearly 98% of these reads could be mapped to the *D. odorifera* draft genome, with the genome coverage reaching 99.63% (Supplementary Table S4). In addition, of the 1,440 genes identified using Embryophyta BUSCO (BUSCO, RRID:SCR_015008 v3.0 [19]), 92.2% complete and 1.7% partial genes were identified in the assembled genome (Supplementary Table S6), and 235 of the 248 genes identified using CEGMA (CEGMA v2.5 [20]) were retrieved in the assembly (Supplementary Table S5). These results indicate that the newly generated *D. odorifera* genome is of high quality and we successfully assembled the major genic regions of this precious plant species.

### Repeat sequences in the *D. odorifera* genome assembly

Homology searching and *ab initio* prediction were applied to study the repetitive sequences in the *D. odorifera* genome. For homology-based prediction, we used RepeatMasker (RepeatMasker, RRID:SCR_012954) v4.0.7 [21] and RepeatProteinMask v4.0.7 to search against Repbase [22]. For *ab initio* prediction, we used Tandem Repeats Finder v4.07b [23], LTR_FINDER (LTR_Finder, RRID:SCR_015247) v 1.07 [24], RepeatModeler (RepeatModeler, RRID:SCR_015027) v1.0.8, Piler v1.0 [25], and RepeatScout (RepeatScout, RRID:SCR_014653) v1.0.5 [26] with default parameters. Overall, the identified repeat sequences in the *D. odorifera* genome accounted for 54.17% and total length of those accounted for 345.73 Mb (Supplementary Table S10). Long terminal repeats (LTRs) were the most abundant (comprising 37.7% of the genome), followed by DNA transposons (9.16% of the genome; Table 3).

**Table 3:** Classifications of transposable elements predicted by each method

Type	Repbase + Denovo		TE proteins		Combined TEs	
	Length (bp)	% in genome	Length (bp)	% in genome	Length (bp)	% in genome
DNA	55,376,910	8.68	10,310,575	1.62	58,455,913	9.16
LINE	6,620,833	1.04	4,004,118	0.63	9,241,306	1.45
SINE	292,685	0.05	0	0	292,685	0.05
LTR	236,380,844	37.03	56,821,684	8.9	240,620,255	37.7
Other	0	0	0	0	0	0
Satellite	116,222	0.02	0	0	116,222	0.02
Simple repeat	1,238,189	0.19	0	0	1,238,189	0.19
Unknown	48,638,508	7.62	0	0	48,638,508	7.62
Total	332,705,455	52.13	71,048,270	11.13	340,525,618	53.35

Note: Repbase + Denovo: RepeatMasker results based on Repbase, RepeatModeler, RepeatScout, Piler, and LTR_FINDER; TE proteins: RepeatProteinMask results based on Repbase; Combined TEs: combined results of Denovo + Repbase and TE proteins. LINE: long interspersed nuclear element; SINE: short interspersed nuclear element; TE: transposable element.

### Protein-coding gene prediction and non-coding RNA prediction

Three approaches were used to predict the protein-coding genes in the *D. odorifera* genome, including homologous comparison, *ab initio* prediction, and RNA-seq–based annotation. For homologous comparison, the reference protein sequences from the Ensembl database and NCBI database for 7 species, including *Arabidopsis thaliana, Populus trichocarpa, Eucalyptus grandis, Medicago truncatula, Arachis duranensis, Malus domestica*, and *Glycine max (*Supplementary Table S1) were aligned against the *D. odorifera* genome using TBLASTN (TBLASTN, RRID:SCR_011822) v2.2.15 [27] search with E-value 1e−5 in the “-F F” option. All TBLAST hits were concatenated after filtration of low-quality records. The sequence of each candidate gene was further extended upstream and downstream by 1,000 bp to represent the entire gene region. Gene structures were predicted using GeneWise (GeneWise, RRID:SCR_015054) v2.4.1 [28]. Genes predicted in a homology-based manner were viewed as the “Homology-set.” RNA-seq data derived from 5 tissues (Supplementary Table S11) were assembled using Trinity (Trinity, RRID:SCR_013048) v2.0 [29]. The assembled sequences (Supplementary Table S12) were aligned against the *D. odorifera* genome using PASA (PASA, RRID:SCR_014656) v2.3.3 [30], which assembles effective alignments into gene structures [31]. Gene models created by PASA were viewed as the PASA-T-set (PASA Trinity set). At the same time, RNA-seq reads were aligned to the *D. odorifera* genome using TopHat (TopHat, RRID:SCR_013035) v2.0.9 [32] with default parameters; then the mapped reads were assembled into gene models with Cufflinks (Cufflinks, RRID:SCR_014597) v2.2.1 [33] (Cufflinks-set). We simultaneously used 5 tools for *ab initio* prediction, including Augustus (Augustus, RRID:SCR_008417) v3.2.3 [34], GeneID (GeneID, RRID:SCR_002473) v1.4.4 [35], GeneScan (GENSCAN, RRID:SCR_012902) v1.0 [36], GlimmerHMM (GlimmerHMM, RRID:SCR_002654) v3.52 [37], and SNAP (SNAP, RRID:SCR_007936) v2006-07-28 [38]. Notably, the parameters were computationally optimized by training a set of high-quality protein sequences derived from the PASA gene models. Finally, the non-redundant reference gene set was generated using EVidenceModeler (EVidenceModeler, RRID:SCR_014659) v1.1.1. Weights for each type of evidence were as follows: PASA-T-set > Homology-set > Cufflinks-set > Augustus > GeneID = SNAP = GlimmerHMM = GeneScan. The gene models were further updated by PASA to identify untranslated regions and to obtain information about alternative splicing variation. Using this strategy, we annotated 30,311 protein-coding genes, with a mean coding sequence length of 1.12 kb and a mean of 4.93 exons per gene (Supplementary Table S13). A total of 96% of the genes were supported by homology searches and/or the presence of expressed transcripts (Supplementary Fig. S9). The gene components were similar to those of related species (Supplementary Fig. S10).

Four types of noncoding RNAs were annotated using tRNAscan-SE (tRNAscan-SE, RRID:SCR_010835) v1.23 and the Rfam (Rfam, RRID:SCR_007891) database v9.1 [39]. We found 112 ribosomal RNA, 582 transfer RNA (tRNA), 747 microRNA, and 473 small nuclear RNA genes in the *D. odorifera* genome (Supplementary Table S15).

### Functional annotation of protein-coding genes

First, gene functions were assigned to the translated protein-coding genes using BLASTP (BLASTP, RRID:SCR_001010) v2.2.15 [40] based on their highest match to proteins in the Swiss-Prot (Swiss-Prot, RRID:SCR_002380) [41], KEGG (KEGG, RRID:SCR_012773) [42], and NR databases. The BLASTP e-value cutoff was set as 1e−5. Second, motifs and domains in the protein-coding genes were retrieved by performing InterproScan (InterproScan, RRID:SCR_005829) v4.7 [43] searches against 6 protein databases: ProDom, PRINTS, Pfam, SMART, PANTHER, and PROSITE. GO (GO, RRID:SCR_002811) [44] terms for each gene were detected from the corresponding InterPro entries. In total, 92.6% (28,069 genes) were successfully annotated for ≥1 function term (Supplementary Table S14).

### Phylogenetic analysis and species divergence time estimation

**Figure 2:**
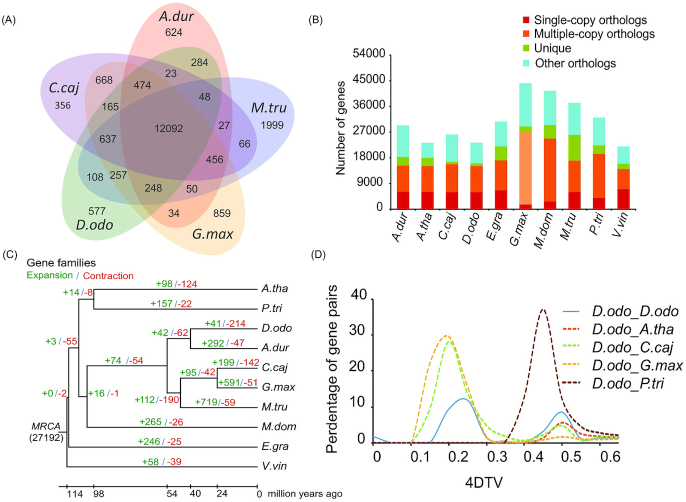
Evolution of the *D. odorifera* genome. (A) Venn diagram of shared and unique orthologous gene families in *D. odorifera* and 4 other legumes. (B) Predicted orthologous protein compositions for the 10 genomes. (C) Expansion and contraction of gene families. The numbers in green indicate the number of gene families that expanded in the species during evolution, and the numbers in red indicate the number of gene families that contracted. (D) 4DTV (4-fold degenerate transversion rate) plot. *A.tha*:*Arabidopsis thaliana; A. dur*:*Arachis duranensis; P.tri*:*Populus trichocarpa; D.odo*:*Dalbergia odorifera; C.caj*:*Cajanus cajan; G.max*:*Glycine max; M.tru*:*Medicago truncatula; M.dom*:*Malus domestica; E.gra*:*Eucalyptus grandis; V.vin*:*Vitis vinifera*.

To investigate the phylogenic positions and to further dissect the molecular underpinnings of *D. odorifera*, we retrieved nucleotide and protein data for 9 plant species from the NCBI and Ensembl databases, including *A. duranensis, A. thaliana, Cajanus cajan, E. grandis, G. max, M. domestica, M. truncatula, P. trichocarpa*, and *Vitis vinifera* (Supplementary Table S1). To remove redundancy caused by alternative splicing variations, we retained only gene models at each gene locus that encoded the longest protein sequence. To exclude putative fragmented genes, genes encoding protein sequences shorter than 30 amino acids were filtered out. The OrthoMCL (Ortholog Groups of Protein Sequences, RRID:SCR_007839) v2.0 [45] software was used to cluster genes into gene families with the parameter “-inflation 1.5.” A total of 27,195 gene families were constructed, 9,108 of which were common among species (Supplementary Fig. S11). In addition, we identified 12,092 gene families shared among 5 Fabaceae species while 577 gene families were unique to *D. odorifera* (Fig. 2A and B). The 1,211 species-specific genes in the unique families were significantly overrepresented in the categories of regulation of replication and repair, such as mismatch repair, DNA replication, nucleotide-excision repair, and homologous recombination (Supplementary Fig. S12).

Protein sequences from 390 single-copy gene families were used for phylogenetic tree reconstruction. MUSCLE (MUSCLE, RRID:SCR_011812) v3.8.31 [46] was used to generate multiple sequence alignments for protein sequences in each single-copy family with default parameters. The alignments of each family were concatenated into a super alignment matrix to reconstruct the phylogenetic tree via the maximum likelihood method using RAxML v8.2.12 [47] (RAxML, RRID:SCR_006086). Divergence time between species was estimated using MCMCtree in PAML (PAML, RRID:SCR_014932) v1.3.1 [48]. The parameters used for MCMCtree analysis were as follows: burn-in = 10,000, sample-number = 100,000, sample-frequency = 2. Divergence time for *A. thaliana* and *P. trichocarpa, G. max* and *C. cajan, G. max* and *M. truncatula, G. max* and *M. domestica, A. thaliana*, and *V. vinifera* from the TimeTree database [49] was used as the calibration point. The results indicated that the split between *D. odorifera* and *A. duranensis* occurred ~40.3 million years ago (Mya) (Supplementary Fig. S14).

### Gene family expansion and contraction

We identified expanded and contracted gene families using CAFÉ (CAFÉ, RRID:SCR_005983) v3.1 [50], which uses a random birth and death model to study gains and losses in gene families across a user-specified phylogeny. There were 41 and 214 gene families that appear to have expanded and contracted, respectively (Fig. 2C). The expanded gene families were significantly clustered in 15 KEGG pathways, including replication and repair, environmental adaptation, signal transduction, secondary metabolites, and carbohydrate metabolism (Supplementary Fig. S13).

### Whole-genome duplication analysis

Whole-genome duplication (WGD) analysis was performed by searching for collinearity with the *D. odorifera* genome using MCScan (MCScan, RRID:SCR_017650) v0.8 software [51]. Repeated gene pairs located in internal collinear segments were processed for sequence alignment analysis. The 4-fold degenerate transversion rate (4DTV) values were calculated and used to construct a frequency distribution map for all repeated gene pairs. In addition, MCscan v0.8 was also used to examine collinearity between *D. odorifera* and *P. trichocarpa, D. odorifera* and *A. thaliana, D. odorifera* and *C. cajan*, and *D. odorifera* and *G. max*. The 4DTV values of orthologous gene pairs in the collinear segment were calculated and used to construct a frequency distribution map. The 4DTV plot indicated that after the ancient so-called γ WGD event shared by core eudicots [52], *D. odorifera* had undergone a new round of WGD (Fig. 2D). The approximate time of WGD was ~66.12 and ~146.93 Mya, respectively.

## Conclusion

In this study, we presented the genome of *D. odorifera* and described its genetic attributes. The final chromosome-level genome is almost the most consecutive to date in the Fabaceae family. This high-quality genome could be another model reference sequence for researching the protection and rational utilization of forest. Furthermore, the genome makes it possible to design resequencing studies to discuss the effect of genetic structure on important economic traits.

## Availability of Supporting Data and Materials

Supporting data and materials are available in the *GigaScience* GigaDB database [53], with the raw DNA sequencing data deposited in the NCBI SRA database under BioProject accession number PRJNA613774 and BioSample accession number SAMN14419398. Raw RNA sequencing data can be found via BioProject accession number PRJNA552194.

## Additional Files

Supplementary Table S1. Latin and abbreviated names used in various tables and figures.

Supplementary Table S2. Pacbio data quality assessment.

Supplementary Table S3. Statistics of 17-mer analysis.

Supplementary Table S4. Mapping results of Illumina paired-end reads with short insert sizes.

Supplementary Table S5. Genome completeness evaluated by CEGMA.

Supplementary Table S6. Genome completeness evaluated by BUSCO.

Supplementary Table S7. Summary of Hi-C clustering.

Supplementary Table S8. Summary of Hi-C assembly.

Supplementary Table S9. The genome assembly statistics by each technique.

Supplementary Table S10. Transposable elements (TEs) predicted by each method.

Supplementary Table S11. The sequencing statistics of RNAseq clean data.

Supplementary Table S12. The RNAseq assembly quality statistics.

Supplementary Table S13. Summary of gene predictions.

Supplementary Table S14. Number of genes with homologs or functional classifications based on different databases.

Supplementary Table S15. Number of all types of noncoding RNAs.

Supplementary Figure S1. Cross section of *D. odorifera* wood.

Supplementary Figure S2. Frequency distribution of 17-mers in the *D. odorifera* genome.

Supplementary Figure S3. The K-mer distribution of ~25X Illumina paired-end reads using GenomeScope based on k value of 21.

Supplementary Figure S4. Sequencing depth based on the mapping of paired-end reads with short insert sizes via HiSeq.

Supplementary Figure S5. GC depth plot and sequencing depth of the Illumina sequencing.

Supplementary Figure S6. Genome-wide Hi-C heat map.

Supplementary Figure S7. Scaffold N50 statistics of 13 plant genomes that belong to Fabaceae family and other related species.

Supplementary Figure S8. Distribution of the divergence rate of each type of TE in *D. odorifera*.

Supplementary Figure S9. The evidences supporting gene prediction.

Supplementary Figure S10. Comparison of the distribution of five features in the final gene set for the eight plants.

Supplementary Figure S11. Statistics of gene families in the 10 species examined.

Supplementary Figure S12. Pathway enrichment analysis of 577 *D. odorifera*specific gene families.

Supplementary Figure S13. Pathway enrichment analysis of expanded gene families in *D. odorifera*.

Supplementary Figure S14. Estimation of divergence times.

giaa084_GIGA-D-20-00067_Original_Submission

giaa084_GIGA-D-20-00067_Revision_1

giaa084_Response_to_Reviewer_Comments_Original_Submission

giaa084_Reviewer_1_Report_Original_Submission Caroline Belser -- 4/13/2020 Reviewed

giaa084_Reviewer_1_Report_Revision_1 Caroline Belser -- 5/22/2020 Reviewed

giaa084_Reviewer_2_Report_Original_Submission Debasis Chattopadhyay -- 4/17/2020 Reviewed

giaa084_Supplemental_Files

## Abbreviations

4DTV: 4-fold degenerate transversion rate; bp: base pairs; BLAST: Basic Local Alignment Search Tool; BUSCO: Benchmarking Universal Single-Copy Orthologs; BWA: Burrows-Wheeler Aligner; CAFÉ: Computational Analysis of Gene Family Evolution; CEGMA: Core Eukaryotic Genes Mapping Approach; CTAB: cetyltrimethylammonium bromide; Gb: gigabase pairs; GEM: Gel Bead in Emulsion; GO: Gene Ontology; HW: heartwood; IUCN: International Union for Conservation of Nature; LTR: long terminal repeat; Mb: megabase pairs; Mya: million years ago; NCBI: National Center for Biotechnology Information; PacBio: Pacific Biosciences; PASA: Program to Assemble Spliced Alignments; RAxML: Randomized Axelerated Maximum Likelihood; SMRT: single-molecule real-time; SRA: Sequence Read Archive; WGD: whole-genome duplication.

## Competing Interests

The authors declare that they have no competing financial interests.

## Funding

This work was supported by the Fundamental Research Funds for the Central Non-profit Research Institution of Chinese Academy of Forestry(CAFYBB2017ZX001-4, CAFYBB2016QB009, CAFYBB2017SY021), National Natural Science Foundation of China (31500537), and the National Key Research and Development Program of China (2016YFD0600601).

## Authors' Contributions

Z.H. designed the sequencing strategy. X.L., N.Z., Z.Y., and Z.C. prepared and analysed the samples. Y.N., J.Lian, and J.Li. drew the figures. J.Li. and Z.H. wrote the manuscript with input from other co-authors. D.X. was responsible for project administration.
