# Supplementary material for: The chromosome-level draft genome of Dalbergia odorifera
Source: Gigascience. 2020 Aug 18;9(8):giaa084. doi: 10.1093/gigascience/giaa084 (PMC7433187; doi:10.1093/gigascience/giaa084)
Supplement: giaa084_GIGA-D-20-00067_Original_Submission [file giaa084_giga-d-20-00067_original_submission.pdf]

GigaScience

Chromosome-level genome of the Dalbergia odorifera provide insight into the antimicrobial properties of its heartwood

--Manuscript Draft--

**Manuscript Number:**	GIGA-D-20-00067
**Full Title:**	Chromosome-level genome of the Dalbergia odorifera provide insight into the antimicrobial properties of its heartwood
**Article Type:**	Research
**Funding Information:**	the Fundamental Research Funds for the Central Non-profit Research Institution of Chinese Academy of Forestry (CAFYBB2016QB009) — Dr Zhou Hong<br>the Fundamental Research Funds for the Central Non-profit Research Institution of Chinese Academy of Forestry (CAFYBB2017SY021) — Dr Ningnan Zhang<br>National Natural Science Foundation of China (31500537) — Dr Zhou Hong<br>the Fundamental Research Funds for the Central Non-profit Research Institution of Chinese Academy of Forestry (CAFYBB2017ZX001-4) — Prof Daping Xu<br>The National Key Research and Development Program of China (2016YFD0600601) — Prof Daping Xu
**Abstract:**	Dalbergia odorifera T. Chen (Leguminosae) is of high medicinal and commercial value due to its officinal, insect-proof, durable heartwood. Here, we present a chromosome-scale genome assembly of D. odorifera obtained based on Pacific Bioscience single-molecule real-time sequencing, Illumina paired-end sequencing, 10X Genomics linked-reads, and Hi-C data. We assembled 97.68% of the 653.45 Mb D. odorifer a genome at chromosomal level resolution with scaffold N50 of 56.16 Mb and predicted 30,310 protein-coding genes in the assembly. The combination results of comparative genomic, transcriptomic, and metabolite indicated that immune response related genes, such as ATRAD17, POLH and SUMM2 might closely relate to the formation of heartwood with antimicrobial activities. These findings shed light on one aspect of the formation mechanism of high-quality durable heartwood and provide valuable genomic resources for the improvement of D. odorifera and other timber trees.
**Corresponding Author:**	Daping Xu<br>Research Institute of Tropical Forestry Chinese Academy of Forestry<br>Guangzhou, CHINA
**Corresponding Author Secondary Information:**	
**Corresponding Author's Institution:**	Research Institute of Tropical Forestry Chinese Academy of Forestry
**Corresponding Author's Secondary Institution:**	
**First Author:**	Daping Xu
**First Author Secondary Information:**	
**Order of Authors:**	Daping Xu<br>Zhou Hong<br>Jiang Li<br>Xiaojin Liu<br>Jinmin Lian

Powered by Editorial Manager® and ProduXion Manager® from Aries Systems Corporation

	Ningnan Zhang
	Zengjiang Yang
	Yongchao Niu
	Zhiyi Cui
**Order of Authors Secondary Information:**	
**Additional Information:**	
**Question**	**Response**
Are you submitting this manuscript to a special series or article collection?	No
**Experimental design and statistics**<br><br>Full details of the experimental design and statistical methods used should be given in the Methods section, as detailed in our Minimum Standards Reporting Checklist. Information essential to interpreting the data presented should be made available in the figure legends.<br><br>Have you included all the information requested in your manuscript?	Yes
**Resources**<br><br>A description of all resources used, including antibodies, cell lines, animals and software tools, with enough information to allow them to be uniquely identified, should be included in the Methods section. Authors are strongly encouraged to cite Research Resource Identifiers (RRIDs) for antibodies, model organisms and tools, where possible.<br><br>Have you included the information requested as detailed in our Minimum Standards Reporting Checklist?	Yes
**Availability of data and materials**<br><br>All datasets and code on which the conclusions of the paper rely must be either included in your submission or	Yes

deposited in publicly available repositories (where available and ethically appropriate), referencing such data using a unique identifier in the references and in the “Availability of Data and Materials” section of your manuscript.<br><br>Have you have met the above requirement as detailed in our Minimum Standards Reporting Checklist?	

Manuscript Click here to download Manuscript Research Article.docx

Chromosome-level genome of the *Dalbergia odorifera* provide insight into the antimicrobial properties of its heartwood

Zhou Hong[1,†], Jiang Li[2,†], Xiaojin Liu[1], Jinmin Lian[2], Ningnan Zhang[1], Zengjiang Yang[1], Yongchao Niu[2], Zhiyi Cui[1], Daping Xu[1,*]

[1] State Key Laboratory of Tree Genetics and Breeding, Research Institute of Tropical Forestry, Chinese Academy of Forestry, Guangzhou 510520, China, [2] Biozeron Shenzhen Inc., Shenzhen 518000, China

[*]Correspondence address. Daping Xu, State Key Laboratory of Tree Genetics and Breeding, Research Institute of Tropical Forestry, Chinese Academy of Forestry, Guangzhou 510520, China. Tel: +86 020 87033626; E-mail: gzfsrd@163.com.

[†]These authors contributed equally to manuscript preparation.

Abstract

Dalbergia odorifera T. Chen (Leguminosae) is of high medicinal and commercial value due to its officinal, insect-proof, durable heartwood. Here, we present a chromosome-scale genome assembly of *D. odorifera* obtained based on Pacific Bioscience single-molecule real-time sequencing, Illumina paired-end sequencing, 10X Genomics linked-reads, and Hi-C data. We assembled 97.68% of the 653.45 Mb *D. odorifer*a genome at chromosomal level resolution with scaffold N50 of 56.16 Mb and predicted 30,310 protein-coding genes in the assembly. The combination results of

comparative genomic, transcriptomic, and metabolite indicated that immune response related genes, such as *ATRAD17, POLH* and *SUMM2* might closely relate to the formation of heartwood with antimicrobial activities. These findings shed light on one aspect of the formation mechanism of high-quality durable heartwood and provide valuable genomic resources for the improvement of *D. odorifera* and other timber trees.

Key words: *Dalbergia odorifera* T. Chen; *de novo* sequencing; comparative genomics; transcriptomics; metabonomics; heartwood

Background

Dalbergia odorifera is a medium-sized evergreen tree belonging to the Leguminosae family. *D. odorifera* originated in Hainan, China and was gradually introduced and cultivated in Guangdong, Fujian, Zhejiang, Guangxi, and Yunnan, China. *D. odorifera* is an ideal biological model to study the mechanism underlying high-quality heartwood (HW) formation due to its insect-proof, durable, fragrant, beautiful HW [1]. HW is defined as the central wood layers of a tree (Additional file 2: Fig. S1). This tissue, containing nonliving cells and nonfunctioning xylem tissue, can affect tree health, with broader implications for forest health [2]. The natural durability of wood as well as the biological, technological, and aesthetic parameters of wood and wood products depend on the presence, quality, and quantity of HW, which is strongly affected by external stimuli [3]. Flavonoids, which are the major compounds found in

D. odorifera, are a main class of secondary metabolites that strongly affect various properties of HW, including durability and the color of wood products [2]. Besides, flavonoids are crucial for plant resistance against pathogenic bacteria and fungi, and flavonoid production can be induced by fungal invasion [4]. It is worth noting that carbohydrates can also affect flavonoid accumulation and the formation of phenolic extractives, which contribute to the natural durability of wood during HW formation [5]. Apart from its excellence as a wood product, the HW of *D. odorifera*, which is known as “JiangXiang” in traditional Chinese medicine, has been included in the Chinese Pharmacopoeia for decades and is widely used to dissipate stasis, stop bleeding, and relieve pain. *D. odorifera* HW is also used to treat blood stagnation syndrome, ischemia, swelling, necrosis, and rheumatic pain in Korea [6]. Due to its great medicinal and commercial value, *D. odorifera* is becoming more and more rare: Only limited numbers of individuals are found in parts of their original habitat, with highly fragmented populations present in the remaining forests of Hainan Island [7]. It is worth noting that *D. odorifera* has been listed on the IUCN’s (International Union for Conservation of Nature) red list by the World Conservation Monitoring Centre (WCMC) since 1998 (http://www.iucnredlist.org/details/32398/0).

Despite the commercial interest and increasing demand for *D. odorifera*, the lack of a genome sequence for this species has limited analysis of the mechanism underlying HW formation in *D. odorifera*, which has seriously hampered conservation and breeding efforts. Advances in sequencing and assembly technology have made it possible to obtain chromosome-level reference genome sequences for organisms once

thought to be intractable, including forest trees, which are highly heterozygous and have repetitive genomes.

In this study, we used PacBio data, Hi-C data, and 10X Genomics linked-reads data to assemble a high-quality reference genome of *D. odorifera*. We then performed comparative genomics analysis based on precise, complete genome data. In addition, transcriptome and metabolite samples were obtained from 3 different heights (Additional file 2: Fig. S15) and each height group included 3 biological replicates. By combining multi-omics data, we try to provide insights into durable HW formation of *D. odorifera*. Moreover, this high-quality genome provided here will facilitate genetic research of *D. odorifera* and related species.

Data Description

To obtain the whole-genome sequences of *D. odorifera*, genomic DNA was extracted from leaf tissues using the cetyltrimethylammonium bromide (CTAB) method. Paired-end (PE) library with an insert size of 350bp was constructed according to the manufacturer’s instructions (Illumina, San Diego, CA). Besides, a 10X Genomics linked-read library was also constructed and sequenced on the Illumina HiSeq 4000 platform. Single-molecule real-time (SMRT) sequencing of long reads on a Pacific Biosciences (PacBio) Sequel platform was used to assist the subsequent *de novo* genome assembly. In summary, ~192 and 74 gigabase (Gb) reads were generated on Illumina platforms and PacBio platforms respectively. Raw sequence data generated by the Illumina platform were filtered by the following criteria: filtered reads with

adapters, filtered reads with N bases more than 10%, and filtered reads with low-quality bases (≤ 5) more than 50%. For the PacBio data, subreads were filtered with the default parameters. The details about assembly, gene prediction, and annotation can be found in the Materials and Methods.

RESULTS

Genome assembly

We utilized a comprehensive strategy for *D. odorifera* genome assembly. We obtained 397.72× coverage of the ~653.45-Mb *D. odorifera* genome, whose size was estimated by k-mer distribution analysis (Additional file 2: Fig. S2), including PacBio single-molecule long reads (~103.67× coverage), HiSeq short-insert size paired-end reads (~113.06× coverage), and 10X Genomics reads (~180.99× coverage; Additional file 1: Table S3). We performed PacBio-only contig assembly using the FALCON package, followed by Quiver polishing. We then used HiSeq paired-end reads with short insert sizes to improve the contig assembly with Pilon. Finally, we used 10X Genomics reads to create scaffolds from the contigs to yield a 638.26 Mb assembly with an N50 contig size of 6.18 Mb and an N50 scaffold size of 7.25 Mb (Additional file 1:Table S4).

To assess the quality of the genome assembly, we mapped paired-end reads with short insert sizes onto the assembly. Nearly 98% of these reads could be mapped to the *D. odorifera* draft genome, with the genome coverage reaching 99.63% (Additional file 1: Table S5). In addition, of the 1,440 genes identified using Embryophyta

Benchmarking Universal Single-Copy Orthologs (BUSCO), 92.2% complete and 1.7% partial genes were identified in the assembled genome (Additional file 1: Table S7), and 235 of the 248 genes identified using Core Eukaryotic Genes Mapping Approach (CEGMA) were retrieved in the assembly (Additional file 1: Table S6). Using a Hi-C library (~238.52× coverage), we improved the scaffold N50 value to 56.16 Mb, with the longest scaffold reaching 79.61 Mb. The final reference assembly comprised 10 chromosome-scale pseudomolecules, with 638.28 Mb of the 653.45 Mb genome assembled in total (Table 1; Additional file 1: Table S9). The N50 of this assembly is almost the best in the Leguminosae family that have been recently completed (Additional file 2: Fig. S6). These results indicate that the newly generated *D. odorifera* genome is of high quality and we successfully assembled the major genic regions of this precious plant species.

Genome annotation

We identified and masked 54.17% of the assembly as repeat regions (Additional file 1: Table S11). Long terminal repeats (LTRs) were the most abundant (comprising 37.7% of the genome), followed by DNA transposons (9.16% of the genome; Additional file 1: Table S12). We then annotated the *D. odorifera* genome using a comprehensive annotation strategy combining RNA-Seq-derived transcript evidence, *de novo* gene prediction, and sequence similarity to proteins from 7 related plant species. Using this strategy, we annotated 30,311 protein-coding genes, with an average coding sequence length of 1.12 kb and an average of 4.93 exons per gene (Additional file 1: Table S13).

Nearly 96% of the genes were supported by homology searches and/or the presence of expressed transcripts (Additional file 2: Fig. S8) with gene components similar to those of related species (Additional file 2:Fig. S9).

To verify the sensitivity of our gene predictions, we performed statistical analysis of core genes using BUSCO. Our gene predictions recovered 1,325 of the 1,440 (92%) highly conserved core proteins in the embryophyte lineage, including 74% single-copy genes and 18% duplicated genes (Additional file 1: Table S14). Of the predicted genes, 92.6% (28,069 genes) were annotated using functional databases (Additional file 1: Table S15). In addition, we identified 1,914 noncoding RNAs, including 747 miRNAs, 112 rRNA, 582 tRNAs, and 473 snRNAs (Additional file 1: Table S16).

Genome evolution

To investigate the phylogenic positions and to further dissect the molecular underpinnings of *D. odorifera*, we retrieved nucleotide and protein data for 9 plant species from the NCBI and Ensembl databases, including *Arachis duranensis*, *Arabidopsis thaliana*, *Cajanus cajan*, *Eucalyptus grandis*, *Glycine max*, *Malus domestica*, *Medicago truncatula*, *Populus trichocarpa*, and *Vitis vinifera*. Gene family clustering via OrthoMCL identified 27,195 gene families, 9,108 of which were common among species (Additional file 2: Fig. S10). In addition, we identified 12,092 gene families shared among 5 legume species and 577 gene families that were unique to *D. odorifera* (Fig. 2A; Fig. 2B). The species-specific genes were significantly overrepresented in the categories of regulation of replication and repair, such as

mismatch repair, DNA replication, nucleotide-excision repair, and homologous recombination (Additional file 2: Fig. S11). Besides, the phenylpropanoid biosynthesis pathway, which belonged to the biosynthesis of secondary metabolites category, was also significantly enriched. Correspondingly, Gene Ontology (GO) enrichment analysis revealed 31 genes associated with response to stress, 30 genes associated with ADP binding and 12 genes associated with defense response (Additional file 1: Table S17).

We performed phylogenetic analysis of 390 single-copy orthologous genes in the genomes of the 10 plant species to investigate the evolutionary trajectory of *D. odorifera*. The results indicated that the split between *D. odorifera* and *A. duranensis* occurred approximately 40.3 million years ago (Additional file 2: Fig. S13). The 4-fold degenerate transversion rate (4DTV) plot indicated that after the ancient so-called γ whole-genome duplication (WGD) event shared by core eudicots [8], *D. odorifera* had undergone a new round of WGD (Fig. 2D). In addition, *D. odorifera* diverged from the ancestor of *A. thaliana* and *P. trichocarpa* and then separated from *G. max* and *C. cajan*, which was consistent with the results of phylogenetic analysis (Fig. 2C). There were 41 and 214 gene families that appear to have expanded and contracted, respectively (Fig. 2C). The expanded gene families were significantly clustered in 15 KEGG pathways, including replication and repair, environmental adaptation, signal transduction, secondary metabolites, and carbohydrate metabolism (Additional file 2: Fig. S12). By integrating the secondary metabolite and carbohydrate related genes, we determined that the shared genes belong to the

β-glucosidase family (Fig. 4A). Many secondary metabolites are stored in the inactive glycosylated form. These glyconjugates are activated by hydrolysis of the β-glucosidic bond by β-glucosidases [9]. Meanwhile, GO enrichment analysis revealed that 42 genes involved in defense response, 47 genes involved in response to stress, 57 genes involved in response to stimulus, 17 involved in carbohydrate binding, and 31 genes involved in carbohydrate metabolic process (Additional file 1: Table S18). Further functional analysis of the expanded gene families revealed 44 genes containing an NB-ARC domain and/or NBS-LRR domain. These types of genes played important roles in disease resistance in plants [10].

Using the branch-site likelihood ratio test, we identified 58 positively selected genes (PSGs). Notably, the GO terms of the PSGs were mainly related to DNA repair, DNA metabolic process, response to stress, single-organism cellular process and single-organism metabolic process (Additional file 1: Table S19).

Genomic insights into durable HW formation

To explore the genomics insights into durable HW formation, we performed transcriptomics and metabolomics experiments derived from three different heights of *D. odorifera* vascular cambium, with each height group including 3 biological replicates (Additional file 2: Fig. S14). A total of 51.59 G Illumina reads were generated and 94.26% of which were mapped to the assembled genome (Additional file 1: Table S20). The expression levels of 24,950 genes (82.32% of total annotated genes) were detected and quantified in at least one sample (Additional file 2: Fig. S16).

In total, 21,452 genes were found to be co-expression and 780 genes were only expressed in samples taken from the base of the tree (Additional file 2: Fig. S17). We observed that the plant-pathogen interaction pathway was the most significantly enriched pathway among these 780 genes (Additional file 2: Fig. S18). In the comparison of lower samples between higher samples, 6764, 7554 and 3003 differentially expressed genes (DEGs) were obtained respectively (Additional file 1: Table S21). The DEGs mainly involved in phenylalanine metabolism, phenylpropanoid biosynthesis, photosynthesis, photosynthesis - antenna proteins, and plant hormone signal transduction (Additional file 1: Table S22). 894 and 339 compounds were detected in the ESI^+ and ESI^- modes, separately (Additional file 2: Fig. S19). According to the KEGG database, 10 (16) compounds related to flavone and flavonoid (Fig. 4B) in the ESI^+ and ESI^- modes, such as 3′,4′,5,7-tetrahydroxyflavone, 5,7-dihydroxy-4′-methoxyflavone, and biochanin A. The selection of variables responsible for the differences was performed through statistical analysis as described in materials and methods. A total of 119 (42), 175 (45), and 80 (2) differential compounds were selected between the comparison of lower samples and higher samples in the ESI^+ (ESI^-) mode, separately (Additional file 2: Fig. S21). We found that the significant enrichment pathway of differential compounds was closely related to the results of transcriptome or experimental background, such as photosynthesis, plant-pathogen interaction, flavonoid biosynthesis and regulation of autophagy (Additional file 1:Table S22). What’s more, we observed that 2 PSGs, namely, *POLH* and *ATRAD17*, were significantly differentially expressed genes at the

comparison group of base between top, and middle between top (Fig. 3A). *ATRAD17* and *POLH* are conserved in plants and animals and involved in DNA damage responses [11]. These 2 PSGs had interaction relationship and *POLH* was nearly at the core of PSGs interaction network according to result of string [12] database (Fig. 3B). The expression of *SUMM2* at transcriptome level was significantly positive correlation with pinocembrin and naringenin at metabolome level in ESI^+ and ESI^- modes separately. Even more interesting was that *SUMM2* defined as DEG, pinocembrin and naringenin were also selected as significantly differentially expressed metabolites at the comparison group of middle between top. Pinocembrin, one of flavonoid, could improve the biological functions of endothelial progenitor cells (EPCs) resulting in inhibiting atherosclerosis [13]. Similar to pinocembrin, naringin also belonged to flavonoids, was found to display strong anti-inflammatory and antioxidant activities [14]. *SUMM2* function as an immune receptor, could activate defense responses [15], suggesting that immune response might play a positive role in the accumulation of flavones resulting in promoting the formation of durable HW (Fig. 4A).

Discussion

D. odorifera is an excellent model for study the durable HW formation due to its high density, antibacterial and bactericidal properties. In addition, flavonoids which are the main compounds in *D. odorifera* have anti-oxidative, anti-inflammatory, anti-mutagenic, and anti-carcinogenic properties associated with their capacity to

modulate the activities of key cellular enzymes [16]. In this study, we presented the genome of *D. odorifera* firstly via PacBio single-molecule long reads, HiSeq short-insert size paired-end reads and Hi-C technologies. The final chromosome-level genome is almost the most consecutive to date in the Leguminosae family. Besides, this high-quality genome could be another model reference sequence for researching the protection and rational utilization of forest. Practically 96% of the predicted genes were supported by homologous evidence and/or transcriptome evidence, indicating the great potentials to be used for investigating gene function. In general, the proportion of HW to total wood decreases with increasing tree height [17]. In addition to comparative genomics, we designed three different heights of vascular cambium transcriptome and metabolome experiments so as to investigate the genomic insights into antimicrobial HW formation. The analysis results of multi-omics indicate that DNA damage, immune response and plant-pathogen interaction genes may affect the formation of antimicrobial HW, such as *ATRAD17, POLH* and *SUMM2*. The activation of DNA damage responses is an intrinsic component of the plant immune response, an important mechanism against pathogens [18]. We hypothesize that the immune response is a key factor could not be ignored for antimicrobial HW formation. Previous study reported that the increase of free sterols in the HW may be due to a possible participation in defense mechanisms against pathogens [19]. The finding by employing cDNA microarray demonstrated that the genes coding for defense and cell rescue are up-regulated during the process of HW formation [20, 21]. Additionally, ESTs from drying and discoloring sapwood of *Cryptomeria* revealed a preponderance

of genes coding for secondary metabolism and defense-related proteins [22]. These findings are consistent with our speculation. Understanding the formation of HW in *D. odorifera* can not only be useful for the efficient and sustainable utilization of this precious resource, but also provide the theoretical basis for forest development and protection. However, it’s a pity that the database of metabonomics is far from perfect, the mining of metabonomics is not deep enough. With the continuous improvement of the database, more HW formation mechanisms can be obtained in the future. Furthermore, the genome makes it possible to design re-sequencing studies to discuss the impact of genetic structure on important economic traits.

Materials and Methods

Sampling and DNA extraction

A 13-year-old *D. odorifera* tree from Guangzhou, China (113.38°E, 23.19°N) was used for genome sequencing. Genomic DNA was isolated from leaf tissue using a DNA purification kit (Qiagen, Valencia, CA, USA). For transcriptome and metabolome, vascular cambium samples were obtained from different height of the tree, namely, 0.3 meters above the ground (base), 1.8 meters above the ground (middle), and 3.5 to 4 meters above the ground (top; Additional file 2: Fig. S14). Each group has three biological replicates. All samples were immediately frozen in liquid nitrogen and stored at -80°C until use.

Sequencing, assembly, and evaluation

Pacific Bioscience (PacBio) single-molecule real-time sequencing, Illumina paired-end sequencing, 10X Genomics linked-reads, and Hi-C technology were utilized for genome sequencing and assembly. A paired-end Illumina sequencing library was constructed with an insert size of 350 bp. Sequencing was carried out on the Illumina HiSeq 4000 platform according to the manufacturer's instructions. In addition, a 10X Genomics linked-read library was also constructed and sequenced on the Illumina HiSeq 4000 platform. The PacBio reads were sequenced using the Sequel platform. Finally, the Hi-C library was prepared following standard procedures.

FALCON v2.0.5 [23] assembler was used to assemble the contigs of the *D. odorifera* genome. Then the genome was polished using Quiver v5.0 [24]. Illumina short reads were used to produce a more contiguous genome with fewer errors with Pilon v1.18 [25]. The PacBio contigs were connected to super scaffolds based on 10X Genomics linked-read data using fragScaff v140324 [26] software. Finally, Hi-C technology was used to anchor the contigs to pseudomolecules as described by Bickhart et al.[27]. To assess the completeness of the assembled *D. odorifera* genome, BUSCO v3.0 analysis was performed by searching against embryophyta BUSCO [28]. The completeness of the *D. odorifera* genome was also assessed using CEGMA v2.5 [29].

Genome annotation

We used a combination of homology searching and *ab initio* prediction to study the repetitive sequences in the *D. odorifera* genome. For homology-based prediction, we

used RepeatMasker v4.0.7 [30] and RepeatProteinMask v4.0.7 to search against Repbase (http://www.girinst.org/repbase). For *ab initio* prediction, we used Tandem Repeats Finder v4.07b [31], LTR_FINDER v 1.07 [32], RepeatModeler v1.0.8, Piler v1.0 [33], and RepeatScout v1.0.5 [34] with default parameters. Four types of noncoding RNAs (microRNAs, transfer RNAs, ribosomal RNAs, and small nuclear RNAs) were annotated using tRNAscan-SE v1.23 and the Rfam database v9.1 [35]. Three approaches were employed to predict the protein-coding genes in the *D. odorifera* genome, including homologous comparison, *ab initio* prediction, and RNA-Seq-based annotation. For homologous comparison, the reference protein sequences from the Ensembl database and NCBI database for seven species, including *A. thaliana* (ftp://ftp.ensemblgenomes.org/pub/plants/release-32), *P. trichocarpa* (ftp://ftp.ensemblgenomes.org/pub/plants/release-32), *E. grandis* (GCF_000612305.1), *M. truncatula* (ftp://ftp.ensemblgenomes.org/pub/plants/release-32), *A. duranensis* (GCF_000817695.2), *M. domestica* (GCF_000148765.1), and *G. max* (ftp://ftp.ensemblgenomes.org/pub/plants/release-32) were aligned against the *D. odorifera* genome using a TBLASTN v2.2.15 [36] search with E-value 1e-5 in the "-F F" option. All BLAST hits were concatenated after filtering low-quality records. The sequence of each candidate gene was further extended upstream and downstream by 1,000 bp to represent the entire gene region. Gene structures were predicted using GeneWise v2.4.1 [37]. Genes predicted in a homology-based manner were viewed as the "Homology-set". RNA reads were assembled using Trinity v2.0 [38], and the assembled sequences were aligned against the *D. odorifera* genome using Program to

Assemble Spliced Alignment (PASA), which assembles effective alignments into gene structures [39]. Gene models created by PASA v2.3.3 [40] were viewed as the PASA-T-set (PASA Trinity set). We simultaneously employed five tools for *ab initio* prediction, including Augustus v3.2.3 [41], GeneID v1.4.4 [42], GeneScan v1.0) [43], GlimmerHMM v3.52 [44], and SNAP v2006-07-28 [45]. Notably, the parameters were computationally optimized by training a set of high-quality protein sequences derived from the PASA gene models. At the same time, RNA-Seq reads were aligned to the *D. odorifera* genome using TopHat v2.0.9 [46] with default parameters, then the mapped reads were assembled into gene models with Cufflinks v2.2.1[47]. Finally, the nonredundant reference gene set was generated using EvidenceModeler v1.1.1. Weights for each type of evidence were as follows: PASA-T-set > Homology-set > Cufflinks set > Augustus > GeneID = SNAP = GlimmerHMM = GeneScan. PASA was also employed to identify untranslated regions and to obtain information about alternative splicing variation. Gene functions were assigned to the translated protein-coding genes using BLASTP v2.2.15 [48] based on their highest match to proteins in the SwissProt [49] and NR databases. Motifs and domains in the protein-coding genes were retrieved by performing InterProScan v4.7 [50] searches against six protein databases: ProDom, PRINTS, Pfam, SMART, PANTHER, and PROSITE. GO [51] terms for each gene were detected from the corresponding InterPro entries. Genes were aligned against the KEGG database [52]. The pathways in which the genes might be involved were deduced from the matching genes in KEGG.

Phylogenetic analysis and estimation of species divergence time

To investigate the phylogenic position of *D. odorifera*, we retrieved nucleotide and protein data for *A. duranensis* (GCF_000817695.2), *A. thaliana* (ftp://ftp.ensemblgenomes.org/pub/plants/release-32), *C. cajan* (GCF_000340665.1), *E. grandis* (GCF_000612305.1), *G. max* (ftp://ftp.ensemblgenomes.org/pub/plants/release-32), *M. domestica* (GCF_000148765.1), *M. truncatula* (ftp://ftp.ensemblgenomes.org/pub/plants/release-32), *P. trichocarpa* (ftp://ftp.ensemblgenomes.org/pub/plants/release-32), and *V. vinifera* (ftp://ftp.ensemblgenomes.org/pub/plants/release-32/) from public databases. Only the gene model that encoded the longest protein sequence was retained in order to remove redundancy caused by alternative splicing variations, and genes encoding protein sequences shorter than 30 amino acids were filtered out. All-against-all BLASTP was employed to identify the similarity among the filtered protein sequences with an E-value cutoff of 1e-7. The OrthoMCL v2.0 [53] software was used to cluster genes into gene families with the parameter "-inflation 1.5". Protein sequences from 390 single-copy gene families were used for constructing phylogenetic trees. MUSCLE v3.8.31 [54]was employed to generate multiple sequence alignments for protein sequences in each single-copy family with default parameters. The alignments of each family were concatenated into a super alignment matrix to reconstruct the phylogenetic tree via the maximum likelihood method. Divergence time among species was estimated using MCMCtree in PAML v1.3.1 [55]. Divergence time for *A. thaliana* and

P. trichocarpa, *G. max* and *C. cajan*, *G. max* and *M. truncatula*, *G. max* and *M. domestica*, *A. thaliana*, and *V. vinifera* from the TimeTree database (http://www.timetree.org/) was used as the calibration point.

Gene family expansion and contraction analyses

We identified expanded and contracted gene families using CAFÉ v3.1 [56], which employs a random birth and death model to study gains and losses in gene families across a user-specified phylogeny. The global parameter λ, which describes both the gene birth (λ) and death ($\mu = -\lambda$) rate across all branches in the tree for all gene families, was estimated using the maximum likelihood method. A conditional P-value was calculated for each gene family, and families with conditional P-values less than the threshold (0.05) were considered to have a notable gain or loss. We identified branches responsible for low overall P-values of significant families.

Detection of positively selected genes

We calculated Ka/Ks ratios for all single-copy orthologs of *D. odorifera* and the nine other species. Firstly, the MUSCLE v3.8.31 [54] program was carried out for multiple sequence alignment for protein sequences in a single-copy gene family with default parameters. Then Gblocks v0.91b [57] was used to remove ambiguously aligned blocks. Finally, “codeml” in the PAML v4.5 package with the branch-site model was used to detect positively selected genes (PSGs).

WGD analysis

WGD analysis was performed by searching for collinearity with the *D. odorifera* genome using MCscan v0.8 (http://chibba.agtec.uga.edu/duplication/mcscan/) software [58]. Repeat gene pairs located in internal collinear segments were processed for sequence alignment analysis. The 4-fold degenerate transversion rate (4DTV) values were calculated and used to construct a frequency distribution map for all repeated gene pairs. Besides, MCscan v0.8 was also employed to examine collinearity between *D. odorifera* and *P. trichocarpa*, *D. odorifera* and *A. thaliana*, *D. odorifera* and *C. cajan,* and *D. odorifera* and *G. max*. The 4DTV values of orthologous gene pairs in the collinear segment were calculated and used to construct a frequency distribution map.

RNA sequencing analysis

Trimming and quality control of the raw paired-end reads were performed using Trimmomatic v0.33 [59] with the parameters "SLIDINGWINDOW:4:15 MINLEN:75." The clean reads were aligned to the reference genome using HISAT2 v2.0.5 [60] software. Then the expression level of each transcript was calculated using the fragments per kilobase of exon per million mapped reads (FPKM) [61] method. Cuffdiff (http://cufflinks.cbcb.umd.edu/) was used to identify differentially expressed genes (DEGs) between different samples and the DEGs between two samples were selected using the following criteria: fold change ≥ 2 and FDR ≤ 0.05. To explore the functions of the DEGs, GO functional enrichment and KEGG pathway analysis were

performed using Goatools v0.8.12 (https://github.com/tanghaibao/Goatools) and KOBAS v3.0 [62]. DEGs were considered to be significantly enriched in GO terms and metabolic pathways when their Bonferroni-corrected P-value was <0.05.

Metabolite analyses

Metabolite extraction

Each 50-mg tissue sample was placed into an Eppendorf tube. After the addition of 1,000 μL of extract solvent (acetonitrile-methanol-water, 2:2:1, containing internal standard 1 μg/mL), the samples were vortexed for 30 s, homogenized at 45 Hz for 4 min, and sonicated for 5 min in an ice-water bath; the homogenization and sonication steps were repeated three times. The samples were incubated at -20°C for 1 h and centrifuged at 12,000 rpm and 4°C for 15 min. The resulting supernatants were transferred to liquid chromatography-mass spectrometry (LC-MS) vials and stored at -80°C prior to UHPLC-QE Orbitrap/MS analysis.

LC-MS/MS analysis

LC-MS/MS analysis was performed using an UHPLC system (1290, Agilent Technologies) with a UPLC HSS T3 column (2.1 mm × 100 mm, 1.8 μm) coupled to Q Exactive (Orbitrap MS, Thermo). The mobile phase A was 0.1% formic acid in water for positive ion detection and 5 mmol/L ammonium acetate in water for negative ion detection, and the mobile phase B was acetonitrile. The elution gradient was as follows: 0 min, 1% B; 1 min, 1% B; 8 min, 99% B; 10 min, 99% B; 10.1 min, 1% B; 12 min, 1%

B. The flow rate was 0.5 mL/min. The injection volume was 2 μL. The QE mass spectrometer was used to acquire MS/MS spectra on an information-dependent basis (IDA) during an LC-MS experiment. In this mode, the acquisition software (Xcalibur 4.0.27, Thermo) continuously evaluates the full scan survey MS data as it collects and triggers the acquisition of MS/MS spectra depending on preselected criteria. ESI source conditions were as follows: sheath gas flow rate of 45 Arb, Aux gas flow rate of 15 Arb, capillary temperature of 320°C, full MS resolution of 70,000, MS/MS resolution of 17,500, collision energy of 20/40/60 eV for the NCE model, and spray voltage of 3.8 kV (positive mode) or -3.1 kV (negative mode).

Data preprocessing and annotation

MS raw data files were converted to mzML format using ProteoWizard and processed with the R package XCMS v 3.2. The preprocessing results generated a data matrix consisting of the retention time (RT), mass-to-charge ratio (m/z) values, and peak intensity. OSI-SMMS (v 1.0; Dalian Chem Data Solution Information Technology Co. Ltd.) was used for peak annotation after XCMS data processing with an in-house MS/MS database.

Multivariate statistical analysis

PCA modeling was used to examine the aggregation degree of the QC samples. Different groups were then analyzed by the multivariate analysis methods PCA, partial least square discriminant analysis (PLS-DA), and orthogonal projections to latent

structures discriminant analysis (OPLS-DA). Differential metabolites were screened out based on the VIP (variable importance in the projection) value of the PLS-DA model (VIP ≥ 1) and independent sample t-test ($P \leq 0.05$). The METLIN online database was searched for accurate molecular weight comparisons to qualitatively differentiate the metabolites. $[M+H]^+$ and $[M+Na]^+$ were selected in positive mode and $[M-H]^-$ and $[M+FA-H]^-$ were selected in negative mode. The mass error value was set to 20 ppm.

Availability of supporting data and materials

The *D. odorifera* genome assembly and the sequencing data used for *de novo* whole-genome assembly are available from the China National GeneBank (CNGB) Nucleotide Sequence Archive (CNSA) under accession number CNP0000528. RNA sequence data from this article can be found in the Sequence Read Archive (SRA) under ID number SRP212606.

Additional files

Additional file 1: A Word file with Table S1-S23.

Additional file 2: A Word file with Fig. S1-S21.

Abbreviations

HW: heartwood; IUCN: International Union for Conservation of Nature; SMRT: Single-molecule real-time; PacBio: Pacific Biosciences; CTAB:

cetyltrimethylammonium bromide; BUSCO: Benchmarking Universal Single-Copy Orthologs; CEGMA: Core Eukaryotic Genes Mapping Approach; LTRs: Long terminal repeats; GO: Gene Ontology; 4DTV: 4-fold degenerate transversion rate; WGD: whole-genome duplication; PSGs: positively selected genes; DEGs: differentially expressed genes; *COMT*: caffeic acid 3-*O*-methyltransferase; *CCR*: cinnamoyl-CoA reductase; *bglB*: β-glucosidase; *E1.11.1.7*: peroxidase; *REF1*: coniferyl-aldehyde dehydrogenase; *bglX*: β-glucosidase; *E2.1.1.104*: caffeoyl-CoA *O*-methyltransferase; *HCT*: shikimate *O*-hydroxycinnamoyltransferase; *AAE*: acetylajmaline esterase; *IF7GT*: isoflavone 7-*O*-glucosyltransferase; *IF7MAT*: isoflavone 7-*O*-glucoside-6″-*O*-malonyltransferase; *HIDH*: 2-hydroxyisoflavanone dehydratase; *AMY*: amyA, malS α-amylase; *bglB*: β-glucosidase; *ALDH*: aldehyde dehydrogenase (NAD+); *E1.10.3.3*: L-ascorbate oxidase; *USP*: UDP-sugar pyrophosphorylase.

Competing interests

The authors declare they have no competing financial interests.

Funding

This work was supported by the Fundamental Research Funds for the Central Non-profit Research Institution of Chinese Academy of Forestry (CAFYBB2017ZX001-4), the Fundamental Research Funds for the Central Non-profit Research Institution of Chinese Academy of Forestry

(CAFYBB2016QB009), the Fundamental Research Funds for the Central Non-profit Research Institution of Chinese Academy of Forestry (CAFYBB2017SY021), National Natural Science Foundation of China (31500537) and the National Key Research and Development Program of China (2016YFD0600601).

Author contributions

Z.H. designed the sequencing strategy. X.L., N.Z., Z.Y., and Z.C. prepared and analysed the samples. Y.C.N, J.M.L., and J.L. draw the figures. J.L. and Z.H. wrote the manuscript with input from other co-authors. D.P.X. was responsible for project administration.

References

1. Sun S, Zeng X, Zhang D, Guo S: **Diverse fungi associated with partial irregular heartwood of Dalbergia odorifera**. *Scientific Reports* 2015, **5**:8464.
2. Celedon J, Bohlmann J: **An extended model of heartwood secondary metabolism informed by functional genomics**. *Tree physiology* 2017, **38**:1-9.
3. Kampe A, Magel E: **New Insights into Heartwood and Heartwood Formation**; 2013.
4. Mierziak J, Kostyn K, Kulma A: **Flavonoids as Important Molecules of Plant Interactions with the Environment**. *Molecules (Basel, Switzerland)* 2014, **19**:16240-16265.
5. Park C, Kim Y, Li X, Kim H-H, Arasu M, Al-Dhabi N, Lee S-Y, Park SU: **Influence of Different Carbohydrates on Flavonoid Accumulation in Hairy Root Cultures of Scutellaria baicalensis**. *Natural product communications* 2016, **11**:799-802.

6. Kang T-H, Tian Y-H, Kim Y-C: **Isoliquiritigenin : A Competitive Tyrosinase Inhibitor from the Heartwood of Dalbergia odorifera**. *Biomolecules and Therapeutics* 2005, **13**.

7. Liu F, Hong Z, Jia H, Zhang N, Liu X, Yang Z, Lu M: **Genetic Diversity of the Endangered Dalbergia odorifera Revealed by SSR Markers**. *Forests* 2019, **10**:18.

8. Bowers J, Chapman B, Rong J, Paterson A: **Bowers JE, Chapman BA, Rong JK, Paterson AH. Unravelling angiosperm genome evolution by phylogenetic analysis of chromosomal duplication events. Nature 422: 433-438**. *Nature* 2003, **422**:433-438.

9. Baba SA, Vishwakarma RA, Ashraf NJJoBC: **Functional characterization of CsBGlu12, a β-glucosidase from Crocus sativus provides insights into its role in abiotic stress through accumulation of antioxidant flavonols**. 2017, **292**(11):jbc.M116.762161.

10. Biezen E, Jones J: **The NB-ARC domain: A novel signalling motif shared by plant resistance gene products and regulators of cell death in animals**. *Current biology : CB* 1998, **8**:R226-227.

11. Nikitaki Z, Pavlopoulou A, Holá M, Donà M, Michalopoulos I, Balestrazzi A, Angelis K, Georgakilas A: **Bridging Plant and Human Radiation Response and DNA Repair through an In Silico Approach**. *Cancers* 2017, **9**:65.

12. Szklarczyk D, Gable A, Lyon D, Junge A, Wyder S, Huerta-Cepas J, Simonovic M, Doncheva N, Morris J, Bork P *et al*: **STRING v11: protein-protein association networks with increased coverage, supporting functional discovery in genome-wide experimental datasets**. *Nucleic acids research* 2018, **47**.

13. Yang N, Qin S, Wang M, Chen B, Yuan N, Fang Y, Yao S, Jiao P, Yu Y, Zhang Y *et al*: **Pinocembrin, a major flavonoid in propolis, improves the biological functions of EPCs derived from rat bone marrow through the PI3K-eNOS-NO signaling pathway**. *Cytotechnology* 2013, **65**(4):541-551.

14. Alam MA, Subhan N, Rahman MM, Uddin SJ, Reza HM, Sarker SD: **Effect of citrus flavonoids, naringin and naringenin, on metabolic**

syndrome and their mechanisms of action. *Adv Nutr* 2014, **5**(4):404-417.

15. Zhang Z, Liu Y, Huang H, Gao M, Wu D, Kong Q, Zhang Y: **The NLR protein SUMM2 senses the disruption of an immune signaling MAP kinase cascade via CRCK3**. *EMBO Rep* 2017, **18**(2):292-302.
16. Panche A, Diwan A, Chandra S: **Flavonoids: An overview**. *Journal of Nutritional Science* 2016, **5**.
17. Herrero de Aza C, Turrión MB, Pando V, Bravo F: **Carbon in heartwood, sapwood and bark along the stem profile in three Mediterranean Pinus species**. *Annals of Forest Science* 2011, **68**(6):1067.
18. Li H, Zhou Y, Zhang Z: **Network Analysis Reveals a Common Host–Pathogen Interaction Pattern in Arabidopsis Immune Responses**. *Frontiers in Plant Science* 2017, **8**.
19. Hillinger C, Höll W, Ziegler H: **Lipids and lipolytic enzymes in the trunkwood ofRobinia pseudoacacia L. during heartwood formation**. *Trees* 1996, **10**(6):376-381.
20. Yang J, Kamdem DP, Keathley DE, Han K-H: **Seasonal changes in gene expression at the sapwood—heartwood transition zone of black locust (Robinia pseudoacacia) revealed by cDNA microarray analysis**. *Tree Physiology* 2004, **24**(4):461-474.
21. Huang Z, Tsai C-J, Harding SA, Meilan R, Woeste K: **A Cross-species Transcriptional Profile Analysis of Heartwood Formation in Black Walnut**. *Plant Molecular Biology Reporter* 2010, **28**(2):222-230.
22. Yoshida K, Futamura N, Nishiguchi M: **Collection of expressed genes from the transition zone of Cryptomeria japonica in the dormant season**. *Journal of Wood Science* 2012, **58**(2):89-103.
23. Pendleton M, Sebra R, Pang A, Ummat A, Franzén O, Rausch T, Stütz A, Stedman W, Anantharaman T, Hastie A *et al*: **Assembly and diploid architecture of an individual human genome via single-molecule technologies**. *Nature Methods* 2015, **12**.
24. Chin C-S, Alexander D, Marks P, Klammer A, Drake J, Heiner C, Clum A, Copeland A, Huddleston J, Eichler E *et al*: **Nonhybrid, finished microbial genome assemblies from long-read SMRT sequencing data**.

Nature methods 2013, **10**.

25. Walker BJ, Abeel T, Shea T, Priest M, Abouelliel A, Sakthikumar S, Cuomo CA, Zeng Q, Wortman J, Young SK *et al*: **Pilon: an integrated tool for comprehensive microbial variant detection and genome assembly improvement**. *PLoS One* 2014, **9**(11):e112963.
26. Mostovoy Y, Levy-Sakin M, Lam J, Lam E, Hastie A, Marks P, Lee J, Chu C, Lin C, Dzakula Z *et al*: **A Hybrid Approach for de novo Human Genome Sequence Assembly and Phasing**. *Nature methods* 2016, **13**.
27. Bickhart DM, Rosen BD, Koren S, Sayre BL, Hastie AR, Chan S, Lee J, Lam ET, Liachko I, Sullivan ST *et al*: **Single-molecule sequencing and chromatin conformation capture enable de novo reference assembly of the domestic goat genome**. *Nature Genetics* 2017, **49**(4):643-650.
28. Simão F, Waterhouse R, Ioannidis P, Zdobnov E: **BUSCO: Assessing genome assembly and annotation completeness with single-copy orthologs**. *Bioinformatics (Oxford, England)* 2015, **31**.
29. Parra G, Bradnam K, Korf I: **CEGMA: A pipeline to accurately annotate core genes in eukaryotic genomes**. *Bioinformatics (Oxford, England)* 2007, **23**:1061-1067.
30. Bergman CM, Quesneville H: **Discovering and detecting transposable elements in genome sequences**. *Briefings in Bioinformatics* 2007, **8**(6):382-392.
31. Benson G: **Tandem repeats finder: a program to analyze DNA sequences**. *Nucleic acids research* 1999, **27**(2):573-580.
32. Xu Z, Wang H: **LTR-FINDER: An efficient tool for the prediction of full-length LTR retrotransposons**. *Nucleic acids research* 2007, **35**:W265-268.
33. Edgar R, Myers E: **PILER: Identification and classification of genomic repeats**. *Bioinformatics (Oxford, England)* 2005, **21 Suppl 1**:i152-158.
34. Price A, Jones N, Pevzner P: **Price, A.L., Jones, N.C. & Pevzner, P.A. De novo identification of repeat families in large genomes. Bioinformatics 21, i351-i358**. *Bioinformatics (Oxford, England)* 2005, **21 Suppl 1**:i351-358.
35. Griffiths-Jones S, Moxon S, Marshall M, Khanna A, Eddy S, Bateman A:

Rfam: Annotating Non-Coding RNAs in Complete Genomes. *Nucleic acids research* 2005, **33**:D121-124.
36. Gertz E, Yu Y-K, Agarwala R, Schaffer A, Altschul S: **Composition-based statistics and translated nucleotide searches: Improving the TBLASTN module of BLAST**. *BMC biology* 2006, **4**:41.
37. Birney E, Clamp M, Durbin R: **GeneWise and Genomewise**. *Genome Res* 2004, **14**(5):988-995.
38. Grabherr M, Haas B, Yassour M, Levin J, Thompson D, Amit I, Adiconis X, Fan L, Raychowdhury R, Zeng Q *et al*: **Trinity: reconstructing a full-length transcriptome without a genome from RNA-Seq data**. *Nature biotechnology* 2013, **29**:644.
39. Haas B, Salzberg S, Zhu W, Pertea M, Allen J, Orvis J, White O, Buell C, Wortman J: **Automated eukaryotic gene structure annotation using EVidenceModeler and the Program to Assemble Spliced Alignments**. *Genome biology* 2008, **9**:R7.
40. Haas BJ, Delcher AL, Mount SM, Wortman JR, Smith RK, Jr., Hannick LI, Maiti R, Ronning CM, Rusch DB, Town CD *et al*: **Improving the Arabidopsis genome annotation using maximal transcript alignment assemblies**. *Nucleic acids research* 2003, **31**(19):5654-5666.
41. Stanke M, Morgenstern B: **AUGUSTUS: A web server for gene prediction in eukaryotes that allows user-defined constraints**. *Nucleic acids research* 2005, **33**:W465-467.
42. Guigó R, Knudsen S, Drake N, Smith T: **Prediction of gene structure**. *Journal of molecular biology* 1992, **226**:141-157.
43. Aggarwal G, Ramaswamy R: **Ab initio gene identification: Prokaryote genome annotation with GeneScan and GLIMMER**. *Journal of Biosciences* 2002, **27**(1):7-14.
44. Majoros W, Pertea M, Salzberg S: **TigrScan and GlimmerHMM: Two open source ab initio eukaryotic gene-finders**. *Bioinformatics (Oxford, England)* 2004, **20**:2878-2879.
45. Korf I: **Gene finding in novel genomes**. *BMC Bioinformatics* 2004, **5**(1):59.
46. Kim D: **TopHat2: accurate alignment of transcriptomes in the presence**

of insertions, deletions and gene fusions. *Genome Biol* 2013, **14**:R36.

47. Trapnell C, Roberts A, Goff L, Pertea G, Kim D, Kelley D, Pimentel H, Salzberg S, Rinn J, Pachter L: **Differential gene and transcript expression analysis of RNA-Seq experiments with TopHat and Cufflinks**. *Nature protocols* 2012, **7**:562-578.
48. Pevsner J: **Basic Local Alignment Search Tool (BLAST)**. In., vol. 215; 2009: 100-138.
49. Bairoch A, Apweiler R: **The SWISS-PROT protein sequence data bank and its supplement TrEMBL in 1998**. *Nucleic Acids Research* 1997, **25**:31-36.
50. Jones P, Binns D, Chang H-Y, Fraser M, Li W, McAnulla C, McWilliam H, Maslen J, Mitchell A, Nuka G *et al*: **InterProScan 5: Genome-scale Protein Function Classification**. *Bioinformatics (Oxford, England)* 2014, **30**.
51. Ashburner M, Ball C, Blake J, Botstein D, Butler H, Cherry J, Davis AP, Dolinski K, Dwight S, Eppig J *et al*: **Gene ontology: tool for the unification of biology. The Gene Ontology Consortium**. *Nature genetics* 2000, **25**:25-29.
52. Ogata H, Goto S, Sato K, Fujibuchi W, Bono H, Kanehisa M: **KEGG: kyoto Encyclopedia of Genes and Genomes**. *Nucleic acids research* 1999, **27**:29-34.
53. Li L, Stoeckert C, Roos D: **OrthoMCL: Identification of Ortholog Groups for Eukaryotic Genomes**. *Genome research* 2003, **13**:2178-2189.
54. Edgar R: **MUSCLE: Multiple Sequence Alignment with High Accuracy and High Throughput**. *Nucleic acids research* 2004, **32**:1792-1797.
55. Yang Z: **PAML 4: Phylogenetic Analysis by Maximum Likelihood**. *Molecular Biology and Evolution* 2007, **24**(8):1586-1591.
56. Hahn M, Demuth J, Han S-G: **Accelerated Rate of Gene Gain and Loss in Primates**. *Genetics* 2007, **177**:1941-1949.
57. Talavera G, Castresana J: **Improvement of Phylogenies after Removing Divergent and Ambiguously Aligned Blocks from Protein Sequence Alignments**. *Systematic biology* 2007, **56**:564-577.
58. Tang H, Bowers J, Wang X, Ming R, Alam M, Paterson A: **Synteny and**

Collinearity in Plant Genomes. *Science (New York, NY)* 2008, **320**:486-488.

59. Bolger A, Lohse M, Usadel B: **Trimmomatic: A Flexible Trimmer for Illumina Sequence Data**. *Bioinformatics (Oxford, England)* 2014, **30**.
60. Kim D, Langmead B, Salzberg S: **HISAT: A fast spliced aligner with low memory requirements**. *Nature methods* 2015, **12**.
61. Roberts A, Trapnell C, Donaghey J, Rinn J, Pachter L: **Improving RNA-seq expression estimates by correcting for fragment bias. Genome Biol 12(3):R22**. *Genome biology* 2011, **12**:R22.
62. Xie C, Huang J, Ding Y, Wu J, Dong S, Kong L, Gao G, Li C-Y, Wei L: **KOBAS 2.0: A web server for annotation and identification of enriched pathways and diseases**. *Nucleic acids research* 2011, **39**:W316-322.

Table1 Statistics for the *D. odorifera* genome

Assembly feature	Value
Estimated genome size (by *k*-mer analysis)	653.45 Mb
Number of scaffolds	480
Contig N50	6.19 Mb
Scaffold N50	56.16 Mb
Longest Scaffold	79.61 Mb
Assembly length	638.26 Mb
Assembly % of genome	97.68
Repeat region % of assembly	54.17
Predicted gene models	30,310
Average coding sequence length	1,121.36 bp
Average exons per gene	4.93

Circos plot of the D. odorifera genome.

Click here to download Figure Figure1.jpg

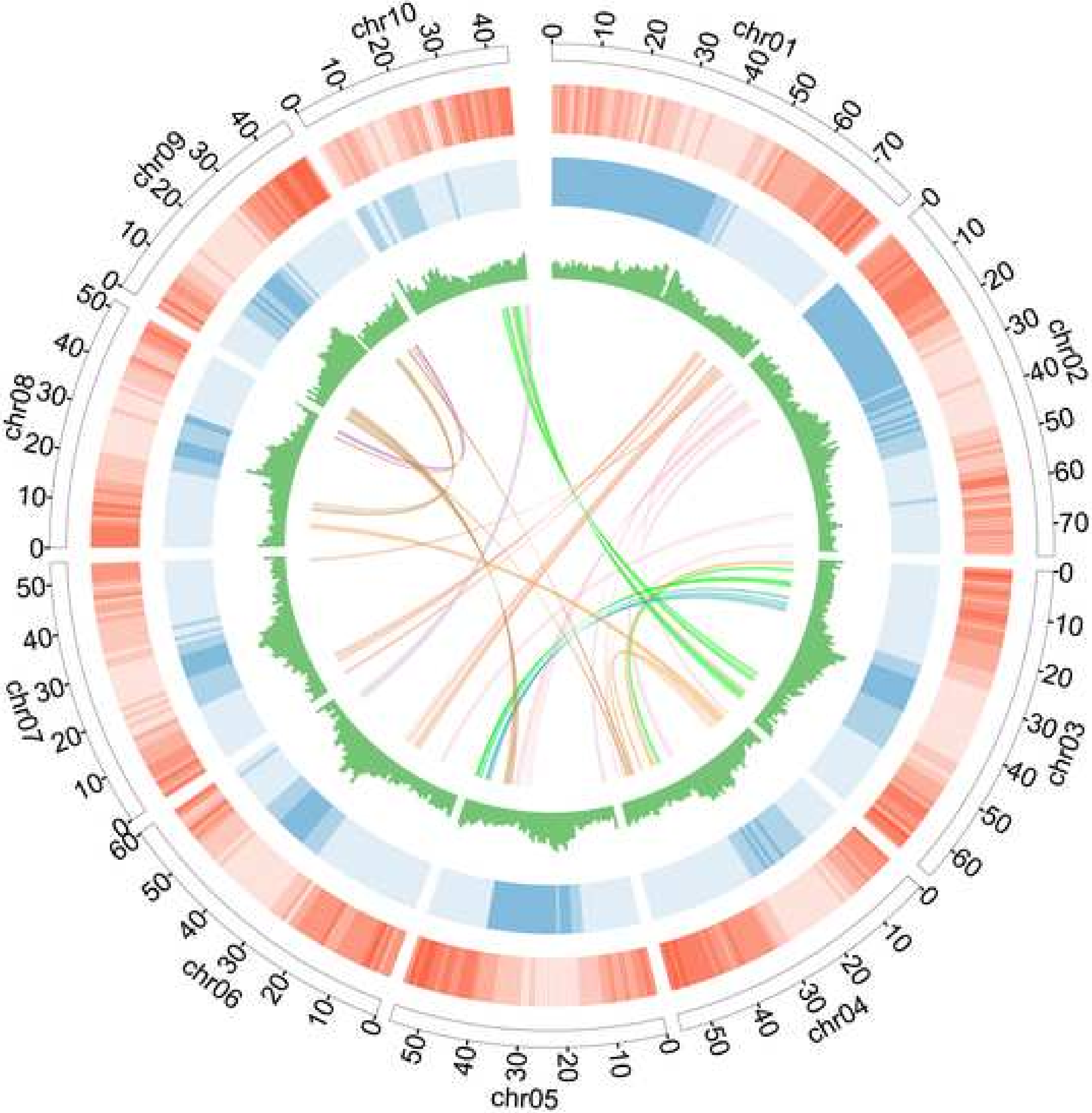

Evolution of the D. odorifera genome.

Click here to download Figure Figure2.jpg

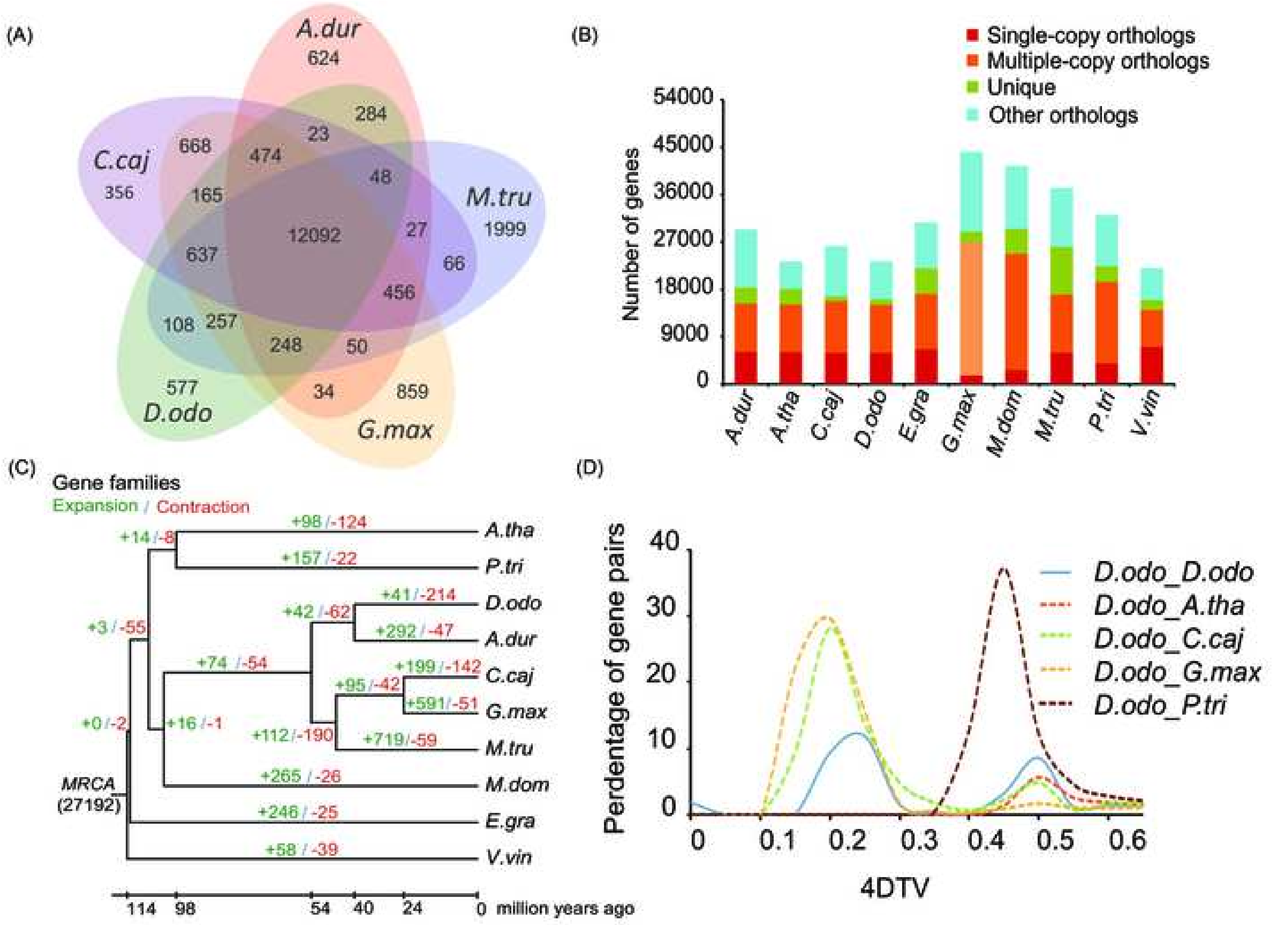

Heat map and network of PSGs.

[Click here to download Figure Figure3.jpg]

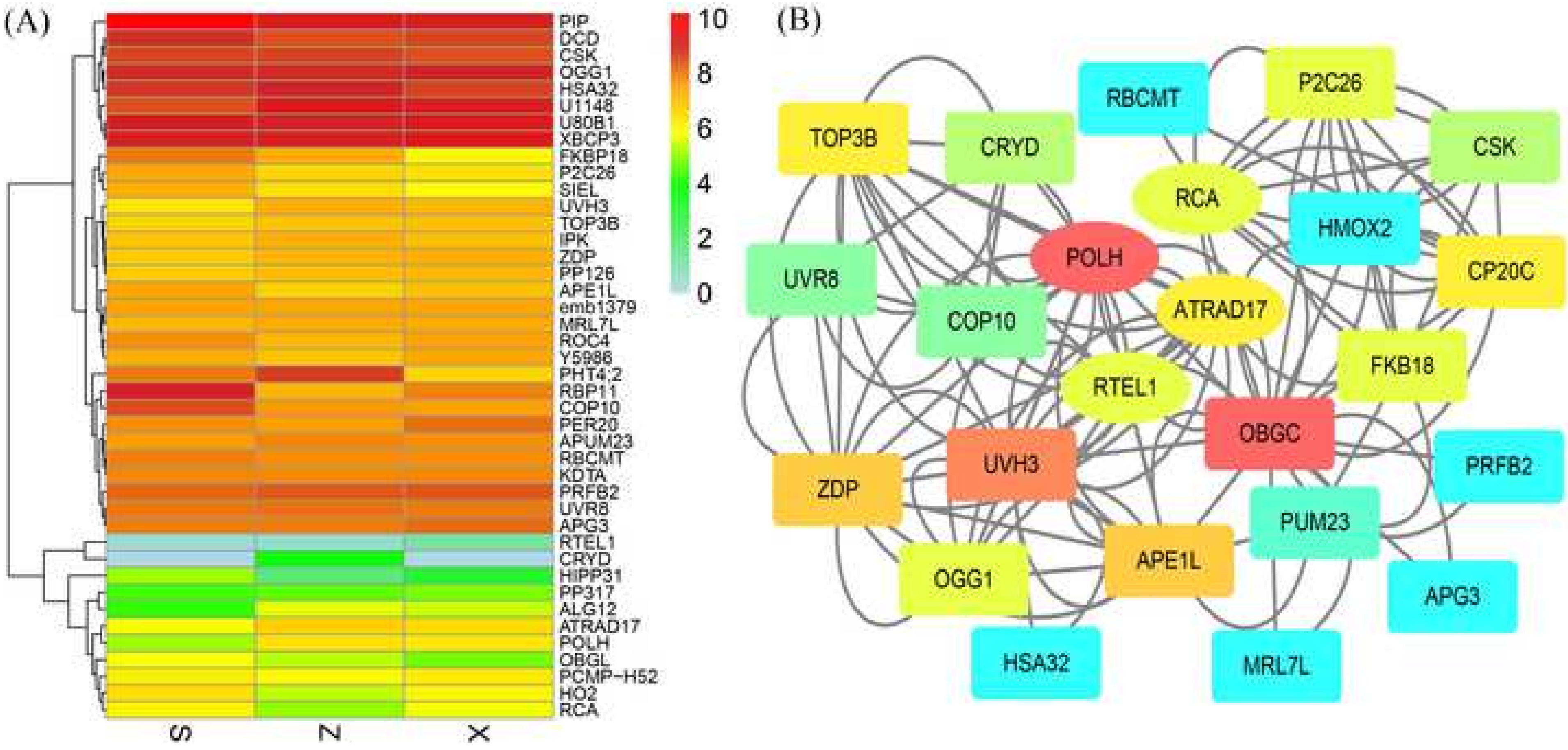

Genes and compounds related to flavones and isoflavones.

Click here to download Figure Figure4.jpg

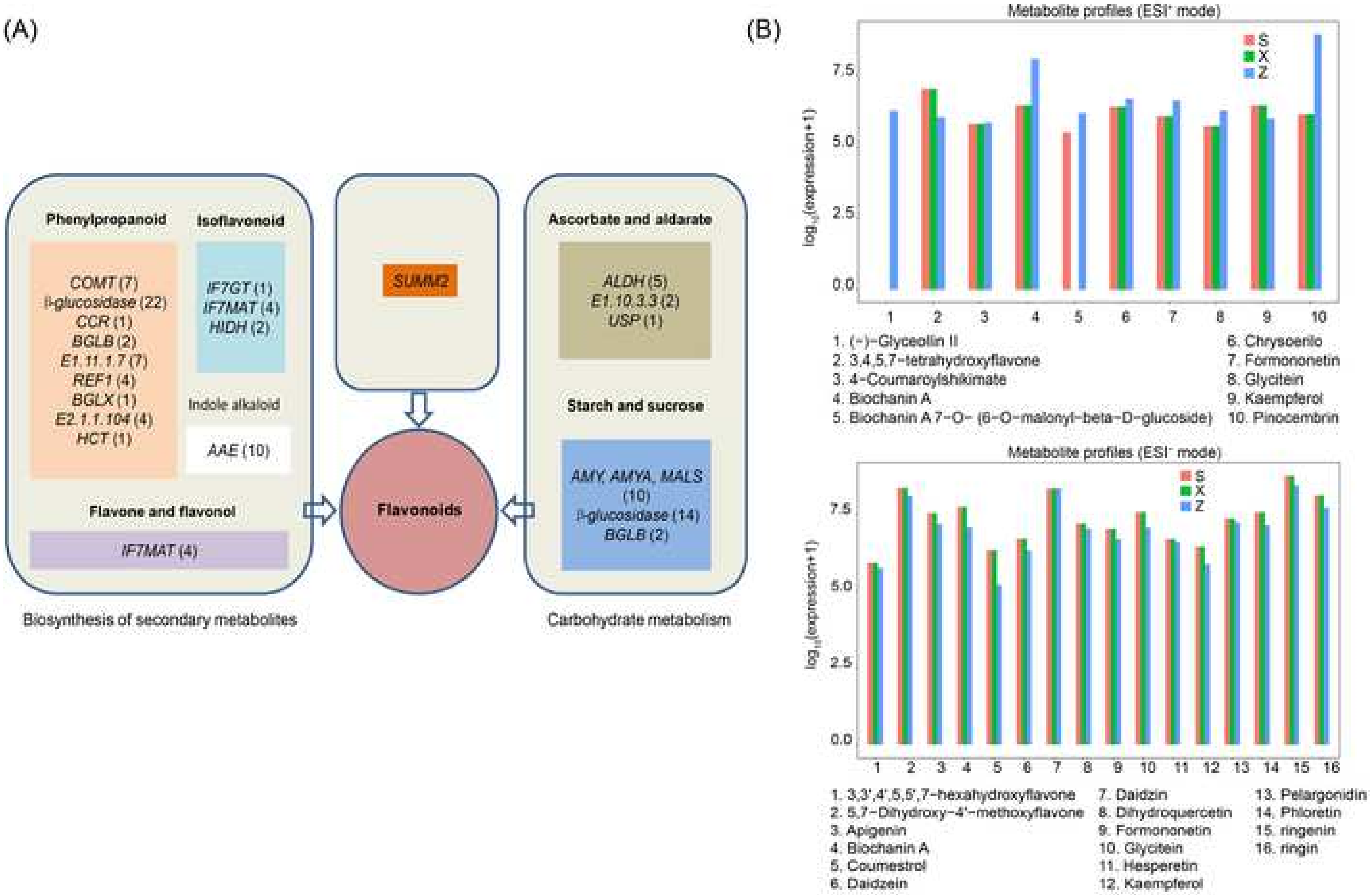

Supplementary Material 1

Click here to access/download
Supplementary Material
Additional file 1.docx

Click here to access/download
Supplementary Material
Additional file 2.docx
