# Supplementary material for: The chromosome-level draft genome of Dalbergia odorifera
Source: Gigascience. 2020 Aug 18;9(8):giaa084. doi: 10.1093/gigascience/giaa084 (PMC7433187; doi:10.1093/gigascience/giaa084)
Supplement: giaa084_GIGA-D-20-00067_Revision_1 [file giaa084_giga-d-20-00067_revision_1.pdf]

GigaScience

The chromosome-level draft genome of Dalbergia odorifera

--Manuscript Draft--

**Manuscript Number:**	GIGA-D-20-00067R1
**Full Title:**	The chromosome-level draft genome of Dalbergia odorifera
**Article Type:**	Data Note

Funding Information:

the Fundamental Research Funds for the Central Non-profit Research Institution of Chinese Academy of Forestry (CAFYBB2017ZX001-4)	Prof Daping Xu
the Fundamental Research Funds for the Central Non-profit Research Institution of Chinese Academy of Forestry (CAFYBB2016QB009)	Dr Zhou Hong
the Fundamental Research Funds for the Central Non-profit Research Institution of Chinese Academy of Forestry (CAFYBB2017SY021)	Dr Ningnan Zhang
National Natural Science Foundation of China (31500537)	Dr Zhou Hong
The National Key Research and Development Program of China (2016YFD0600601)	Prof Daping Xu

Abstract:

Backgroud: Dalbergia odorifera T. C. Chen (Fabaceae) is an IUCN red listed tree. This tree is of high medicinal and commercial value due to its officinal, insect-proof, durable heartwood. However, there is a lack of genome reference, which has hindered development of studies on the heartwood formation. Findings: We presented the first chromosome-scale genome assembly of D. odorifera obtained based on Illumina paired-end sequencing, Pacific Bioscience single-molecule real-time sequencing, 10× Genomics linked-reads, and Hi-C technology. We assembled 97.68% of the 653.45 Mb D. odorifera genome with scaffold N50 and contig sizes of 56.16 Mb and 5.92 Mb, respectively. Ten super-scaffolds corresponding to the 10 chromosomes were assembled with the longest scaffold reaching 79.61 Mb. Repetitive elements account for 54.17% of the genome, and 30,310 protein-coding genes were predicted from the genome, of which approximately 92.6% were functionally annotated. The phylogenetic tree showed that D. odorifera diverged from the ancestor of A. thaliana and P. trichocarpa , and then separated from G. max and C. cajan . Conclusions: We sequence and reveal the first chromosome-level de novo genome of D. odorifera . These studies provide valuable genomic resources for the research of heartwood formation in D. odorifera and other timber trees. The high-quality assembled genome can also be used as reference for comparative genomics analysis and future population genetic studies of D. odorifera .

**Corresponding Author:**	Daping Xu<br>Research Institute of Tropical Forestry Chinese Academy of Forestry<br>Guangzhou, CHINA
**Corresponding Author Secondary Information:**	
**Corresponding Author's Institution:**	Research Institute of Tropical Forestry Chinese Academy of Forestry
**Corresponding Author's Secondary Institution:**	
**First Author:**	Daping Xu
**First Author Secondary Information:**	
**Order of Authors:**	Daping Xu
	Zhou Hong

Powered by Editorial Manager® and ProduXion Manager® from Aries Systems Corporation

	Jiang Li
	Xiaojin Liu
	Jinmin Lian
	Ningnan Zhang
	Zengjiang Yang
	Yongchao Niu
	Zhiyi Cui
**Order of Authors Secondary Information:**	
**Response to Reviewers:**	For a Data Note in-depth analysis is not required, but we do require some validation. Comparative genomics is a good way to do this and is usually recommended, but you do not necessarily need to do any biological experimentation to meet this requirement.<br>Answers: Thanks for your reminding. The collinearity analyses have been done using jcvi method (https://github.com/tanghaibao/jcvi). Firstly, we find out all the published genome of legumes on the website: https://www.plabipd.de/plant_genomes_pa.ep. According to the divergence time from the website: http://www.timetree.org/, Arachis duranensis was the closest legume specie to Dalbergia odorifera. So we make a collinearity analysis of Arachis duranensis between Dalbergia odorifera. While due to the long genetic distance (~40 MYA) and low genome collinearity between Arachis duranensis and Dalbergia odorifera, it is not suitable for verification of Dalbergia odorifera assembly by comparative genomics so far.Since I can't upload the pictures in the column of response to reviewers, I have sent you the collinearity diagram and the document responding to the reviewer's comments by e-mail.<br><br>In addition, please register any new software application in the bio.tools and SciCrunch.org databases to receive RRID (Research Resource Identification Initiative ID) and biotoolsID identifiers, and include these in your manuscript. This will facilitate tracking, reproducibility and re-use of your tool.<br>Answers: Thanks for your reminding. The RRID have been added.<br><br>Line 79. The DNA extraction was performed using the CTAB method. There is no reference for this method. Please add a reference or explain the method.<br>Answers: Thanks for your reminding. The reference has been added.<br><br><br>Line 97. The genome size was estimated by k-mer distribution analysis. Please clarify which method you used ? How did you estimate the heterozygosity rate ? Did you compare your results with results from Genomescope for example ?<br>Answers: The genome size of D. odorifera is estimated with the following formula: genome size = [Num (total k-mer) -Num (erroneous k-mer)]/mean depth of k-mer. 17-mers are counted as 55,992,417,644 from short clean reads. The total count of error k-mers is 449,157,644 and the k-mer depth is 85. Therefore, the genome size of D. odorifera is estimated to be around 653.45 Mb. Besides, we use around 25X Illumina's paired-end sequencing clean read to make GenomeScope analysis. The rate of genome heterozygosity estimated by GenomeScope (http://qb.cshl.edu/genomescope/) is around 0.59% and the genome size is around 623.46 Mb.<br>Our method: Genome size=653.45Mb, Heterozygosity=0.54%,TE=56.65%. The size of the assembled genome=638Mb, TE=54.17%. It's very close to our assessment.<br><br><br>Line 106-121 : The assembly has a good gene completeness and seems to be of high quality. But can you add some evaluations such as KAT histogramms which reflect the completeness of the assembly process. (https://kat.readthedocs.io/en/latest/).<br>Answers: Thanks for your comments. We further validate the assembly by comparing the cleaned read spectrum from two paired-end libraries with the copy number in the assembly using KAT toolkit v2.4.2. The results show that the heterozygous rate is 0.81% and the assembly completeness is 99.73%.<br><br>Line 278. Can you specify the kit used ? What is the quantity of material engaged in

Powered by Editorial Manager® and ProduXion Manager® from Aries Systems Corporation

the preparations ?
Answers: Thanks for your reminding. The library detail information has been added.

Can you detail the preparation of the 10X sequencing library ? What is the quantity of DNA engaged in the library preparation. What is the size distribution of the DAN fragments used ?
Answers: Thanks for your reminding. The 10X library detail information has been added.

Line 293. Please add some informations about the HiC library preparation or add a reference. What is the enzyme used ?
Answers: The enzyme is DpnII and it used cuts at "GATC". The reference has been added.

Lines 298-299. You used the HiC data to organize the scaffolds into pseudo-molecules. Did you detect mis-assemblies after 10X scaffolding ?
Answers: We use the Hi-C data to organize the scaffolds into pseudo-molecules after 10X scaffolding, so we can’t detect mis-assemblies by 10X Genomics link-reads. But according to the results of Hi-C, we could correct the mis-assemblies that may be introduced by 10x scaffolding. The Hi-C interaction heatmap indicate that the clusting result is good.

Line 327. Sequences produced from the RNA sequencing libraries are assembled. Can you detail the RNA extraction method ? Did you extract RNA from the fresh leaves too ?
Answers:We have added detail information about the RNA sequencing libraries, thanks for your comments.

Line 407. You perform analysis on RNA sequencing. But you spoke about replicates (line 193). Did you use the three replicates of each samples in your analysis ? Have you check the reproducibility of the results thanks to the replicates?
Answers: According to the editor’ request, for Data Note in-depth analysis is not required. We have made some adjustments to the manuscript, thanks for your comments.

Typo :
Line 398 : repeated gene pairs
Line 198 : co-expressed instead of co-expression
Line 224 : has instead of was
Answers:Revised, thanks for your comments.

Reviewer #2: The manuscript describes genome assembly, annotation, Transcript sequence of D. odorifera and very preliminary analysis of genome and some of the medicinally important genes. The quality of genome assembly and annotation is very high. However, the other analysis are routine. A big problem for me to assess this manuscript is I could not find the legends of the figures in the main text. The genome assembly statistics table must represent the step-by-step improvement for each sequencing technique. The approximate time of WGD is not mentioned. I did not find any comparative structure genomic analysis between D. odorifera and nearest species (MCScanX?). At the same time, what is the speciality in the sequence of medicinally important genes of D. odorifera vis-a-vis the nearest species that allowed it to produce novel compounds. To publish in Gigascience, I believe some useful biological analysis and information are required. A more informative and biologically oriented manuscript would be suitable for this journal.

Answers: Thanks for your comment. According to the editor’ advice, this manuscript was transferred to Data Note format. We have made some adjustments to the manuscript according to your suggestions. Figure legends has been added to the text, and the statistics of assembly process has been added to the assembly statistics table. The approximate time of WGD was described.

Powered by Editorial Manager® and ProduXion Manager® from Aries Systems Corporation

	The collinearity analyses between Dalbergia odorifera and Arachis duranensis have been done using jcvi method (https://github.com/tanghaibao/jcvi). Firstly, we find out all the published genome of legumes on the website: https://www.plabipd.de/plant_genomes_pa.ep. According to the divergence time from the website: http://www.timetree.org/, Arachis duranensis was the closest legume specie to Dalbergia odorifera at present. So we make a collinearity analysis of Arachis duranensis between Dalbergia odorifera. I can't upload pictures in the column of response to reviewers, so I sent the collinearity figure to the eidtor by e-mail.<br>While due to the long genetic distance (~40 MYA) and low genome collinearity between Arachis duranensis and Dalbergia odorifera, it is not suitable for verification of Dalbergia odorifera assembly by comparative genomics so far.<br>According to the editor' request, for Data Note in-depth analysis is not required. Comparative genomics is usually recommended, but we do not necessarily need to do any biological experimentation to meet this requirement. Thanks again for your valuable comments.
**Additional Information:**	
**Question**	**Response**
Are you submitting this manuscript to a special series or article collection?	No
**Experimental design and statistics**<br><br>Full details of the experimental design and statistical methods used should be given in the Methods section, as detailed in our Minimum Standards Reporting Checklist. Information essential to interpreting the data presented should be made available in the figure legends.<br><br>Have you included all the information requested in your manuscript?	Yes
**Resources**<br><br>A description of all resources used, including antibodies, cell lines, animals and software tools, with enough information to allow them to be uniquely identified, should be included in the Methods section. Authors are strongly encouraged to cite Research Resource Identifiers (RRIDs) for antibodies, model organisms and tools, where possible.<br><br>Have you included the information requested as detailed in our Minimum Standards Reporting Checklist?	Yes

Powered by Editorial Manager® and ProduXion Manager® from Aries Systems Corporation

**Availability of data and materials**<br><br>All datasets and code on which the conclusions of the paper rely must be either included in your submission or deposited in publicly available repositories (where available and ethically appropriate), referencing such data using a unique identifier in the references and in the "Availability of Data and Materials" section of your manuscript.<br><br>Have you have met the above requirement as detailed in our Minimum Standards Reporting Checklist?	Yes

Powered by Editorial Manager® and ProduXion Manager® from Aries Systems Corporation

Manuscript Click here to access/download;Manuscript;GIGA-D-20-00067-HL(2).docx

The chromosome-level draft genome of *Dalbergia odorifera*

Zhou Hong[1,†], Jiang Li[2,†], Xiaojin Liu [1], Jinmin Lian[2], Ningnan Zhang[1], Zengjiang Yang[1], Yongchao Niu[2], Zhiyi Cui[1], Daping Xu[1,*]

[1] State Key Laboratory of Tree Genetics and Breeding, Research Institute of Tropical Forestry, Chinese Academy of Forestry, Guangzhou 510520, China, [2] Biozeron Shenzhen Inc., Shenzhen 518000, China

[*]Correspondence address. Daping Xu, State Key Laboratory of Tree Genetics and Breeding, Research Institute of Tropical Forestry, Chinese Academy of Forestry, Guangzhou 510520, China. Tel: +86 020 87033626; E-mail: gzfsrd@163.com.

[†]These authors contributed equally to manuscript preparation.

ORCIDs:

Zhou Hong, 0000-0001-9955-9469

Jiang Li,0000-0003-2099-8165

Xiaojin Liu, 0000-0001-9677-6388

Jinmin Lian, 0000-0002-2063-2086

Ningnan Zhang, 0000-0001-6509-5213

Zengjiang Yang, 0000-0002-9474-8583

Yongchao Niu, 0000-0002-9774-5417

Zhiyi Cui, 0000-0002-8162-1313

Daping Xu, 0000-0003-3701-1232

Abstract

Backgroud: *Dalbergia odorifera* T. C. Chen (Fabaceae) is an IUCN red listed tree. This tree is of high medicinal and commercial value due to its officinal, insect-proof, durable heartwood. However, there is a lack of genome reference, which has hindered development of studies on the heartwood formation. Findings: We presented the first chromosome-scale genome assembly of *D. odorifera* obtained based on Illumina paired-end sequencing, Pacific Bioscience single-molecule real-time sequencing, 10× Genomics linked-reads, and Hi-C technology. We assembled 97.68% of the 653.45 Mb *D. odorifer*a genome with scaffold N50 and contig sizes of 56.16 Mb and 5.92 Mb, respectively. Ten super-scaffolds corresponding to the 10 chromosomes were assembled with the longest scaffold reaching 79.61 Mb. Repetitive elements account for 54.17% of the genome, and 30,310 protein-coding genes were predicted from the genome, of which approximately 92.6% were functionally annotated. The phylogenetic tree showed that *D. odorifera* diverged from the ancestor of *A. thaliana* and *P. trichocarpa*, and then separated from *G. max* and *C. cajan*. Conclusions: We sequence and reveal the first chromosome-level *de novo* genome of *D. odorifera*. These studies provide valuable genomic resources for the research of heartwood formation in *D. odorifera* and other timber trees. The high-quality assembled genome can also be used as reference for comparative genomics analysis and future population genetic studies of *D. odorifera*.

Key words: *Dalbergia odorifera* T. Chen; *de novo* sequencing; chromosome-level

genome assembly; annotation; phylogeny

Background

Dalbergia odorifera T. C. Chen (NCBI:txid499988), formerly named *Dalbergia hainanensis* Merr. et Chun, is a medium-sized evergreen tree belonging to the Fabaceae family. *D. odorifera* originated in Hainan, China and has been gradually introduced and cultivated in Guangdong, Fujian, Zhejiang, Guangxi, and Yunnan, China. *D. odorifera* is an ideal biological model to study the mechanism underlying high-quality heartwood (HW) formation due to its insect-proof, durable, fragrant, beautiful HW [1]. HW is defined as the central wood layers of a tree (Additional file 2: Fig. S1). This tissue, containing nonliving cells and nonfunctioning xylem tissue, can affect tree health, with broader implications for forest health [2]. The natural durability of wood as well as the biological, technological, and aesthetic parameters of wood and wood products depend on the presence, quality, and quantity of HW, which is strongly affected by external stimuli [3]. Flavonoids, which are the major compounds found in *D. odorifera*, are a main class of secondary metabolites that strongly affect various properties of HW, including durability and the color of wood products [2]. Besides, flavonoids are crucial for plant resistance against pathogenic bacteria and fungi, and flavonoid production can be induced by fungal invasion [4]. It is worth noting that carbohydrates can also affect flavonoid accumulation and the formation of phenolic extractives, which contribute to the natural durability of wood during HW formation [5]. Apart from its excellence as a wood product, the HW of *D. odorifera*, which is

known as “JiangXiang” in traditional Chinese medicine, has been included in the Chinese Pharmacopoeia for decades and is widely used to dissipate stasis, stop bleeding, and relieve pain. *D. odorifera* HW is also used to treat blood stagnation syndrome, ischemia, swelling, necrosis, and rheumatic pain in Korea [6]. Due to its great medicinal and commercial value, *D. odorifera* is becoming more and more rare: Only limited numbers of individuals are found in parts of their original habitat, with highly fragmented populations present in the remaining forests of Hainan Island [7]. It is worth noting that *D. odorifera* has been listed on the IUCN’s (International Union for Conservation of Nature) red list by the World Conservation Monitoring Centre (WCMC) since 1998[8].

Despite the commercial interest and increasing demand for *D. odorifera*, the lack of a genome sequence for this species has limited analysis of the mechanism underlying HW formation in *D. odorifera*, which has seriously hampered conservation and breeding efforts. Advances in sequencing and assembly technology have made it possible to obtain chromosome-level reference genome sequences for organisms once thought to be intractable, including forest trees, which always have high heterozygosity.

Herein, we used Illumina short reads, Pacific Bioscience’s single-molecule real-time sequencing long reads, Hi-C data, and 10× Genomics linked-reads data to assemble the first chromosome-level genome of *D. odorifera*. We revealed the genomic features of *D. odorifera*, including repeat sequences, gene annotation, and evolution. This high-quality genome would provide the fundamental genetic information to study

the durable HW formation of *D. odorifera* and related species.

Data Description

Sample collection and sequencing

An individual plant of cultivar *D. odorifera* from Guangzhou, China (113.38°E, 23.19°N) was chosen as a tissue source for sequencing. After collection healthy, fresh leaves were snap frozen in liquid nitrogen, followed by preservation at -80 °C in the laboratory prior to DNA extraction. To obtain the whole-genome sequences, genomic DNA was extracted using the cetyltrimethylammonium bromide (CTAB) method [9]. The quality and quantity of the isolated DNA were checked by electrophoresis on a 1% agarose gel and a NanoPhotometer® spectrophotometer (IMPLEN, CA, USA), and the DNA was then accurately quantified using Flurometer (Life Technologies, CA, USA).

In order to generate a chromosome-scale assembly, four different technologies were applied: Illumina's paired-end sequencing, Pacific Bioscience's single-molecule real-time sequencing, 10× Genomics link-reads, and Hi-C technology. NEB Next® Ultra DNA Library Prep Kit (NEB, USA）was used to construct Illumina's paired-end library. 0.5 ug genomic DNA molecules were fragmented, end-paired and ligated to adaptor. The ligated fragments were fractionated on agarose gels and purified by PCR amplification to produce sequencing libraries. Two paired-end (PE) libraries were constructed with insert length of 350bp according to Illumina's protocol (Illumina, San Diego, CA, USA) and 73.88 Gb (113.06 × coverage) sequencing data were produced

on the Illumina Hiseq4000 System (Illumina HiSeq 3000/HiSeq 4000 System, RRID: SCR_016386). The preparation of PacBio library was followed the protocol "Preparing *Arabidopsis* Genomic DNA for Size-Selected ~20 kb SMRTbell™ Libraries" [10]. PacBio reads were sequenced by the Sequel platform (PacBio Sequel System, RRID:SCR_017989), which gained 67.80 Gb (103.76 × coverage) sequencing data. Subreads were filtered with the default parameters. Finally, we obtained 67.74 Gb of subreads data. The average and the N50 length of long subreads reached to 11.20 kb and 17.40 kb, respectively (Additional file 1: Table S2). For 10× Genomics library preparation, purified high-molecular-weight genomic DNA of high quality was incubated with Proteinase K and RNaseA for 30 minutes at 25°C. DNA was further purified, indexed, and partitioned into bar coded libraries that were prepared using the GemCode kit (10× Genomics, Pleasanton, CA). Following the GemCode procedure, 1.0 ng of DNA was used for gel beads in emulsion (GEM) reactions in which DNA fragments were partitioned into molecular reactors to extend the DNA and to introduce specific 14-bp partition bar codes. Subsequently, GEM reactions were polymerase chain reaction (PCR)-amplified. The PCR cycling protocol was as follows: 95°C for 5 minutes; cycled 18×: 4°C for 30 seconds, 45°C for 1second, 70°C for 20 seconds, and 98°C for 30 seconds; held at 4°C. The PCR products were purified as described in the GemCode protocol. Purified DNA was sheared, end-repaired, adenylation tailed, and universal adapter ligated, and samples were indexed according to the manufacturer's recommendations. The whole genome GemCode library with an insert size of 500bp was sequenced using 150 bp PE mode sequencing on Illumina

HiSeq 4000 System (Illumina HiSeq 3000/HiSeq 4000 System, RRID: SCR_016386), which produced 118.27 Gb (180.99 × coverage) sequencing data. The whole-genome Hi-C library was constructed as described in the manufacturer’s recommendations [11] and sequenced with 150 bp PE sequencing on Illumina Hiseq 4000 System (Illumina HiSeq 3000/HiSeq 4000 System, RRID: SCR_016386), of which 156.84 Gb (240.02 × coverage) data were generated. The enzyme used in Hi-C library was DpnII and it used cuts at "GATC". All the raw sequence data generated by the Illumina platform were filtered by the following criteria: filtered reads with adapters, filtered reads with N bases more than 10%, and filtered reads with low-quality bases (≤ 5) more than 50%. All sequence data were summarized in Table 1.

To fully assist genome annotation, five tissues (flower, leaf, root, seed and stem) were collected. Total RNA from each tissue was isolated using the NEBNext Poly (A) mRNA Magnetic Isolation Module and treated with DNase I (Thermo Fisher Scientific, Wilmington, DE, USA) to remove genomic DNA. The RNA integrity of each tissue was confirmed with a Bioanalyzer 2100 (Agilent Technologies, Santa Clara, USA). For each tissue, RNA-sequencing library was prepared using the NEBNext Ultra RNA Library Prep Kit for Illumina with an insert size of 300 bp. 150 bp PE mode sequencing was performed on Illumina HiSeq 4000 System (Illumina HiSeq 3000/HiSeq 4000 System, RRID: SCR_016386). After removing reads containing adapters, reads containing poly-N, and low-quality reads from the raw data, 44.85 Gb clean data were generated (Additional file 1: Table S11).

Genome size estimation and genome assembly

The genome size of *D. odorifera* was estimated with the following formula: genome size = [Num (total k-mer) -Num (erroneous k-mer)]/mean depth of k-mer. 17-mers were counted as 55,992,417,644 from short clean reads. The total count of error k-mers was 449,157,644 and the k-mer depth was 85 (Additional file 1: Table S3; Additional file 2: Fig. S2). The genome size of *D. odorifera* was estimated to be ~653.45 Mb and the heterozygosity was approximately 0.54%. Besides, we used around 25× Illumina's paired-end sequencing clean reads to make GenomeScope (GenomeScope, RRID:SCR_017014) v1.0.0 [12] analysis. The rate of genome heterozygosity estimated by GenomeScope (GenomeScope, RRID:SCR_017014) was around 0.59% and the genome size was around 623.46 Mb (Additional file 2: Fig. S3).

Falcon (Falcon, RRID:SCR_016089) v2.0.5 [13] assembler was used to assemble the contig of the *D. odorifera* genome, with parameters as follows: --max_diff 100 --max_cov 100 --min_cov 2 --min_len 5000. After Falcon assembly, the genome was polished by Quiver v5.0 [14]. Illumina short reads were used to produce a more contiguous genome with fewer errors with Pilon (Pilon, RRID:SCR_014731) v1.18 [15]. Initial assembly of the PacBio data alone resulted in a contig N50 of 6.19Mb. The PacBio contigs were first scaffolded based on 10× Genomics linked-read data using fragScaff v140324 [16] software, and the resulting scaffolds were further connected to super-scaffolds by Hi-C technology using the methods described by Bickhart et al.[17]. According to the Hi-C clustering results, the genome sequences were divided into 10 chromosome clusters (Fig.1; Additional file 1:

Table S7; Additional file 2: Fig. S6). Each technology greatly improved the assembly quality (Additional file 1: Table S9). All these processes yielded a final draft *D. odorifera* genome assembly with a total length of 638.26 Mb, scaffold N50 of 56.16 Mb, and longest scaffold of 79.61 Mb (Table 2). The N50 of this assembly is almost the best in the Fabaceae family that have been recently completed (Additional file 2: Fig. S7).

To assess the quality of the genome assembly, we mapped paired-end reads with short insert sizes onto the assembly using BWA (BWA, RRID:SCR_010910) v 0.7.17-r1188 [18] with mem algorithm. Nearly 98% of these reads could be mapped to the *D. odorifera* draft genome, with the genome coverage reaching 99.63% (Additional file 1: Table S4). In addition, of the 1,440 genes identified using Embryophyta Benchmarking Universal Single-Copy Orthologs (BUSCO, RRID:SCR_015008 v3.0 [19]), 92.2% complete and 1.7% partial genes were identified in the assembled genome (Additional file 1: Table S6), and 235 of the 248 genes identified using Core Eukaryotic Genes Mapping Approach (CEGMA v2.5 [20]) were retrieved in the assembly (Additional file 1: Table S5). These results indicate that the newly generated *D. odorifera* genome is of high quality and we successfully assembled the major genic regions of this precious plant species.

Repeat sequences in the *D. odorifera* genome assembly

Homology searching and *ab initio* prediction were applied to study the repetitive sequences in the *D. odorifera* genome. For homology-based prediction, we used

RepeatMasker (RepeatMasker, RRID:SCR_012954) v4.0.7 [21] and RepeatProteinMask v4.0.7 to search against Repbase [22]. For *ab initio* prediction, we used Tandem Repeats Finder v4.07b [23], LTR_FINDER (LTR_Finder, RRID:SCR_015247) v 1.07 [24], RepeatModeler (RepeatModeler, RRID:SCR_015027) v1.0.8, Piler v1.0 [25], and RepeatScout (RepeatScout, RRID:SCR_014653) v1.0.5 [26] with default parameters. Overall, the identified repeat sequences in the *D. odorifera* genome accounted for 54.17% and total length of those accounted for 345.73 Mb (Additional file 1: Table S10). Long terminal repeats (LTRs) were the most abundant (comprising 37.7% of the genome), followed by DNA transposons (9.16% of the genome; Table 3).

Protein coding gene prediction and ncRNA prediction

Three approaches were employed to predict the protein-coding genes in the *D. odorifera* genome, including homologous comparison, *ab initio* prediction, and RNA-seq-based annotation. For homologous comparison, the reference protein sequences from the Ensembl database and NCBI database for seven species, including *Arabidopsis thaliana*, *Populus trichocarpa*, *Eucalyptus grandis*, *Medicago truncatula*, *Arachis duranensis*, *Malus domestica*, and *Glycine max*（Additional file 1: Table S1） were aligned against the *D. odorifera* genome using TBLASTN (TBLASTN, RRID:SCR_011822) v2.2.15 [27] search with E-value 1e-5 in the "-F F" option. All TBLAST hits were concatenated after filtering low-quality records. The sequence of each candidate gene was further extended upstream and downstream by 1,000 bp to

represent the entire gene region. Gene structures were predicted using GeneWise (GeneWise, RRID:SCR_015054) v2.4.1 [28]. Genes predicted in a homology-based manner were viewed as the “Homology-set”. RNA-sequencing (RNA-seq) data derived from 5 tissues (Additional file 1: Table S11) were assembled using Trinity (Trinity, RRID:SCR_013048) v2.0 [29]. The assembled sequences (Additional file 1: Table S12) were aligned against the *D. odorifera* genome using Program to Assemble Spliced Alignment (PASA, RRID:SCR_014656 v2.3.3 [30]), which assembles effective alignments into gene structures [31]. Gene models created by PASA were viewed as the PASA-T-set (PASA Trinity set). At the same time, RNA-seq reads were aligned to the *D. odorifera* genome using TopHat (TopHat, RRID:SCR_013035) v2.0.9 [32] with default parameters, then the mapped reads were assembled into gene models with Cufflinks (Cufflinks, RRID:SCR_014597) v2.2.1[33] (Cufflinks-set). We simultaneously employed five tools for *ab initio* prediction, including Augustus (Augustus, RRID:SCR_008417) v3.2.3 [34], GeneID (GeneID, RRID:SCR_002473) v1.4.4 [35], GeneScan (GENSCAN, RRID:SCR_012902) v1.0 [36], GlimmerHMM (GlimmerHMM , RRID:SCR_002654) v3.52 [37], and SNAP (SNAP, RRID:SCR_007936) v2006-07-28 [38]. Notably, the parameters were computationally optimized by training a set of high-quality protein sequences derived from the PASA gene models. Finally, the non-redundant reference gene set was generated using EVidenceModeler (EVidenceModeler, RRID:SCR_014659) v1.1.1. Weights for each type of evidence were as follows: PASA-T-set > Homology-set > Cufflinks set > Augustus > GeneID = SNAP = GlimmerHMM = GeneScan. The gene

models were further updated by PASA to identify untranslated regions and to obtain information about alternative splicing variation. Using this strategy, we annotated 30,311 protein-coding genes, with an average coding sequence length of 1.12 kb and an average of 4.93 exons per gene (Additional file 1: Table S13). Nearly 96% of the genes were supported by homology searches and/or the presence of expressed transcripts (Additional file 2: Fig. S9). The gene components were similar to those of related species (Additional file 2:Fig. S10).

Four types of noncoding RNAs were annotated using tRNAscan-SE (tRNAscan-SE, RRID:SCR_010835) v1.23 and the Rfam (Rfam, RRID:SCR_007891) database v9.1 [39]. We found 112 ribosomal RNA (rRNA), 582 transfer RNA (tRNA), 747 microRNAs (miRNA), and 473 snRNA genes in the *D. odorifera* genome (Additional file 1: Table S15).

Functional annotation of protein-coding genes

Firstly, gene functions were assigned to the translated protein-coding genes using BLASTP (BLASTP, RRID:SCR_001010) v2.2.15 [40] based on their highest match to proteins in the Swiss-Prot (Swiss-Prot, RRID:SCR_002380) [41] , KEGG (KEGG, RRID:SCR_012773) [42], and NR databases. The BLASTP e-value cutoff was set as 1E-5. Secondly, motifs and domains in the protein-coding genes were retrieved by performing InterproScan (InterproScan, RRID:SCR_005829) v4.7 [43] searches against six protein databases: ProDom, PRINTS, Pfam, SMART, PANTHER, and PROSITE. GO (GO, RRID:SCR_002811) [44] terms for each gene were detected from

the corresponding InterPro entries. In total, 92.6% (28,069 genes) were successfully annotated at least one function terms (Additional file 1: Table S14).

Phylogenetic analysis and species divergence time estimation

To investigate the phylogenic positions and to further dissect the molecular underpinnings of *D. odorifera*, we retrieved nucleotide and protein data for nine plant species from the NCBI and Ensembl databases, including *Arachis duranensis*, *Arabidopsis thaliana*, *Cajanus cajan, Eucalyptus grandis, Glycine max, Malus domestica*, *Medicago truncatula, Populus trichocarpa,* and *Vitis vinifera* （Additional file 1: Table S1）. To remove redundancy caused by alternative splicing variations, we retained only gene models at each gene locus that encoded the longest protein sequence. To exclude putative fragmented genes, genes encoding protein sequences shorter than 30 amino acids were filtered out. The OrthoMCL (Ortholog Groups of Protein Sequences, RRID:SCR_007839) v2.0 [45] software was used to cluster genes into gene families with the parameter "-inflation 1.5". A total of 27,195 gene families were constructed, 9,108 of which were common among species (Additional file 2: Fig. S11). In addition, we identified 12,092 gene families shared between five Fabaceae species while 577 gene families that were unique to *D. odorifera* (Fig. 2A; Fig. 2B). The 1,211 species-specific genes in the unique families were significantly overrepresented in the categories of regulation of replication and repair, such as mismatch repair, DNA replication, nucleotide-excision repair, and homologous recombination (Additional file 2: Fig. S12).

Protein sequences from 390 single-copy gene families were used for phylogenetic tree reconstruction. MUSCLE (MUSCLE, RRID:SCR_011812) v3.8.31 [46] was employed to generate multiple sequence alignments for protein sequences in each single-copy family with default parameters. The alignments of each family were concatenated into a super alignment matrix to reconstruct the phylogenetic tree via the maximum likelihood method using RAxML v8.2.12 [47] (RAxML, RRID:SCR_006086). Divergence time between species was estimated using MCMCtree in PAML (PAML, RRID:SCR_014932) v1.3.1 [48]. The parameters used for MCMCtree analysis as follow: burn-in=10000, sample-number=100000, sample-frequency=2. Divergence time for *A. thaliana* and *P. trichocarpa*, *G. max* and *C. cajan*, *G. max* and *M. truncatula*, *G. max* and *M. domestica*, *A. thaliana*, and *V. vinifera* from the TimeTree database [49] was used as the calibration point. The results indicated that the split between *D. odorifera* and *A. duranensis* occurred approximately 40.3 million years ago (Additional file 2: Fig. S14).

Gene family expansion and contraction

We identified expanded and contracted gene families using CAFÉ(CAFÉ, RRID:SCR_005983) v3.1 [50], which employs a random birth and death model to study gains and losses in gene families across a user-specified phylogeny. There were 41 and 214 gene families that appear to have expanded and contracted, respectively (Fig. 2C). The expanded gene families were significantly clustered in 15 KEGG

pathways, including replication and repair, environmental adaptation, signal transduction, secondary metabolites, and carbohydrate metabolism (Additional file 2: Fig. S13).

Whole-genome duplication analysis

Whole genome duplication (WGD) analysis was performed by searching for collinearity with the *D. odorifera* genome using MCScan (MCScan, RRID:SCR_017650) v0.8 software [51]. Repeated gene pairs located in internal collinear segments were processed for sequence alignment analysis. The 4-fold degenerate transversion rate (4DTV) values were calculated and used to construct a frequency distribution map for all repeated gene pairs. Besides, MCscan v0.8 was also employed to examine collinearity between *D. odorifera* and *P. trichocarpa*, *D. odorifera* and *A. thaliana*, *D. odorifera* and *C. cajan*, and *D. odorifera* and *G. max*. The 4DTV values of orthologous gene pairs in the collinear segment were calculated and used to construct a frequency distribution map. The 4DTV plot indicated that after the ancient so-called γ WGD event shared by core eudicots [52], *D. odorifera* had undergone a new round of WGD (Fig. 2D). The approximate time of WGD was ~66.12 Mya and ~146.93 Mya respectively.

Conclusion

In this study, we presented the genome of *D. odorifera* firstly and described its genetic attributes. The final chromosome-level genome is almost the most consecutive to date

in the Fabaceae family. This high-quality genome could be another model reference sequence for researching the protection and rational utilization of forest. Furthermore, the genome makes it possible to design re-sequencing studies to discuss the impact of genetic structure on important economic traits.

Availability of Supporting Data and Materials

Supporting data and materials are available in the *GigaScience* GigaDB database[53], with the raw DNA sequencing data deposited in the NCBI SRA database under the BioProject accession number PRJNA613774 and BioSample accession number SAMN14419398. Raw RNA sequencing data can be found via the BioProject accession number PRJNA552194.

Figures and Tables

Fig. 1: Circos plot shows the characterization of the *D.odorifera* genome. I: Syntenic regions within *D.odorifera* assembly base on homology searches were carried out by conducting with MCscan[51] requiring at least thirty genes per block (Links); II: GC content in non-overlapping 1Mb windows (Histograms); III: Percent coverage of TEs in non-overlapping 1Mb windows (Heat maps); IV: Gene density calculated on the basis of the number of genes in non-overlapping 1Mb windows (Heat maps); V: The length of super-Scaffolds in the size of Mb.

Fig. 2: Evolution of the *D. odorifera* genome. (A) Venn diagram of shared and unique orthologous gene families in *D. odorifera* and four other legumes. (B) Predicted

orthologous protein compositions for the 10 genomes. (C) Expansion and contraction of gene families. The numbers in green indicate the number of gene families that expanded in the species during evolution, and the numbers in red indicate the number of gene families that contracted. (D) 4DTV (4-fold degenerate transversion rate) plot.

Table 1: Sequencing data used for the *D. odorifera* genome assembly.

Table 2: Statistics for the *D. odorifera* genome.

Table 3: Classifications of Transposable elements (TEs) predicted by each method.

Additional files

Additional file 1: A Word file with Table S1-S15.

Additional file 2: A Word file with Fig. S1-S14.

Abbreviations

NCBI: National Center for Biotechnology Information; HW: heartwood; IUCN: International Union for Conservation of Nature; SMRT: Single-molecule real-time; PacBio: Pacific Biosciences; CTAB: cetyltrimethylammonium bromide; GEM: Gel Bead in Emulsion ; BUSCO: Benchmarking Universal Single-Copy Orthologs; CEGMA: Core Eukaryotic Genes Mapping Approach; LTRs: Long terminal repeats; PASA: Program to Assemble Spliced Alignment; GO: Gene Ontology; LTRs：Long terminal repeats; RAxML: Randomized Axelerated Maximum Likelihood; CAFÉ: Computational Analysis of Gene Family Evolution; 4DTV: 4-fold degenerate transversion rate; WGD: whole-genome duplication; Mya : million years ago;

SRA:Sequence Read Archive

Competing interests

The authors declare they have no competing financial interests.

Funding

This work was supported by the Fundamental Research Funds for the Central Non-profit Research Institution of Chinese Academy of Forestry (CAFYBB2017ZX001-4), the Fundamental Research Funds for the Central Non-profit Research Institution of Chinese Academy of Forestry (CAFYBB2016QB009), the Fundamental Research Funds for the Central Non-profit Research Institution of Chinese Academy of Forestry (CAFYBB2017SY021), National Natural Science Foundation of China (31500537) and the National Key Research and Development Program of China (2016YFD0600601).

Author contributions

Z.H. designed the sequencing strategy. X.L., N.Z., Z.Y., and Z.C. prepared and analysed the samples. Y.C.N, J.M.L., and J.L. draw the figures. J.L. and Z.H. wrote the manuscript with input from other co-authors. D.P.X. was responsible for project administration.

Acknowledgements

Thanks to reviewers for their helpful comments and constructive suggestions.

References

1. Sun S, Zeng X, Zhang D, Guo S: **Diverse fungi associated with partial irregular heartwood of Dalbergia odorifera**. *Scientific Reports* 2015, **5**:8464.
2. Celedon J, Bohlmann J: **An extended model of heartwood secondary metabolism informed by functional genomics**. *Tree physiology* 2017, **38**:1-9.
3. Kampe A, Magel E: **New Insights into Heartwood and Heartwood Formation**. *Cellular Aspects of Wood Formation*.2013.
4. Mierziak J, Kostyn K, Kulma A: **Flavonoids as Important Molecules of Plant Interactions with the Environment**. *Molecules (Basel, Switzerland)* 2014, **19**:16240-16265.
5. Park C, Kim Y, Li X, Kim H-H, Arasu M, Al-Dhabi N, Lee S-Y, Park SU: **Influence of Different Carbohydrates on Flavonoid Accumulation in Hairy Root Cultures of Scutellaria baicalensis**. *Natural product communications* 2016, **11**:799-802.
6. Kang T-H, Tian Y-H, Kim Y-C: **Isoliquiritigenin : A Competitive Tyrosinase Inhibitor from the Heartwood of Dalbergia odorifera**. *Biomolecules and Therapeutics* 2005, **13**.
7. Liu F, Hong Z, Jia H, Zhang N, Liu X, Yang Z, Lu M: **Genetic Diversity of the Endangered Dalbergia odorifera Revealed by SSR Markers**. *Forests* 2019, **10**:18.
8. **WCMC**. http://www.iucnredlist.org/details/32398/0
9. Porebski S, Bailey LG, Baum BR: **Modification of a CTAB DNA extraction protocol for plants containing high polysaccharide and polyphenol components**. *Plant Molecular Biology Reporter* 1997, **15**(1):8-15.
10. **Preparing Arabidopsis Genomic DNA for Size-Selected ~20 kb**

SMRTbellTM Libraries. http://www.pacb.com/wp-content/uploads/2015/09/Shared-Protocol-Preparing-Arabidopsis-DNA-for-20-kb-SMRTbell-Libraries.pdf

11. Xu C-Q, Hui L, Zhou S-S, Zhang D-X, Zhao W, Wang S, Chen F, Sun Y-Q, Nie S, Jia K-H *et al*: **Genome sequence of Malania oleifera, a tree with great value for nervonic acid production**. *GigaScience* 2019, **8**(2):1-14.
12. Vurture GW, Sedlazeck FJ, Nattestad M, Underwood CJ, Fang H, Gurtowski J, Schatz MC: **GenomeScope: fast reference-free genome profiling from short reads**. *Bioinformatics (Oxford, England)* 2017, **33**(14):2202-2204.
13. Pendleton M, Sebra R, Pang A, Ummat A, Franzén O, Rausch T, Stütz A, Stedman W, Anantharaman T, Hastie A *et al*: **Assembly and diploid architecture of an individual human genome via single-molecule technologies**. *Nature Methods* 2015, **12**.
14. Chin C-S, Alexander D, Marks P, Klammer A, Drake J, Heiner C, Clum A, Copeland A, Huddleston J, Eichler E *et al*: **Nonhybrid, finished microbial genome assemblies from long-read SMRT sequencing data**. *Nature methods* 2013, **10**.
15. Walker BJ, Abeel T, Shea T, Priest M, Abouelliel A, Sakthikumar S, Cuomo CA, Zeng Q, Wortman J, Young SK *et al*: **Pilon: an integrated tool for comprehensive microbial variant detection and genome assembly improvement**. *PLoS One* 2014, **9**(11):e112963.
16. Mostovoy Y, Levy-Sakin M, Lam J, Lam E, Hastie A, Marks P, Lee J, Chu C, Lin C, Dzakula Z *et al*: **A Hybrid Approach for de novo Human Genome Sequence Assembly and Phasing**. *Nature methods* 2016, **13**.
17. Bickhart DM, Rosen BD, Koren S, Sayre BL, Hastie AR, Chan S, Lee J, Lam ET, Liachko I, Sullivan ST *et al*: **Single-molecule sequencing and chromatin conformation capture enable de novo reference assembly of the domestic goat genome**. *Nature Genetics* 2017, **49**(4):643-650.
18. Li H: **Fast and Accurate Long-Read Alignment with Burrows-Wheeler Transform**. *Bioinformatics (Oxford, England)* 2010, **26**:589-595.
19. Simão F, Waterhouse R, Ioannidis P, Zdobnov E: **BUSCO: Assessing**

genome assembly and annotation completeness with single-copy orthologs. *Bioinformatics (Oxford, England)* 2015, **31**.
20. Parra G, Bradnam K, Korf I: **CEGMA: A pipeline to accurately annotate core genes in eukaryotic genomes**. *Bioinformatics (Oxford, England)* 2007, **23**:1061-1067.
21. Bergman CM, Quesneville H: **Discovering and detecting transposable elements in genome sequences**. *Briefings in Bioinformatics* 2007, **8**(6):382-392.
22. Bao W, Kojima K, Kohany O: **Repbase Update, a database of repetitive elements in eukaryotic genomes**. *Mobile DNA* 2015, **6**.
23. Benson G: **Tandem repeats finder: a program to analyze DNA sequences**. *Nucleic acids research* 1999, **27**(2):573-580.
24. Xu Z, Wang H: **LTR-FINDER: An efficient tool for the prediction of full-length LTR retrotransposons**. *Nucleic acids research* 2007, **35**:W265-268.
25. Edgar R, Myers E: **PILER: Identification and classification of genomic repeats**. *Bioinformatics (Oxford, England)* 2005, **21 Suppl 1**:i152-158.
26. Price A, Jones N, Pevzner P: **Price, A.L., Jones, N.C. & Pevzner, P.A. De novo identification of repeat families in large genomes. Bioinformatics 21, i351-i358**. *Bioinformatics (Oxford, England)* 2005, **21 Suppl 1**:i351-358.
27. Gertz E, Yu Y-K, Agarwala R, Schaffer A, Altschul S: **Composition-based statistics and translated nucleotide searches: Improving the TBLASTN module of BLAST**. *BMC biology* 2006, **4**:41.
28. Birney E, Clamp M, Durbin R: **GeneWise and Genomewise**. *Genome Res* 2004, **14**(5):988-995.
29. Grabherr M, Haas B, Yassour M, Levin J, Thompson D, Amit I, Adiconis X, Fan L, Raychowdhury R, Zeng Q *et al*: **Trinity: reconstructing a full-length transcriptome without a genome from RNA-Seq data**. *Nature biotechnology* 2013, **29**:644.
30. Haas BJ, Delcher AL, Mount SM, Wortman JR, Smith RK, Jr., Hannick LI, Maiti R, Ronning CM, Rusch DB, Town CD *et al*: **Improving the Arabidopsis genome annotation using maximal transcript alignment**

assemblies. *Nucleic acids research* 2003, **31**(19):5654-5666.

31. Haas B, Salzberg S, Zhu W, Pertea M, Allen J, Orvis J, White O, Buell C, Wortman J: **Automated eukaryotic gene structure annotation using EVidenceModeler and the Program to Assemble Spliced Alignments**. *Genome biology* 2008, **9**:R7.
32. Kim D: **TopHat2: accurate alignment of transcriptomes in the presence of insertions, deletions and gene fusions**. *Genome Biol* 2013, **14**:R36.
33. Trapnell C, Roberts A, Goff L, Pertea G, Kim D, Kelley D, Pimentel H, Salzberg S, Rinn J, Pachter L: **Differential gene and transcript expression analysis of RNA-Seq experiments with TopHat and Cufflinks**. *Nature protocols* 2012, **7**:562-578.
34. Stanke M, Morgenstern B: **AUGUSTUS: A web server for gene prediction in eukaryotes that allows user-defined constraints**. *Nucleic acids research* 2005, **33**:W465-467.
35. Guigó R, Knudsen S, Drake N, Smith T: **Prediction of gene structure**. *Journal of molecular biology* 1992, **226**:141-157.
36. Aggarwal G, Ramaswamy R: **Ab initio gene identification: Prokaryote genome annotation with GeneScan and GLIMMER**. *Journal of Biosciences* 2002, **27**(1):7-14.
37. Majoros W, Pertea M, Salzberg S: **TigrScan and GlimmerHMM: Two open source ab initio eukaryotic gene-finders**. *Bioinformatics (Oxford, England)* 2004, **20**:2878-2879.
38. Korf I: **Gene finding in novel genomes**. *BMC Bioinformatics* 2004, **5**(1):59.
39. Griffiths-Jones S, Moxon S, Marshall M, Khanna A, Eddy S, Bateman A: **Rfam: Annotating Non-Coding RNAs in Complete Genomes**. *Nucleic acids research* 2005, **33**:D121-124.
40. Altschul S: **Basic local alignment search tool (BLAST)**. *Journal of Molecular Biology* 1990, **215**:403-410.
41. Bairoch A, Apweiler R: **The SWISS-PROT protein sequence data bank and its supplement TrEMBL in 1998**. *Nucleic Acids Research* 1997, **25**:31-36.
42. Ogata H, Goto S, Sato K, Fujibuchi W, Bono H, Kanehisa M: **KEGG:**

kyoto Encyclopedia of Genes and Genomes. *Nucleic acids research* 1999, **27**:29-34.

43. Jones P, Binns D, Chang H-Y, Fraser M, Li W, McAnulla C, McWilliam H, Maslen J, Mitchell A, Nuka G *et al*: **InterProScan 5: Genome-scale Protein Function Classification**. *Bioinformatics (Oxford, England)* 2014, **30**.

44. Ashburner M, Ball C, Blake J, Botstein D, Butler H, Cherry J, Davis AP, Dolinski K, Dwight S, Eppig J *et al*: **Gene ontology: tool for the unification of biology. The Gene Ontology Consortium**. *Nature genetics* 2000, **25**:25-29.

45. Li L, Stoeckert C, Roos D: **OrthoMCL: Identification of Ortholog Groups for Eukaryotic Genomes**. *Genome research* 2003, **13**:2178-2189.

46. Edgar R: **MUSCLE: Multiple Sequence Alignment with High Accuracy and High Throughput**. *Nucleic acids research* 2004, **32**:1792-1797.

47. Stamatakis A: **RAxML Version 8: A Tool for Phylogenetic Analysis and Post-Analysis of Large Phylogenies**. *Bioinformatics (Oxford, England)* 2014, **30**.

48. Yang Z: **PAML 4: Phylogenetic Analysis by Maximum Likelihood**. *Molecular Biology and Evolution* 2007, **24**(8):1586-1591.

49. **Timetree.** http://www.timetree.org/

50. Hahn M, Demuth J, Han S-G: **Accelerated Rate of Gene Gain and Loss in Primates**. *Genetics* 2007, **177**:1941-1949.

51. Tang H, Bowers J, Wang X, Ming R, Alam M, Paterson A: **Synteny and Collinearity in Plant Genomes**. *Science (New York, NY)* 2008, **320**:486-488.

52. Bowers J, Chapman B, Rong J, Paterson A: Bowers JE, Chapman BA, Rong JK, Paterson AH. **Unravelling angiosperm genome evolution by phylogenetic analysis of chromosomal duplication events.** *Nature* 2003, **422**:433-438.

53. Hong Z; Li J; Liu X; Lian J; Zhang N; Yang Z; Niu Y; Cui Z; Xu D. **Supporting data for "The chromosome-level draft genome of Dalbergia odorifera"** *GigaScience* Database 2020. http://dx.doi.org/10.5524/100760

558

Table 1: Sequencing data used for the *D. odorifera* genome assembly

Libraries	Insert size (bp)	Raw data (Gb)	Clean data (Gb)	Read length (bp)	Sequence coverage (X)
Illumina reads	350 bp	73.88	73.80	150	113.06
PacBio reads	20 Kb	67.80	67.74	11,201	103.76
10X Genomics	500 bp	118.27	116.06	150	180.99
Hi-C	350bp	156.84	155.86	150	240.02
Total	-	416.79	413.46	-	637.83

Note: The coverage was calculated using an estimated genome size of 653.45 Mb.

Table2 Statistics for the *D. odorifera* genome

Assembly feature	Value
Estimated genome size (by *k*-mer analysis)	653.45 Mb
Number of scaffolds	384
Contig N50	5.92 Mb
Scaffold N50	56.16 Mb
Longest Scaffold	79.61 Mb
Assembly length	638.26 Mb
Assembly % of genome	97.68
Repeat region % of assembly	54.17
Predicted gene models	30,310
Average coding sequence length	1,121.36 bp
Average exons per gene	4.93

Click here to access/download;Table;Table 3.xls

Table3 Classifications of Transposable elements (TEs) predicted by each method

Type	Repbase+Denovo		TE proteins		Combined TEs	
	Length (Bp)	% in genome	Length (Bp)	% in genome	Length (Bp)	% in genome
DNA	55,376,910	8.68	10,310,575	1.62	58,455,913	9.16
LINE	6,620,833	1.04	4,004,118	0.63	9,241,306	1.45
SINE	292,685	0.05	0	0	292,685	0.05
LTR	236,380,844	37.03	56,821,684	8.9	240,620,255	37.7
Other	0	0	0	0	0	0
Satellite	116,222	0.02	0	0	116,222	0.02
Simple_repeat	1,238,189	0.19	0	0	1,238,189	0.19
Unknown	48,638,508	7.62	0	0	48,638,508	7.62
Total	332,705,455	52.13	71,048,270	11.13	340,525,618	53.35

Note: Repbase+Denovo: RepeatMasker results based on Repbase, RepeatModeler, RepeatScout, Piler, and LTR_FINDER; TE proteins: RepeatProteinMask results based on Repbase; Combined TEs: combined results of Denovo+Repbase and TE proteins.

Circos plot shows the characterization of the D.odorifera genome

Click here to access/download;Figure;Fig. 1.png

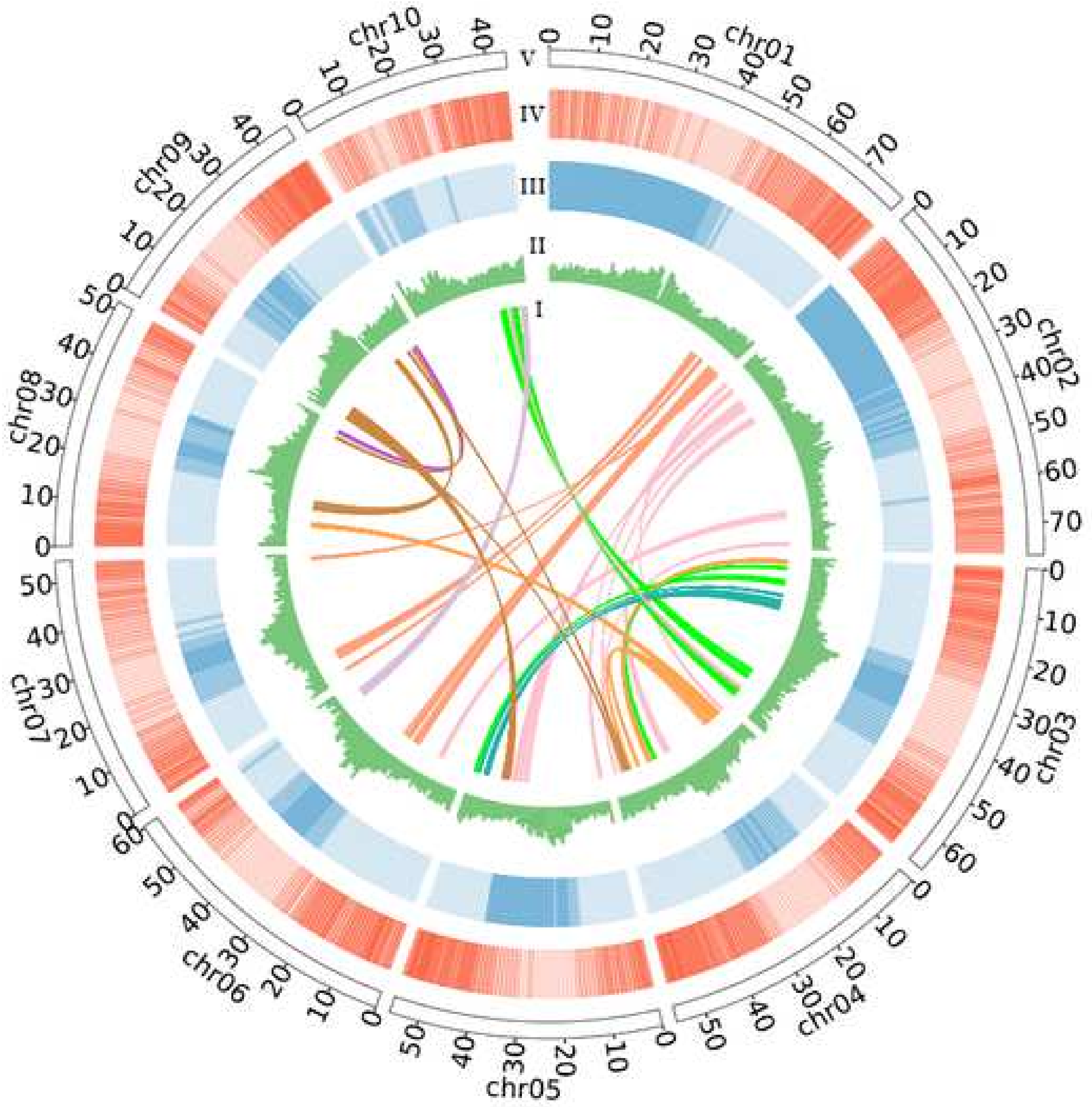

Evolution of the D. odorifera genome.

Click here to access/download;Figure;Fig. 2.jpg

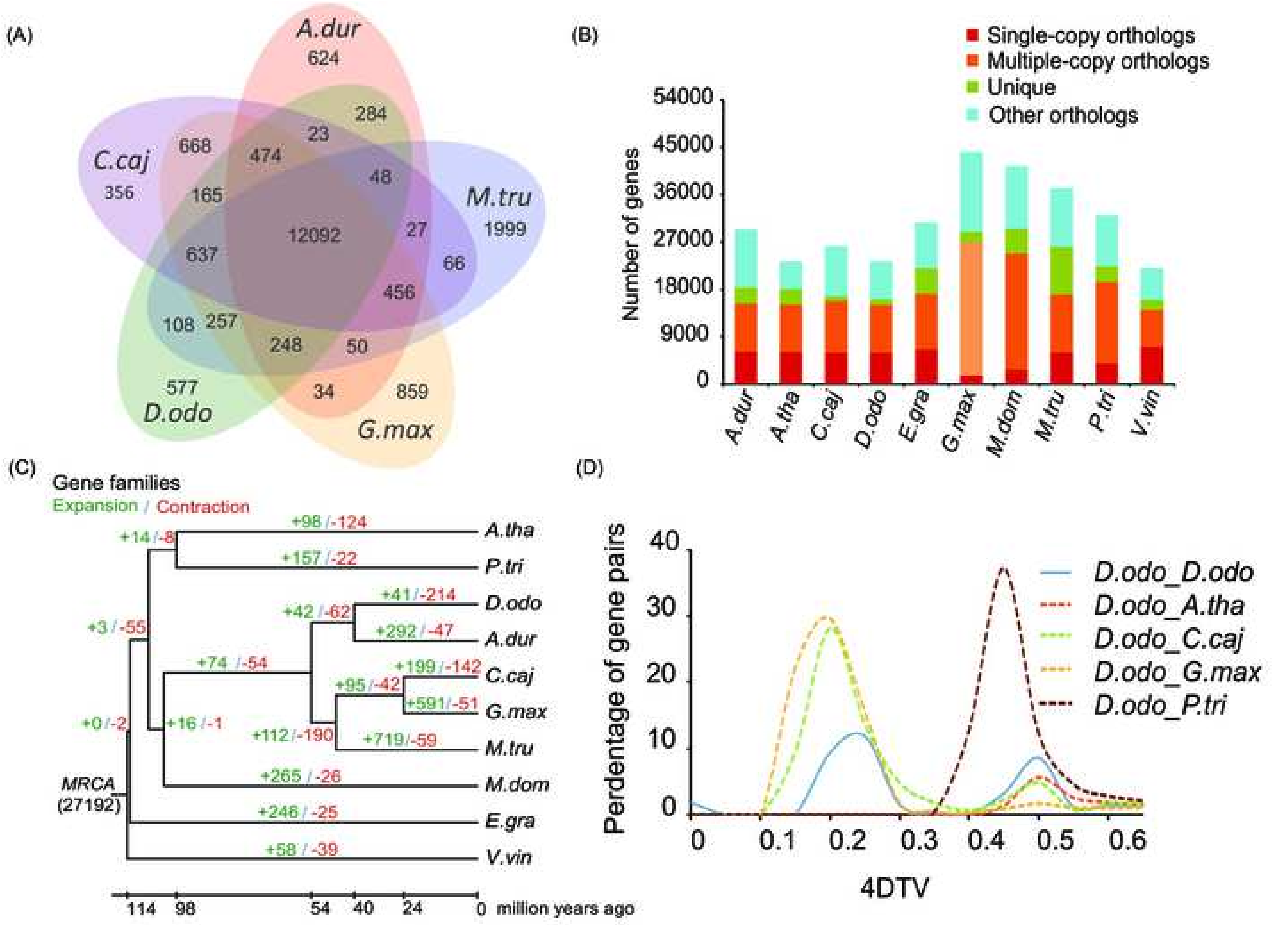

Figure

Click here to access/download;Figure;collinearity figure.pdf

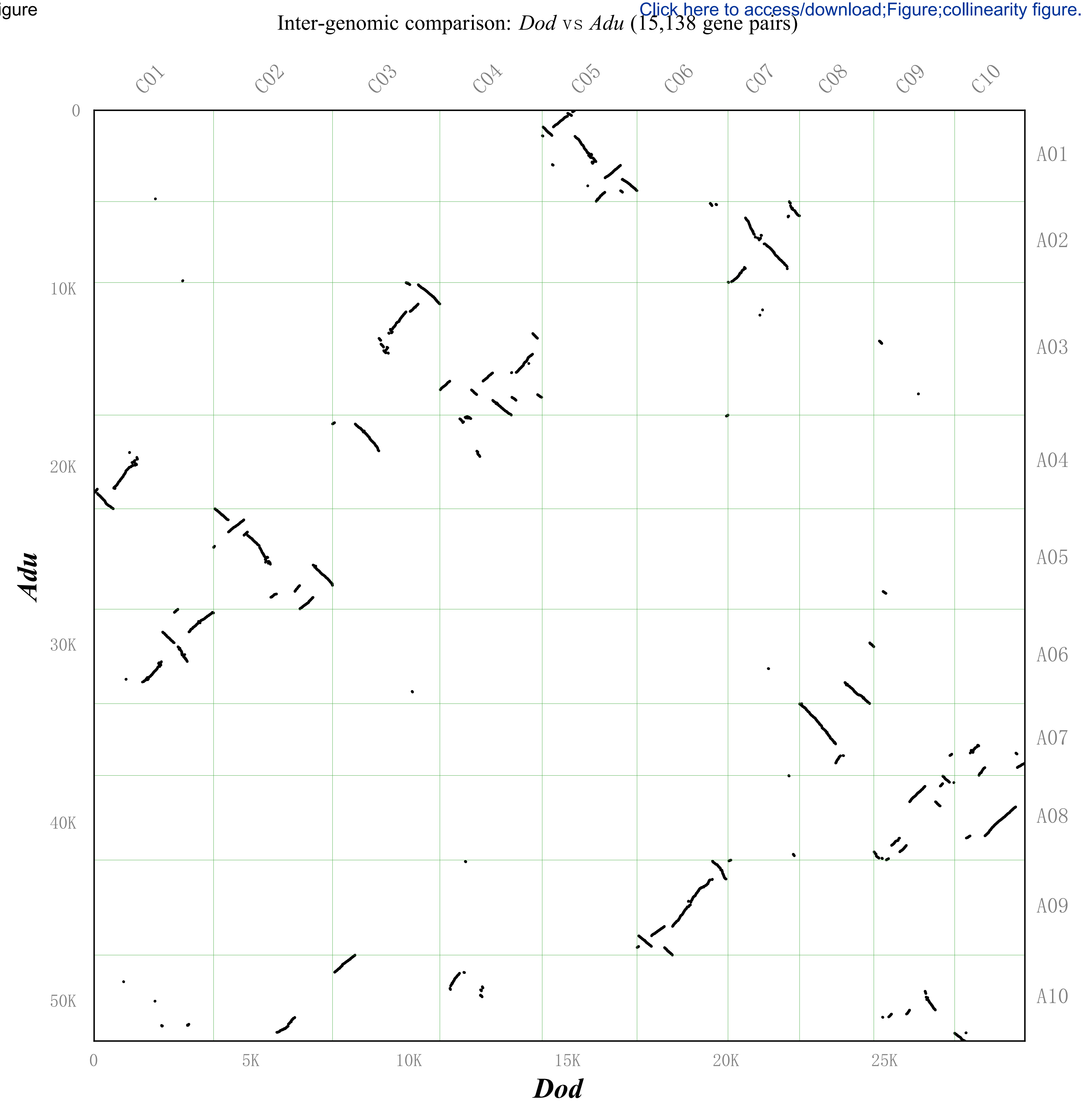

Click here to access/download
Supplementary Material
Additional file 1.docx

Click here to access/download
Supplementary Material
Additional file 2.docx

 Click here to access/download;Personal Cover;Response to Reviewers GIGA-D-20-00067.docx

Dear Dr Xu,

Your manuscript "Chromosome-level genome of the Dalbergia odorifera provide insight into the antimicrobial properties of its heartwood" (GIGA-D-20-00067) has been assessed by our reviewers. Although it is of interest, we are unable to consider it for publication in its current form. The reviewers have raised a number of points which we believe would improve the manuscript and may allow a revised version to be published in GigaScience.

For a Data Note in-depth analysis is not required, but we do require some validation. Comparative genomics is a good way to do this and is usually recommended, but you do not necessarily need to do any biological experimentation to meet this requirement. Answers: Thanks for your reminding. The collinearity analyses have been done using jcvi method (https://github.com/tanghaibao/jcvi). Firstly, we find out all the published genome of legumes on the website: https://www.plabipd.de/plant_genomes_pa.ep. According to the divergence time from the website: http://www.timetree.org/, *Arachis duranensis* was the closest legume specie to *Dalbergia odorifera*. So we make a collinearity analysis of *Arachis duranensis* between *Dalbergia odorifera*. While due to the long genetic distance (~40 MYA) and low genome collinearity between *Arachis duranensis* and *Dalbergia odorifera*, it is not suitable for verification of *Dalbergia odorifera* assembly by comparative genomics so far.

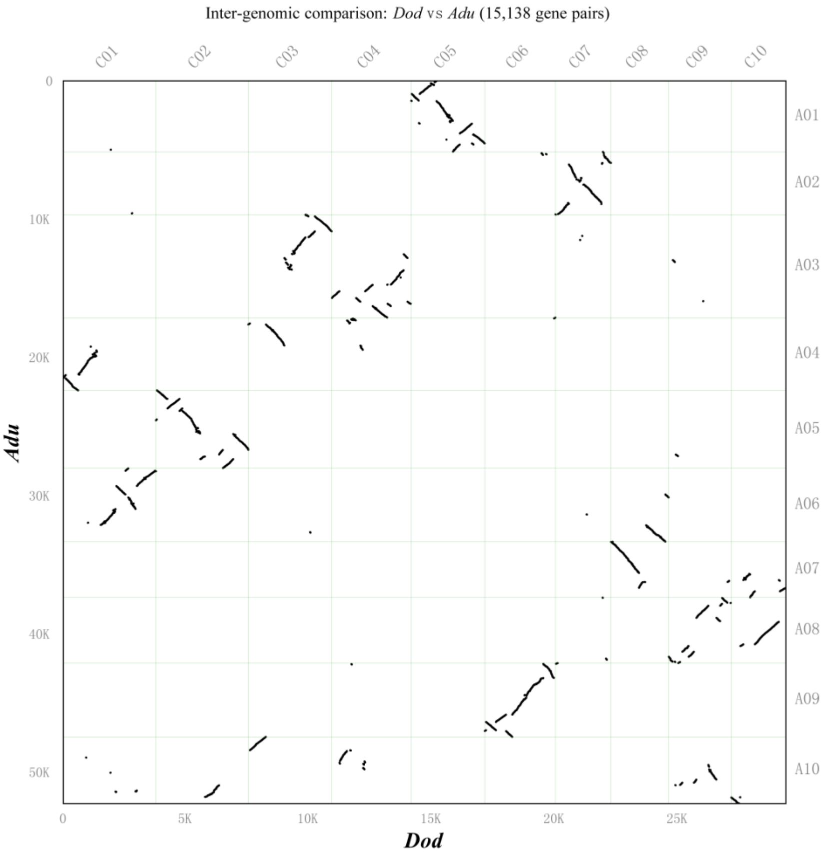

Their reports, together with any other comments, are below. Please also take a moment to check our website at https://www.editorialmanager.com/giga/ for any additional comments that were saved as attachments.

In addition, please register any new software application in the bio.tools and SciCrunch.org databases to receive RRID (Research Resource Identification Initiative ID) and biotoolsID identifiers, and include these in your manuscript. This will facilitate tracking, reproducibility and re-use of your tool.
Answers: Thanks for your reminding. The RRID have been added.

If you are able to fully address these points, we would encourage you to submit a revised manuscript to GigaScience. Once you have made the necessary corrections, please submit online at:
https://www.editorialmanager.com/giga/
If you have forgotten your username or password please use the "Send Login Details" link to get your login information. For security reasons, your password will be reset.

Please include a point-by-point within the 'Response to Reviewers' box in the submission system. Please ensure you describe additional experiments that were

carried out and include a detailed rebuttal of any criticisms or requested revisions that you disagreed with. Please also ensure that your revised manuscript conforms to the journal style, which can be found in the Instructions for Authors on the journal homepage. If the data and code has been modified in the revision process please be sure to update the public versions of this too.

The due date for submitting the revised version of your article is 20 Jul 2020.

I look forward to receiving your revised manuscript soon.

Reviewer #1: This article is presenting the genome assembly of Dalbergia odorifera which is a tree of interest for its commercial and medicinal properties. The genome assembly is at the chromosome scale and has been well annotated. The phylogenetic and metabolite analysis produced very interesting results. But some methods had to be better described as listed below :

Line 79. The DNA extraction was performed using the CTAB method. There is no reference for this method. Please add a reference or explain the method.

Answers: Thanks for your reminding. The reference has been added.

Line 97. The genome size was estimated by k-mer distribution analysis. Please clarify which method you used ? How did you estimate the heterozygosity rate ? Did you compare your results with results from Genomescope for example ?

Answers: The genome size of *D. odorifera* is estimated with the following formula: genome size = [Num (total k-mer) -Num (erroneous k-mer)]/mean depth of k-mer. 17-mers are counted as 55,992,417,644 from short clean reads. The total count of error k-mers is 449,157,644 and the k-mer depth is 85. Therefore, the genome size of *D. odorifera* is estimated to be around 653.45 Mb. Besides, we use around 25X Illumina's paired-end sequencing clean read to make GenomeScope analysis. The rate of genome heterozygosity estimated by GenomeScope (http://qb.cshl.edu/genomescope/) is around 0.59% and the genome size is around 623.46 Mb.

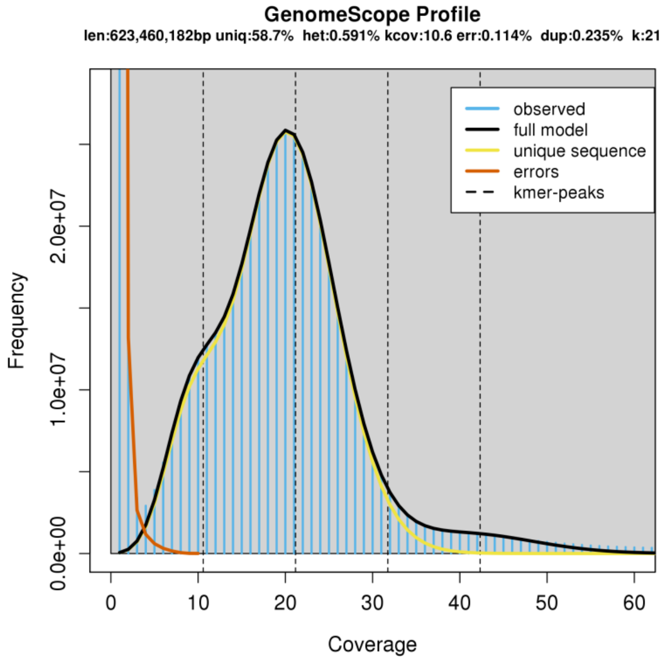

Our method: Genome size=653.45Mb, Heterozygosity=0.54%,TE=56.65%. The size of the assembled genome=638Mb, TE=54.17%. It's very close to our assessment.

Line 106-121 : The assembly has a good gene completeness and seems to be of high quality. But can you add some evaluations such as KAT histogramms which reflect the completeness of the assembly process. (https://kat.readthedocs.io/en/latest/).

Answers: Thanks for your comments. We further validate the assembly by comparing the cleaned read spectrum from two paired-end libraries with the copy number in the assembly using KAT toolkit v2.4.2.

The results show that the heterozygous rate is 0.81% and the assembly completeness i s 99.73%.

Line 278. Can you specify the kit used ? What is the quantity of material engaged in the preparations ?

Answers: Thanks for your reminding. The library detail information has been added.

Can you detail the preparation of the 10X sequencing library ? What is the quantity of DNA engaged in the library preparation. What is the size distribution of the DAN fragments used ?

Answers: Thanks for your reminding. The 10X library detail information has been added.

Line 293. Please add some informations about the HiC library preparation or add a reference. What is the enzyme used ?
Answers: The enzyme is DpnII and it used cuts at "GATC". The reference has been added.

Lines 298-299. You used the HiC data to organize the scaffolds into pseudo-molecules. Did you detect mis-assemblies after 10X scaffolding ?
Answers: We use the Hi-C data to organize the scaffolds into pseudo-molecules after 10X scaffolding, so we can't detect mis-assemblies by 10X Genomics link-reads. But according to the results of Hi-C, we could correct the mis-assemblies that may be introduced by 10x scaffolding. The Hi-C interaction heatmap indicate that the clusting result is good.

Line 327. Sequences produced from the RNA sequencing libraries are assembled. Can you detail the RNA extraction method ? Did you extract RNA from the fresh leaves too ?
Answers:We have added detail information about the RNA sequencing libraries, thanks for your comments.

Line 407. You perform analysis on RNA sequencing. But you spoke about replicates (line 193). Did you use the three replicates of each samples in your analysis ? Have you check the reproducibility of the results thanks to the replicates?
Answers: According to the editor' request, for Data Note in-depth analysis is not required. We have made some adjustments to the manuscript, thanks for your comments.

Typo :
Line 398 : repeated gene pairs
Line 198 : co-expressed instead of co-expression
Line 224 : has instead of was
Answers:Revised, thanks for your comments.

Reviewer #2: The manuscript describes genome assembly, annotation, Transcript sequence of D. odorifera and very preliminary analysis of genome and some of the medicinally important genes. The quality of genome assembly and annotation is very

high. However, the other analysis are routine. A big problem for me to assess this manuscript is I could not find the legends of the figures in the main text. The genome assembly statistics table must represent the step-by-step improvement for each sequencing technique. The approximate time of WGD is not mentioned. I did not find any comparative structure genomic analysis between D. odorifera and nearest species (MCScanX?). At the same time, what is the speciality in the sequence of medicinally important genes of D. odorifera vis-a-vis the nearest species that allowed it to produce novel compounds. To publish in Gigascience, I believe some useful biological analysis and information are required. A more informative and biologically oriented manuscript would be suitable for this journal.

Answers: Thanks for your comment. According to the editor' advice, this manuscript was transferred to Data Note format. We have made some adjustments to the manuscript according to your suggestions. Figure legends has been added to the text, and the statistics of assembly process has been added to the assembly statistics table. The approximate time of WGD was described.
The collinearity analyses between *Dalbergia odorifera* and *Arachis duranensis* have been done using jcvi method (https://github.com/tanghaibao/jcvi). Firstly, we find out all the published genome of legumes on the website: https://www.plabipd.de/plant_genomes_pa.ep. According to the divergence time from the website: http://www.timetree.org/, *Arachis duranensis* was the closest legume specie to *Dalbergia odorifera* at present. So we make a collinearity analysis of *Arachis duranensis* between *Dalbergia odorifera*. The result figure is as follow:

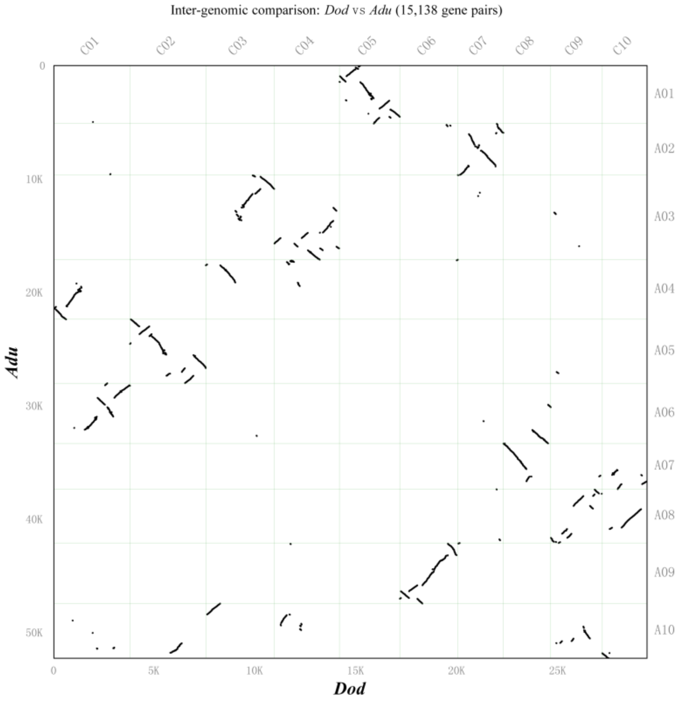

While due to the long genetic distance (~40 MYA) and low genome collinearity between *Arachis duranensis* and *Dalbergia odorifera*, it is not suitable for verification of *Dalbergia odorifera* assembly by comparative genomics so far.

According to the editor' request, for Data Note in-depth analysis is not required. Comparative genomics is usually recommended, but we do not necessarily need to do any biological experimentation to meet this requirement. Thanks again for your valuable comments.
