# Supplementary material for: The chromosome-level draft genome of Dalbergia odorifera
Source: Gigascience. 2020 Aug 18;9(8):giaa084. doi: 10.1093/gigascience/giaa084 (PMC7433187; doi:10.1093/gigascience/giaa084)
Supplement: giaa084_Supplemental_Files [file giaa084_supplemental_files.zip › Additional file 1.docx]

Additional file 1

**Table S1. Latin and abbreviated plant names used in various tables and figures.**

| **Latin Name** | **Abbreviation** | **Source** | **Version** |
| --- | --- | --- | --- |
| *Arachis duranensis* | Adu | NCBI | GCF_000817695.2 |
| *Arabidopsis thaliana* | Ath | Ensembl | release-32 |
| *Cajanus cajan* | CCa | NCBI | GCF_000340665.1 |
| *Dalbergia odorifera* | Dod | This project | this study |
| *Eucalyptus grandis* | Egr | NCBI | GCF_000612305.1 |
| *Glycine max* | Gma | Ensembl | release-32 |
| *Glycine soja* | Gso | NCBI | GCA_004193775.1 |
| *Medicago truncatula* | Mtr | Ensembl | release-32 |
| *Populus trichocarpa* | Ptr | Ensembl | release-32 |
| *Vitis vinifera* | Vvi | Ensembl | release-32 |

**Table S2. Pacbio data quality assessment.**

| Read_type | Read_base | Read_Number | Read_length(max) | Read_length(mean) | Read_length(N50) |
| --- | --- | --- | --- | --- | --- |
| Polymerase | 67,804,898,057 | 4,707,656 | 104,019 | 14,403 | 22,493 |
| Insertsize | 57,982,731,586 | 4,707,656 | 84,801 | 12,316 | 18,373 |
| Subreads | 67,743,628,461 | 6,047,570 | 84,801 | 11,201 | 17,402 |

**Table S3. Statistics of 17-mer analysis.**

| **K-mer** | **Depth** | **K-mer number** | **Revised Genome_size (Mb)** | **Heterozygous (%)** | **Repeat (%)** |
| --- | --- | --- | --- | --- | --- |
| 17 | 85 | 55,992,417,644 | 653.45 | 0.54 | 56.65 |

Note: All 17-mer sequences were extracted from paired-end clean reads that passed the quality control (QC) step from next-generation sequencing libraries (350 bp), and the frequency of each 17-mer was calculated and plotted.

**Table S4. Mapping results of Illumina paired-end reads with short insert sizes.**

| **Parameters** | **Percentage (%)** |
| --- | --- |
| Reads Mapping rate | 97.93 |
| Genome Coverage | 99.63 |
| Coverage at least 4X | 99.46 |
| Coverage at least 10X | 99.26 |
| Coverage at least 20X | 98.95 |

**Table S5. Genome completeness evaluated by CEGMA.**

| **Species** | **Complete** | | **Complete + partial** | |
| --- | --- | --- | --- | --- |
|  | Number | % completeness | Number | % completeness |
| *D. odorifera* | 233 | 93 | 235 | 94.76 |

**Table S6. Genome completeness evaluated by BUSCO.**

| **Species** | **BUSCO assessment results** |
| --- | --- |
| *D. odorifera* | C:92.2%[S:84.1%,D:8.1%],F:1.7%,M:6.1%,n:1440 |

Note: C (Complete BUSCOs) is the complete BUSCO; D (Complete and duplicated BUSCOs) is a complete BUSCO, but appears multiple times; F (Fragmented BUSCOs) is a fragmented BUSCO;

M (Missing BUSCOs) is the missing BUSCO; n is the number of genes used for evaluation.

**Table S7．Summary of Hi-C clustering.**

| **Group** | **Number** | **Length** |
| --- | --- | --- |
| Group0 | 20 | 79,613,017 |
| Group1 | 17 | 76,641,667 |
| Group2 | 10 | 66,664,306 |
| Group3 | 8 | 56,159,160 |
| Group4 | 6 | 56,046,058 |
| Group5 | 13 | 60,283,349 |
| Group6 | 14 | 54,483,937 |
| Group7 | 16 | 51,657,244 |
| Group8 | 14 | 46,752,440 |
| Group9 | 6 | 44,243,769 |
| TOTAL | 124 | 592,544,947 (92.84%) |

**Table S8. Summary of Hi-C assembly.**

| **Type** | **Input assembly** | **Hi-C assembly** |
| --- | --- | --- |
|  |  |  |
| Total Length | 638.26 Mb | 638.28 Mb |
| L50/N50 | 24 Scaffolds; 7.25 Mb | 5 Scaffolds; 56.16 Mb |
| L90/N90 | 91 Scaffolds; 1.53 Mb | 10 Scaffolds; 44.24 Mb |
| Longest Scaffolds | 26.65 Mb | 79.61 Mb |
| Number of Scaffolds | 480 | 384 |
| Contig N50 | 6.19 Mb | 5.92 Mb |

**Table S9. The genome assembly statistics by each technique.**

| Sequencing technique | Total size | Scaffold N50 size |
| --- | --- | --- |
| Pacbio | 637,662,611 | 6,178,971 |
| 10X | 638,264,760 | 7,249,437 |
| Hi-C | 638,276,160 | 56,159,160 |

**Table S10. Transposable elements (TEs) predicted by each method.**

| **Method** | **Repeat Size** | **% of genome** |
| --- | --- | --- |
| Tandem Repeats Finder | 23,276,422 | 3.65 |
| RepeatMasker | 332,705,455 | 52.13 |
| RepeatProteinmask | 71,048,270 | 11.13 |
| Total | 345,733,251 | 54.17 |

**Table S11. The sequencing statistics of RNAseq clean data.**

| Sample | Total reads | Total Length | %GC | %Q20 | %Q30 |
| --- | --- | --- | --- | --- | --- |
| flower | 54,518,696 | 8,177,804,400 | 44.84 | 97.49 | 93.03 |
| leaf | 65,003,696 | 9,750,554,400 | 45.75 | 97.72 | 93.88 |
| root | 55,234,402 | 8,285,160,300 | 46.4 | 97.51 | 93.53 |
| seed | 64,397,922 | 9,659,688,300 | 46.91 | 97.93 | 94.36 |
| stem | 59,867,950 | 8,980,192,500 | 45.63 | 97.66 | 93.81 |

**Table S12. The RNAseq assembly quality statistics.**

|  | Total Number | Total Length | Mean Length | N50 | GC(%) |
| --- | --- | --- | --- | --- | --- |
| Trinity.fasta | 225,859 | 250,797,804 | 1,110 | 1,966 | 40.53 |

**Table S13. Summary of gene predictions.**

| **Gene set** | **Number of genes** | **CDS+intron length (avg.)** | **CDS length (avg.)** | **Exon length (avg.)** | **Intron length (avg.)** | **Exons per gene (avg.)** |
| --- | --- | --- | --- | --- | --- | --- |
| Augustus | 37,360 | 2,854.22 | 983.20 | 232.15 | 578.32 | 4.24 |
| GlimerHMM | 64,297 | 8,243.39 | 612.20 | 196.58 | 3,609.32 | 3.11 |
| SNAP | 62,758 | 3,745.55 | 620.52 | 167.99 | 1,160.11 | 3.69 |
| Genscan | 35,685 | 10,409.38 | 1,068.19 | 195.01 | 2,086.14 | 5.48 |
| Geneid | 56,042 | 4,629.48 | 714.89 | 183.83 | 1,355.03 | 3.89 |
| *A. thaliana* | 34,850 | 2,507.15 | 960.37 | 268.47 | 600.19 | 3.58 |
| *P. trichocarpa* | 30,951 | 2,871.85 | 1,102.12 | 272.55 | 581.44 | 4.04 |
| *E. grandis* | 23,954 | 3,556.58 | 1,319.92 | 290.36 | 630.80 | 4.55 |
| *M. truncatula* | 44,252 | 2,416.16 | 922.02 | 265.33 | 603.69 | 3.48 |
| *A. duranensis* | 27,342 | 3,345.60 | 1,331.06 | 296.00 | 576.11 | 4.50 |
| *M. domestica* | 25,516 | 3,224.61 | 1,282.33 | 301.65 | 597.43 | 4.25 |
| *G. max* | 35,295 | 3,021.44 | 1,056.61 | 260.00 | 641.29 | 4.06 |
| Cufflinks | 56,774 | 6,453.37 | 2,212.77 | 353.89 | 807.32 | 6.25 |
| PASA | 83,515 | 3,449.56 | 967.74 | 205.06 | 667.30 | 4.72 |
| EVM | 39,967 | 3,176.73 | 956.99 | 223.02 | 674.47 | 4.29 |
| PASA-update | 39,731 | 3,154.86 | 964.99 | 225.12 | 666.31 | 4.29 |
| Final_set | 30,310 | 3,682.30 | 1,121.36 | 227.50 | 651.81 | 4.93 |

**Table S14. Number of genes with homologs or functional classifications based on different databases.**

| **Database** |  | **Annotated Number** | **Annotated Percentage (%)** |
| --- | --- | --- | --- |
| NR |  | 28,010 | 92.4 |
| Swiss-Prot |  | 22,612 | 74.6 |
| KEGG |  | 21,186 | 69.9 |
|  | All | 23,867 | 78.7 |
| InterPro | Pfam | 22,264 | 73.5 |
|  | GO | 16,530 | 54.5 |
| Annotated |  | 28,069 | 92.6 |
| Total |  | 30,310 | - |

**Table S15. Number of all types of noncoding RNAs.**

| **Type** | | **Copy (w*)** | **Average length (bp)** | **Total length (bp)** | **% of genome** |
| --- | --- | --- | --- | --- | --- |
| miRNA | | 747 | 112.4618 | 84,009 | 0.013162 |
| tRNA | | 582 | 74.9983 | 43,649 | 0.006839 |
| rRNA | rRNA | 112 | 241.8482 | 27,087 | 0.004244 |
|  | 18S | 10 | 1445.1 | 14,451 | 0.002264 |
|  | 28S | 37 | 136.0541 | 5,034 | 0.000789 |
|  | 5.8S | 8 | 173.375 | 1,387 | 0.000217 |
|  | 5S | 57 | 109.0351 | 6,215 | 0.000974 |
| snRNA | snRNA | 473 | 111.9662 | 52,960 | 0.008297 |
|  | CD-box | 312 | 100.7885 | 31,446 | 0.004927 |
|  | HACA-box | 41 | 132.9756 | 5,452 | 0.000854 |
|  | splicing | 119 | 133.8992 | 15,934 | 0.002496 |

Note: w*, whole-genome annotation.
