# Supplementary material for: The chromosome-level draft genome of Dalbergia odorifera
Source: Gigascience. 2020 Aug 18;9(8):giaa084. doi: 10.1093/gigascience/giaa084 (PMC7433187; doi:10.1093/gigascience/giaa084)

Additional file 2

**Fig. S1. Cross section of *D. odorifera* wood.**


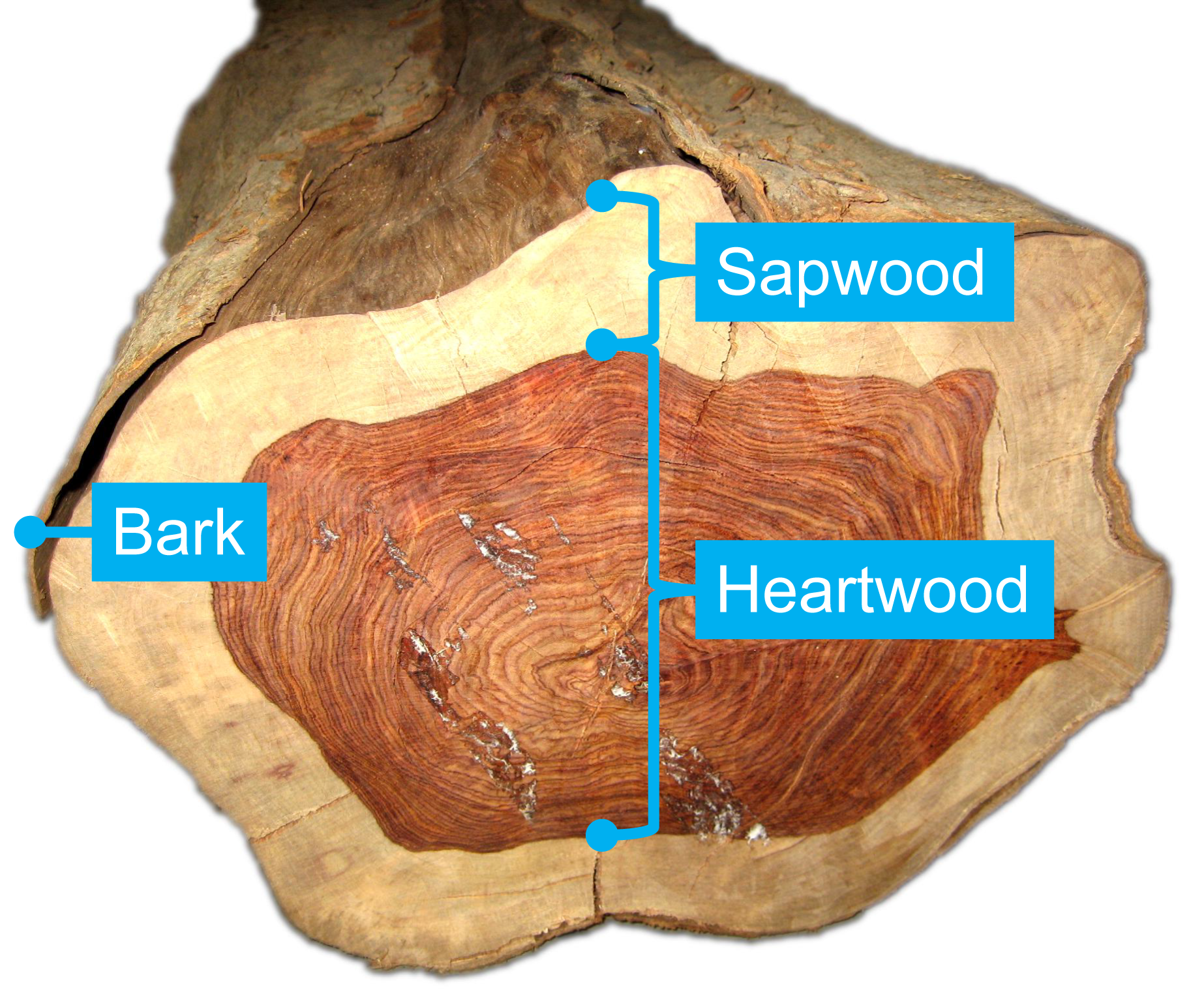


**Fig. S2. Frequency distribution of 17-mers in the *D. odorifera* genome.**X-axis represents the sequencing depth. Y-axis represents the frequency of K-mers at a given sequencing depth. The estimated genome size for *D. odorifera* is 653.45 Mb.


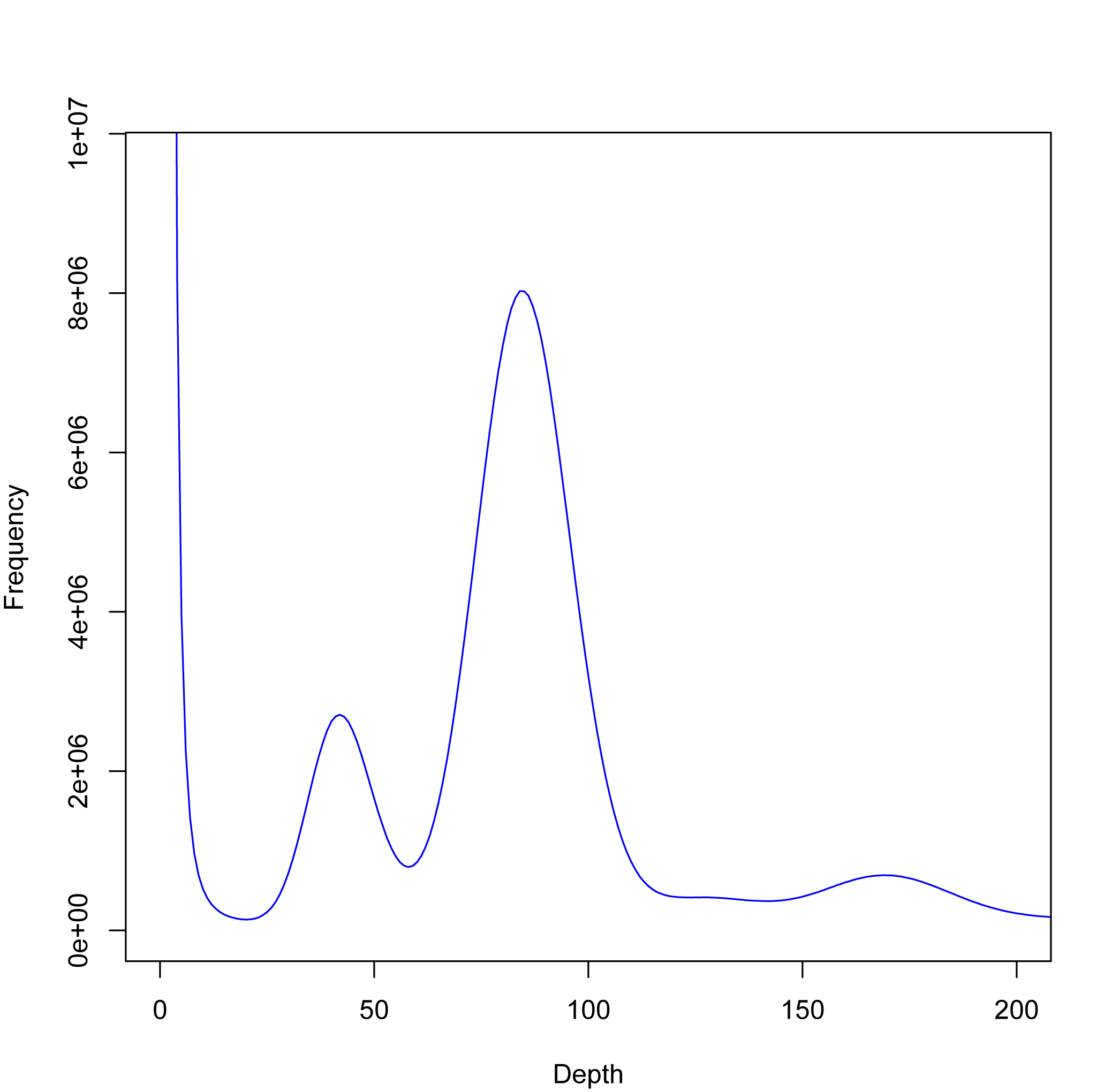


**Fig. S3. The K-mer distribution of ~25X Illumina paired-end reads using GenomeScope based on k value of 21.** K-mer occurrences (x axis) were plotted against their frequencies (y axis).

**
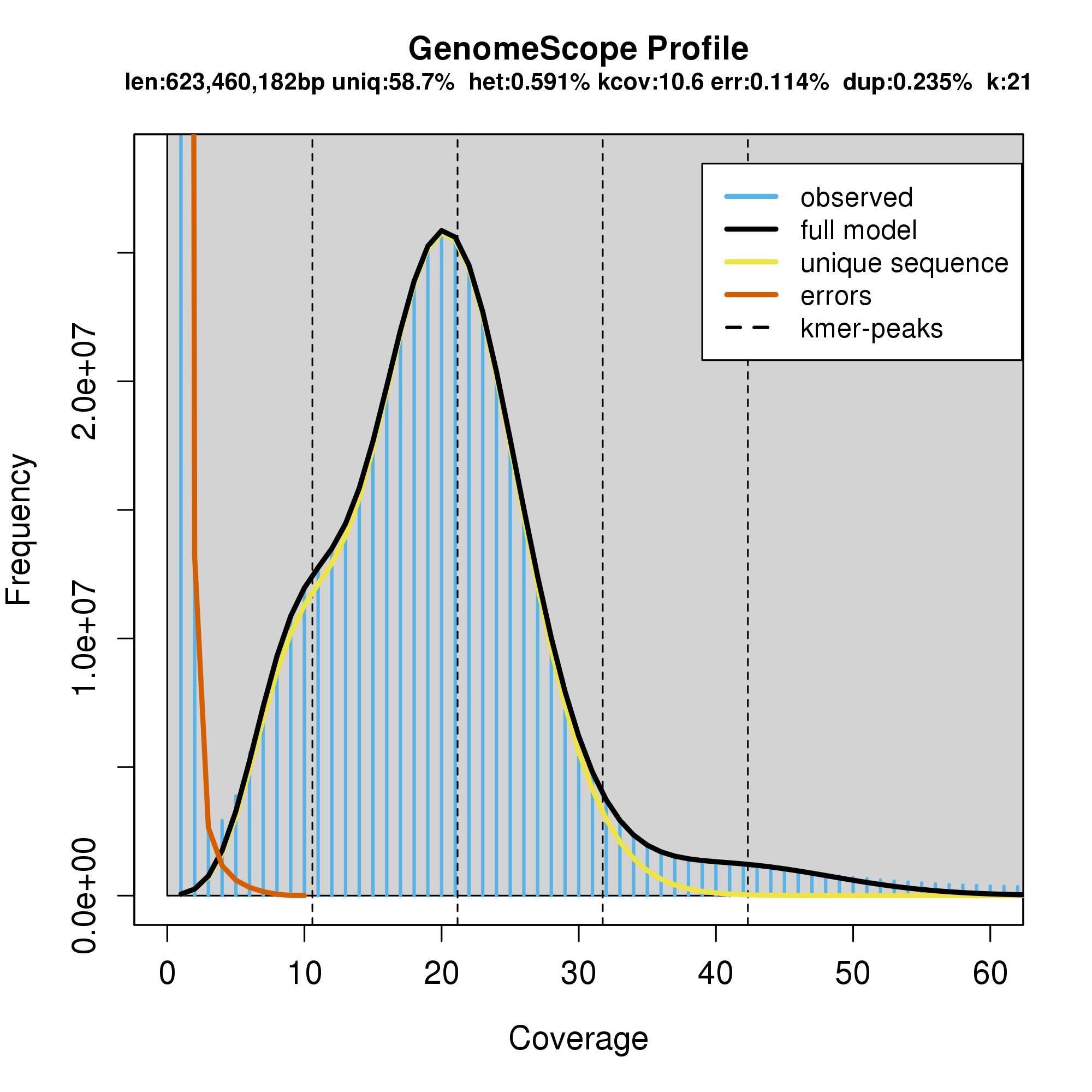
**

**Fig. S4. Sequencing depth based on the mapping of paired-end reads with short insert sizes via HiSeq.**


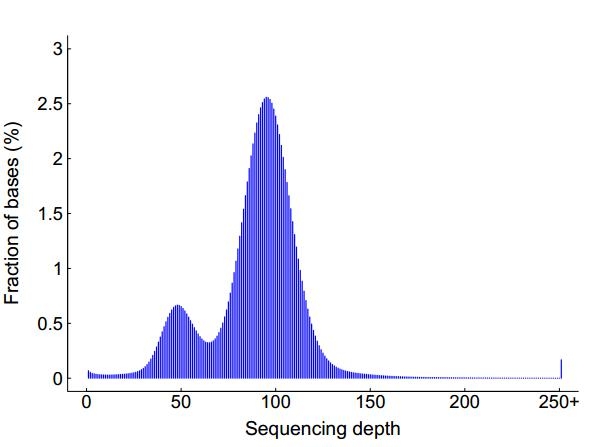


**Fig. S5. GC depth plot and sequencing depth of the Illumina sequencing.** The GC content is concentrated around 34%, and there is no obvious separation of the scatter plots, indicating that there is no external contamination of the genome.


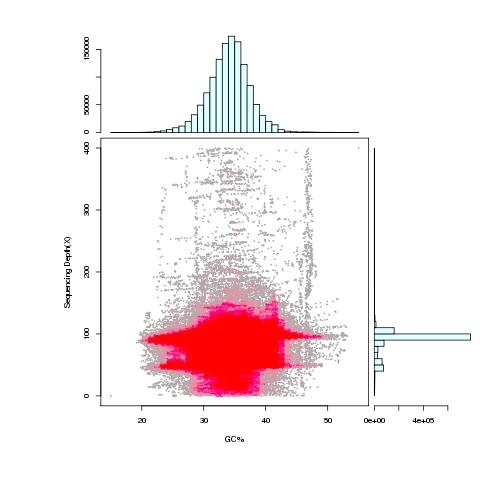


**Fig. S6. Genome-wide Hi-C heat map.** There are 10 pseudomolecules.


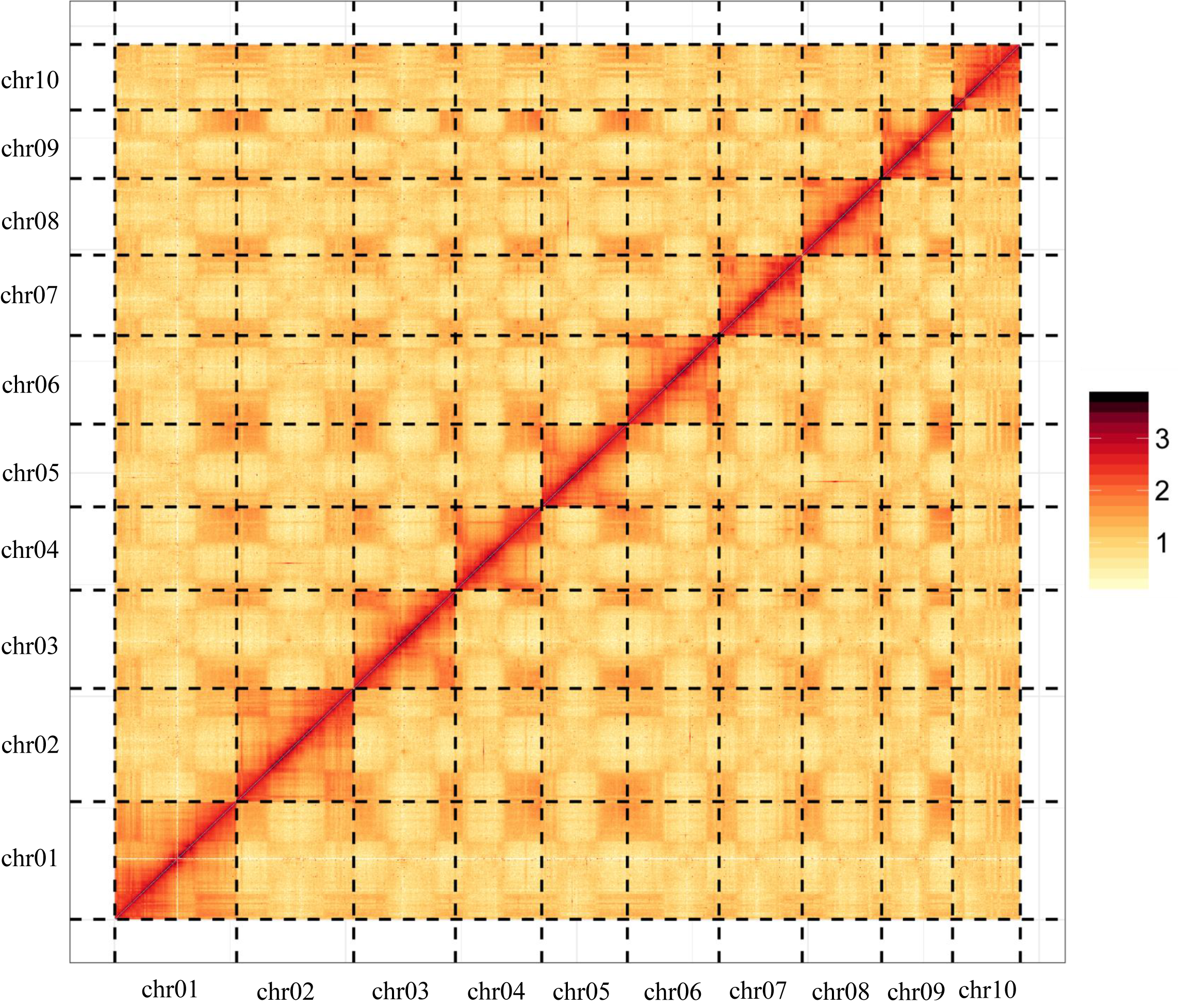


**Fig. S7. Scaffold N50 statistics of 13 plant genomes that belong to Fabaceae family and other related species.**

**Fig. S8. Distribution of the divergence rate of each type of TE in *D.* *odorifera*.** The divergence rate was calculated between the identified TE in the genome using a homology-based method and the consensus sequence in Repbase.


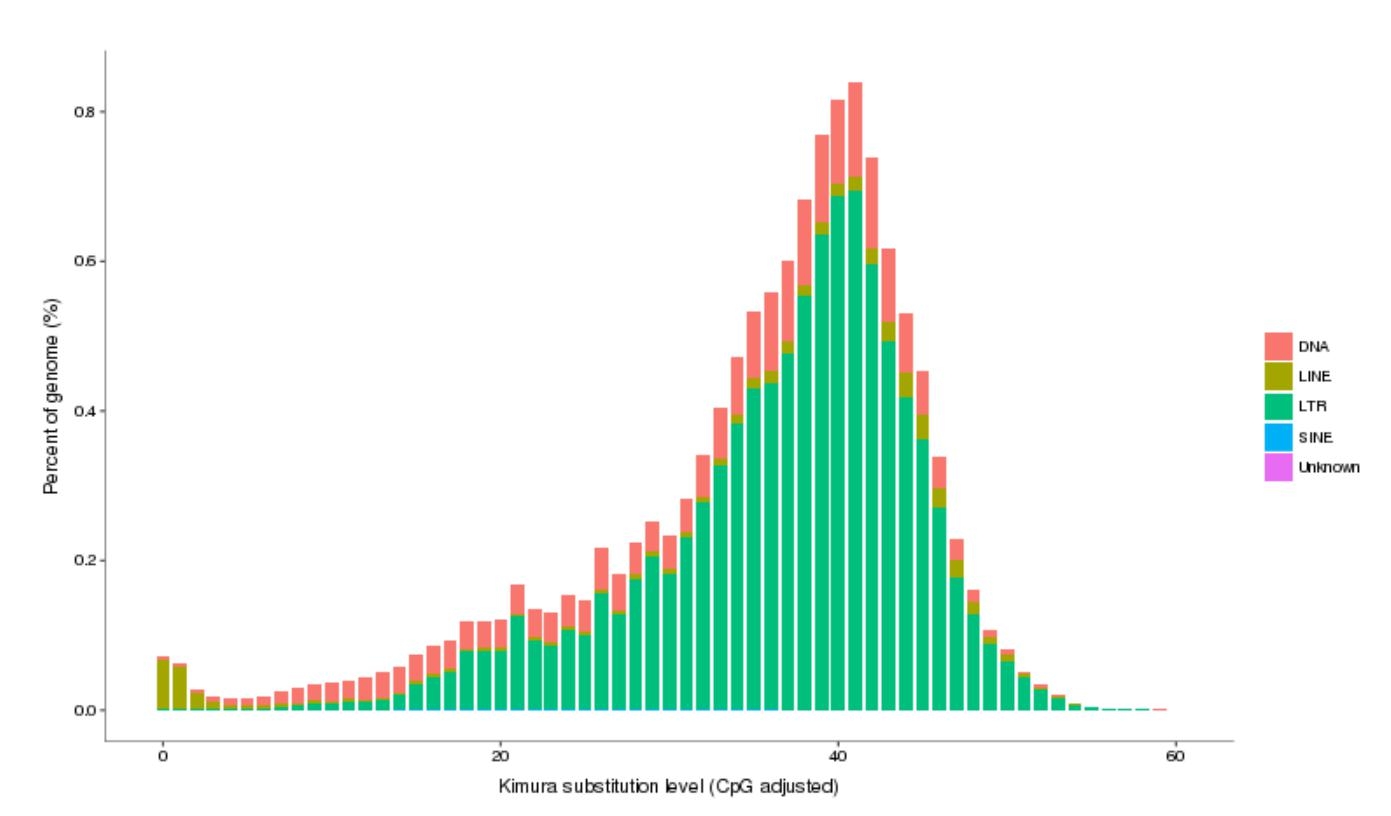


**Fig. S9. The evidences supporting gene prediction.**


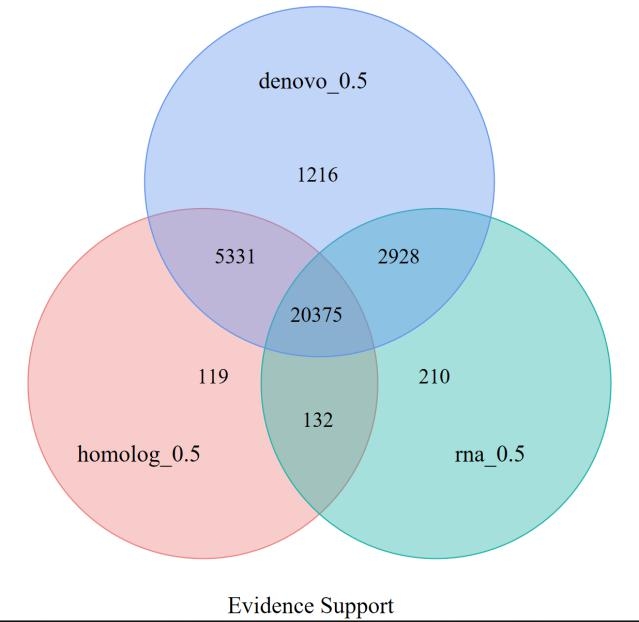


Note: *denovo* means *ab initio* prediction, homolog means homologous comparison, and rna means RNA-Seq-based annotation. 0.5 means at least 50% overlap.

**Fig. S10. Comparison of the distribution of five features in the final gene set for the eight plants.** Window refers to the length of each point. No obvious unexpected differences were detected among organisms, confirming the high quality of the gene structure annotation.


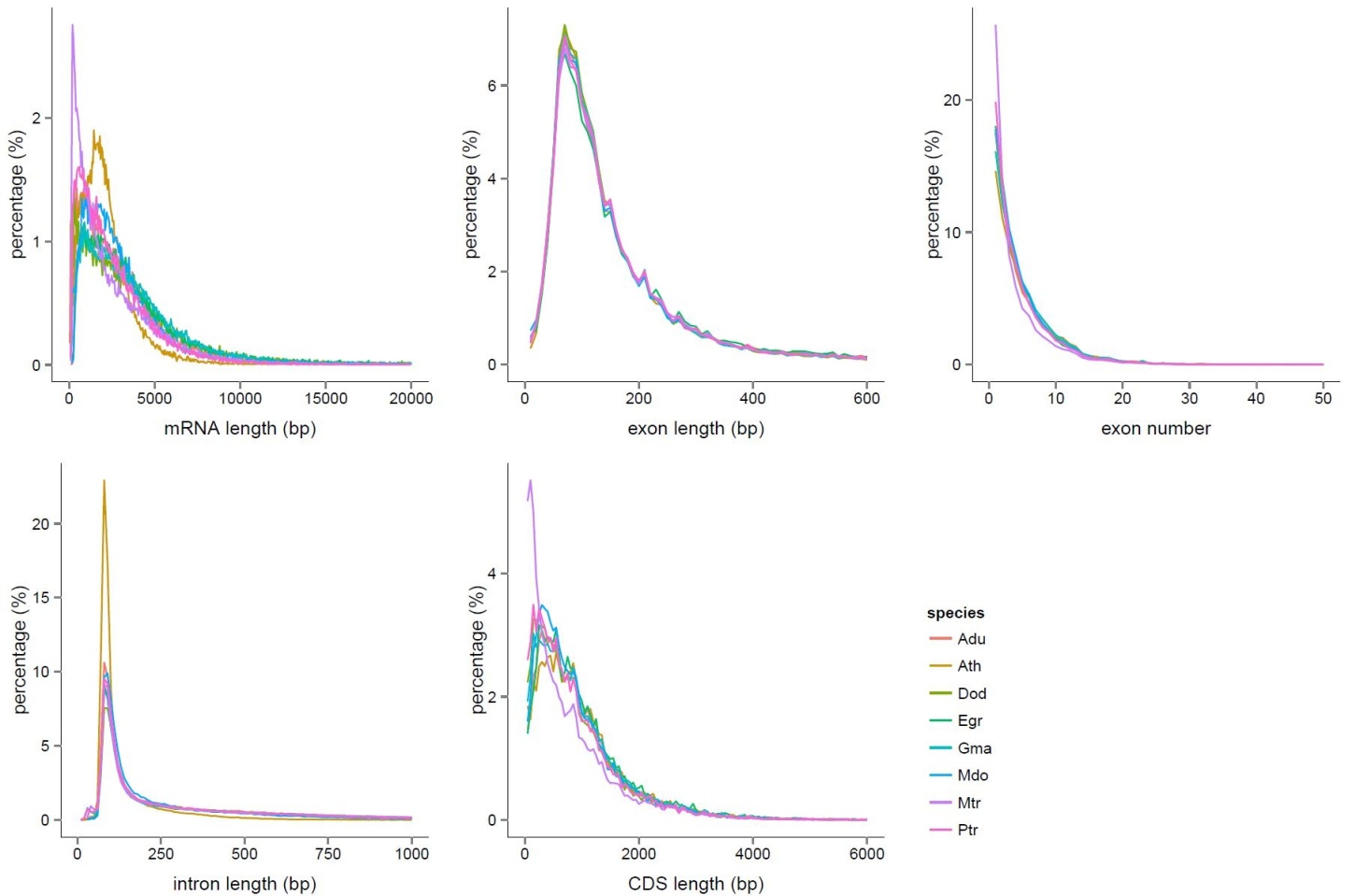


**Fig. S11. Statistics of gene families in the 10 species examined.**


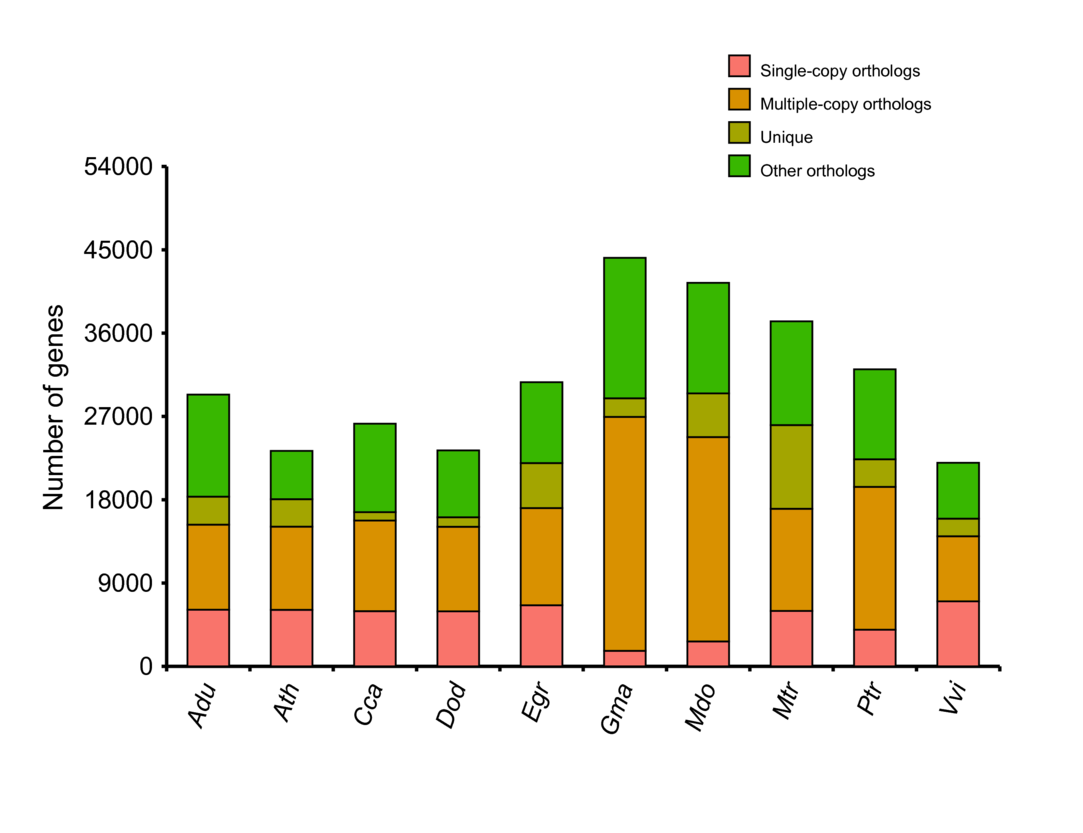


**Fig. S12. Pathway enrichment analysis of 577 *D. odorifera* specific gene families.**


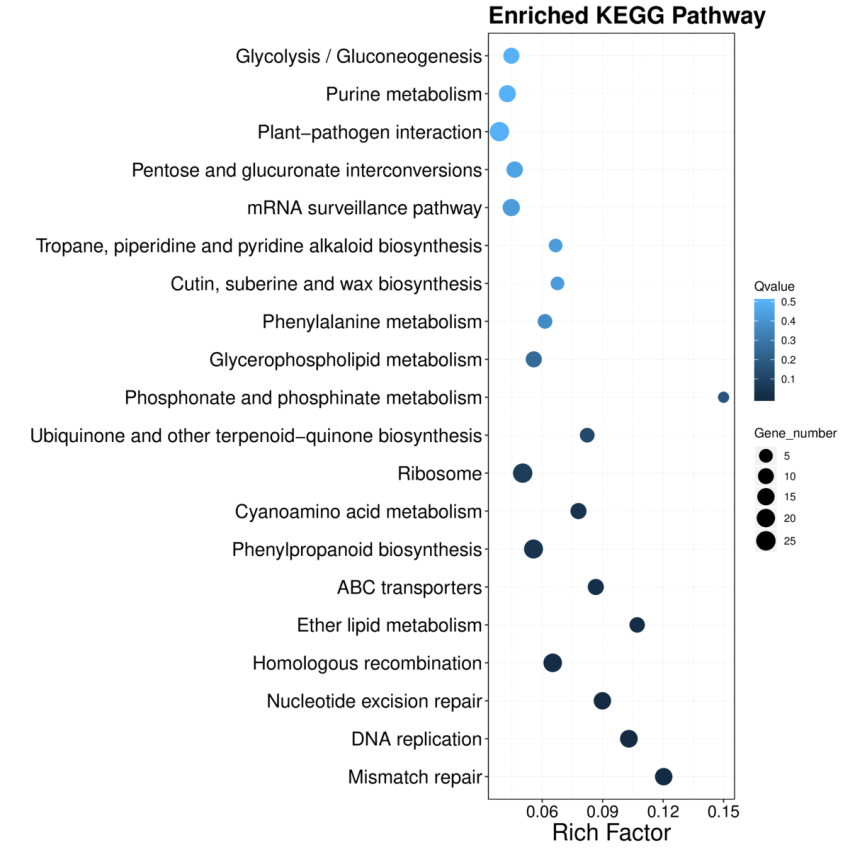


Note: Rich factor refers to the ratio of the number of genes enriched in the pathway to the number of annotated genes. The larger the Rich factor, the greater the degree of enrichment.

**Fig. S13. Pathway enrichment analysis of expanded gene families in *D. odorifera*.**


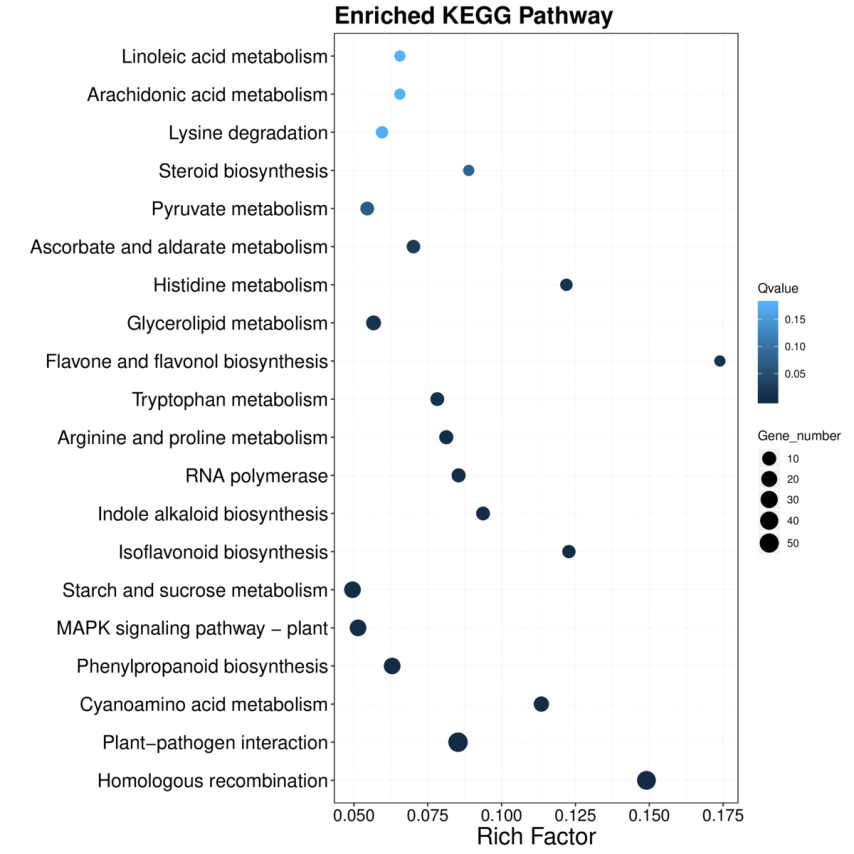


Note: Rich factor refers to the ratio of the number of genes enriched in the pathway to the number of annotated genes. The larger the Rich factor, the greater the degree of enrichment.

**Fig. S14. Estimation of divergence times.** The red circles at the nodes show the estimated divergence times using Ath-Ptr (97-109 million years ago [Mya]), Cca-Gma (17.8-27.0 Mya), Gma-Mtr (46-60 Mya), Gma-Mdo (82-116 Mya), and Ath-Vvi (105-115 Mya) as the calibration times. All calibration times were obtained from timetree (http://www.timetree.org/).


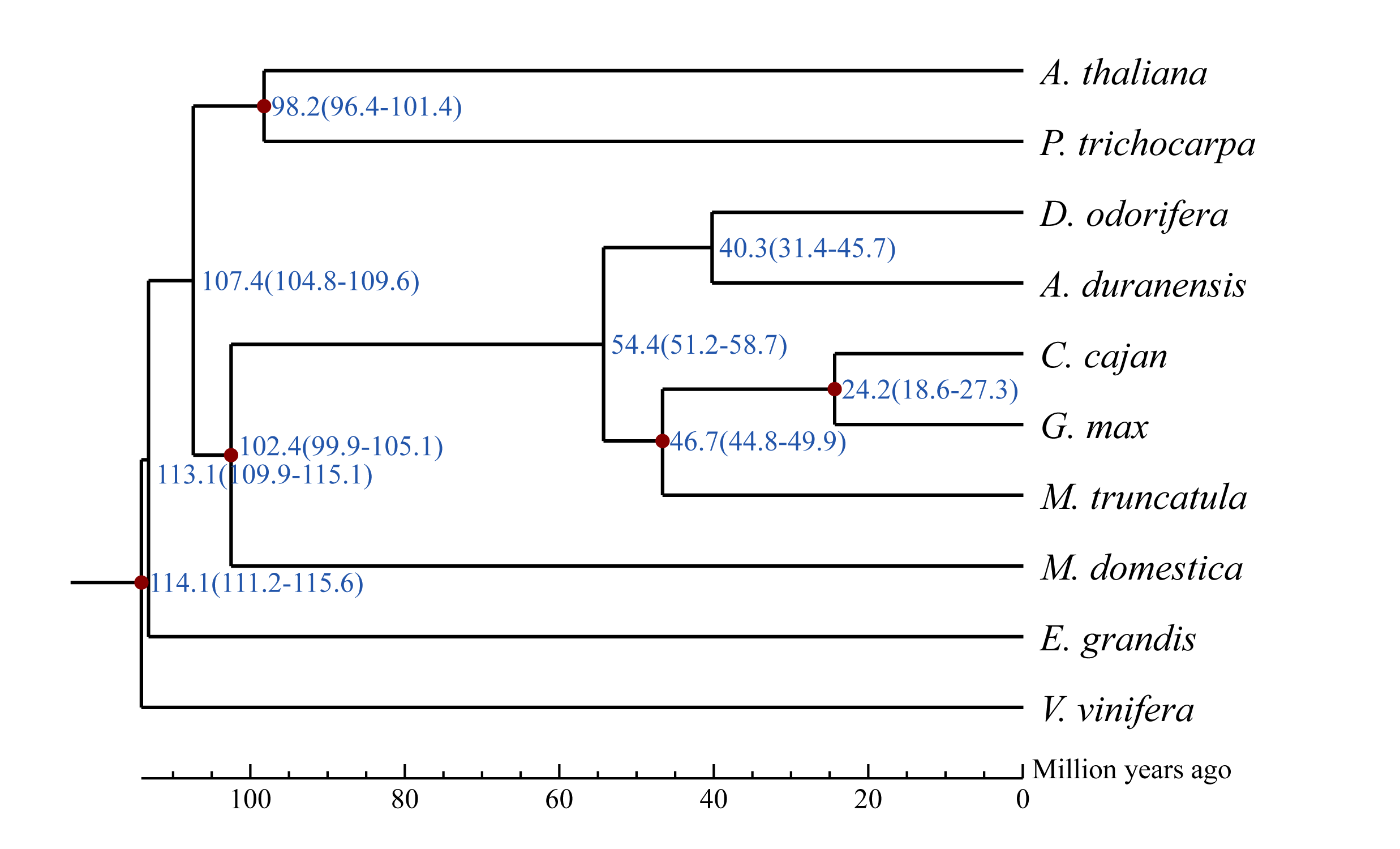

Supplement: giaa084_Supplemental_Files [file giaa084_supplemental_files.zip › Additional file 2.docx]
