# Supplementary material for: The chromosome-level draft genome of Dalbergia odorifera
Source: Gigascience. 2020 Aug 18;9(8):giaa084. doi: 10.1093/gigascience/giaa084 (PMC7433187; doi:10.1093/gigascience/giaa084)
Supplement: giaa084_Response_to_Reviewer_Comments_Original_Submission [file giaa084_response_to_reviewer_comments_original_submission.pdf]

Author's Response To Reviewer Comments

Close

For a Data Note in-depth analysis is not required, but we do require some validation. Comparative genomics is a good way to do this and is usually recommended, but you do not necessarily need to do any biological experimentation to meet this requirement.
Answers: Thanks for your reminding. The collinearity analyses have been done using jcvi method (https://github.com/tanghaibao/jcvi). Firstly, we find out all the published genome of legumes on the website: https://www.plabipd.de/plant_genomes_pa.ep. According to the divergence time from the website: http://www.timetree.org/, Arachis duranensis was the closest legume specie to Dalbergia odorifera. So we make a collinearity analysis of Arachis duranensis between Dalbergia odorifera. While due to the long genetic distance (~40 MYA) and low genome collinearity between Arachis duranensis and Dalbergia odorifera, it is not suitable for verification of Dalbergia odorifera assembly by comparative genomics so far.Since I can't upload the pictures in the column of response to reviewers, I have sent you the collinearity diagram and the document responding to the reviewer's comments by e-mail.

In addition, please register any new software application in the bio.tools and SciCrunch.org databases to receive RRID (Research Resource Identification Initiative ID) and biotoolsID identifiers, and include these in your manuscript. This will facilitate tracking, reproducibility and re-use of your tool.
Answers: Thanks for your reminding. The RRID have been added.

Line 79. The DNA extraction was performed using the CTAB method. There is no reference for this method. Please add a reference or explain the method.
Answers: Thanks for your reminding. The reference has been added.

Line 97. The genome size was estimated by k-mer distribution analysis. Please clarify which method you used ? How did you estimate the heterozygosity rate ? Did you compare your results with results from Genomescope for example ?
Answers: The genome size of D. odorifera is estimated with the following formula: genome size = [Num (total k-mer) -Num (erroneous k-mer)]/mean depth of k-mer. 17-mers are counted as 55,992,417,644 from short clean reads. The total count of error k-mers is 449,157,644 and the k-mer depth is 85. Therefore, the genome size of D. odorifera is estimated to be around 653.45 Mb. Besides, we use around 25X Illumina's paired-end sequencing clean read to make GenomeScope analysis. The rate of genome heterozygosity estimated by GenomeScope (http://qb.cshl.edu/genomescope/) is around 0.59% and the genome size is around 623.46 Mb.
Our method: Genome size=653.45Mb, Heterozygosity=0.54%,TE=56.65%. The size of the assembled genome=638Mb, TE=54.17%. It's very close to our assessment.

Line 106-121 : The assembly has a good gene completeness and seems to be of high quality. But can you add some evaluations such as KAT histogramms which reflect the completeness of the assembly process. (https://kat.readthedocs.io/en/latest/).
Answers: Thanks for your comments. We further validate the assembly by comparing the cleaned read spectrum from two paired-end libraries with the copy number in the assembly using KAT toolkit v2.4.2. The results show that the heterozygous rate is 0.81% and the assembly completeness is 99.73%.

Line 278. Can you specify the kit used ? What is the quantity of material engaged in the preparations ?
Answers: Thanks for your reminding. The library detail information has been added.

Can you detail the preparation of the 10X sequencing library ? What is the quantity of DNA engaged in the library preparation. What is the size distribution of the DAN fragments used ?
Answers: Thanks for your reminding. The 10X library detail information has been added.

Line 293. Please add some informations about the HiC library preparation or add a reference. What is the enzyme used ?
Answers: The enzyme is DpnII and it used cuts at "GATC". The reference has been added.

Lines 298-299. You used the HiC data to organize the scaffolds into pseudo-molecules. Did you detect mis-assemblies after 10X scaffolding ?
Answers: We use the Hi-C data to organize the scaffolds into pseudo-molecules after 10X scaffolding, so we can't detect mis-assemblies by 10X Genomics link-reads. But according to the results of Hi-C, we could correct the mis-assemblies that may be introduced by 10x scaffolding. The Hi-C interaction heatmap indicate that the clusting result is good.

Line 327. Sequences produced from the RNA sequencing libraries are assembled. Can you detail the RNA extraction method ? Did you extract RNA from the fresh leaves too ?
Answers:We have added detail information about the RNA sequencing libraries, thanks for your comments.

Line 407. You perform analysis on RNA sequencing. But you spoke about replicates (line 193). Did you use the three replicates of each samples in your analysis ? Have you check the reproducibility of the results thanks to the replicates?
Answers: According to the editor' request, for Data Note in-depth analysis is not required. We have made some adjustments to the manuscript, thanks for your comments.

Typo :
Line 398 : repeated gene pairs
Line 198 : co-expressed instead of co-expression
Line 224 : has instead of was
Answers:Revised, thanks for your comments.

Reviewer #2: The manuscript describes genome assembly, annotation, Transcript sequence of D. odorifera and very preliminary analysis of genome and some of the medicinally important genes. The quality of genome assembly and annotation is very high. However, the other analysis are routine. A big problem for me to assess this manuscript is I could not find the legends of the figures in the main text. The genome assembly statistics table must represent the step-by-step improvement for each sequencing technique. The approximate time of WGD is not mentioned. I did not find any comparative structure genomic analysis between D. odorifera and nearest species (MCScanX?). At the same time, what is the speciality in the sequence of medicinally important genes of D. odorifera vis-a-vis the nearest species that allowed it to produce novel compounds. To publish in Gigascience, I believe some useful biological analysis and information are required. A more informative and biologically oriented manuscript would be suitable for this journal.

Answers: Thanks for your comment. According to the editor' advice, this manuscript was transferred to Data Note format. We have made some adjustments to the manuscript according to your suggestions. Figure legends has been added to the text, and the statistics of assembly process has been added to the assembly statistics table. The approximate time of WGD was described.
The collinearity analyses between Dalbergia odorifera and Arachis duranensis have been done using jcvi method (https://github.com/tanghaibao/jcvi). Firstly, we find out all the published genome of legumes on the website: https://www.plabipd.de/plant_genomes_pa.ep. According to the divergence time from the website: http://www.timetree.org/, Arachis duranensis was the closest legume specie to Dalbergia odorifera at present. So we make a collinearity analysis of Arachis duranensis between Dalbergia odorifera. I can't upload pictures in the column of response to reviewers, so I sent the collinearity figure to the eidtor by e-mail.
While due to the long genetic distance (~40 MYA) and low genome collinearity between Arachis duranensis and Dalbergia odorifera, it is not suitable for verification of Dalbergia odorifera assembly by comparative genomics so far.
According to the editor' request, for Data Note in-depth analysis is not required. Comparative genomics is usually recommended, but we do not necessarily need to do any biological experimentation to meet this requirement. Thanks again for your valuable comments.

Close
