# Supplementary material for: The chromosome-level draft genome of Dalbergia odorifera
Source: Gigascience. 2020 Aug 18;9(8):giaa084. doi: 10.1093/gigascience/giaa084 (PMC7433187; doi:10.1093/gigascience/giaa084)
Supplement: giaa084_Reviewer_1_Report_Original_Submission — Caroline Belser -- 4/13/2020 Reviewed [file giaa084_reviewer_1_report_original_submission.pdf]

Reviewer Report

Title: The chromosome-level draft genome of Dalbergia odorifera

Version: Original Submission **Date:** 4/13/2020

Reviewer name: Caroline Belser

Reviewer Comments to Author:

This article is presenting the genome assembly of Dalbergia odorifera which is a tree of interest for its commercial and medicinal properties. The genome assembly is at the chromosome scale and has been well annotated. The phylogenetic and metabolite analysis produced very interesting results. But some methods had to be better described as listed below :

Line 79. The DNA extraction was performed using the CTAB method. There is no reference for this method. Please add a reference or explain the method.

Line 97. The genome size was estimated by k-mer distribution analysis. Please clarify which method you used ? How did you estimate the heterozygosity rate ? Did you compare your results with results from Genomescope for example ?

Line 106-121 : The assembly has a good gene completeness and seems to be of high quality. But can you add some evaluations such as KAT histogramms which reflect the completeness of the assembly process. (https://kat.readthedocs.io/en/latest/).

Line 278. Can you specify the kit used ? What is the quantity of material engaged in the preparations ? Can you detail the preparation of the 10X sequencing library ? What is the quantity of DNA engaged in the library preparation. What is the size distribution of the DAN fragments used ?

Line 293. Please add some informations about the HiC library preparation or add a reference. What is the enzyme used ?

Lines 298-299. You used the HiC data to organize the scaffolds into pseudo-molecules. Did you detect mis-assemblies after 10X scaffolding ?

Line 327. Sequences produced from the RNA sequencing libraries are assembled. Can you detail the RNA extraction method ? Did you extract RNA from the fresh leaves too ?

Line 407. You perform analysis on RNA sequencing. But you spoke about replicates (line 193). Did you use the three replicates of each samples in your analysis ? Have you check the reproducibility of the results thanks to the replicates?

Typo :

Line 398 : repeated gene pairs

Line 198 : co-expressed instead of co-expression

Line 224 : has instead of was

Level of Interest

Please indicate how interesting you found the manuscript: Choose an item.

Quality of Written English

Please indicate the quality of language in the manuscript: Choose an item.

Declaration of Competing Interests

Please complete a declaration of competing interests, considering the following questions:

- Have you in the past five years received reimbursements, fees, funding, or salary from an organisation that may in any way gain or lose financially from the publication of this manuscript, either now or in the future?
- Do you hold any stocks or shares in an organisation that may in any way gain or lose financially from the publication of this manuscript, either now or in the future?
- Do you hold or are you currently applying for any patents relating to the content of the manuscript?
- Have you received reimbursements, fees, funding, or salary from an organization that holds or has applied for patents relating to the content of the manuscript?
- Do you have any other financial competing interests?
- Do you have any non-financial competing interests in relation to this paper?

If you can answer no to all of the above, write 'I declare that I have no competing interests' below. If your reply is yes to any, please give details below.

I declare that I have no competing interests

I agree to the open peer review policy of the journal. I understand that my name will be included on my report to the authors and, if the manuscript is accepted for publication, my named report including any attachments I upload will be posted on the website along with the authors' responses. I agree for my report to be made available under an Open Access Creative Commons CC-BY license (http://creativecommons.org/licenses/by/4.0/). I understand that any comments which I do not wish to be included in my named report can be included as confidential comments to the editors, which will not be published.

Choose an item.

To further support our reviewers, we have joined with Publons, where you can gain additional credit to further highlight your hard work (see: https://publons.com/journal/530/gigascience). On publication of this paper, your review will be automatically added to Publons, you can then choose whether or not to claim your Publons credit. I understand this statement.

Yes Choose an item.
