# Supplementary material for: The chromosome-level draft genome of Dalbergia odorifera
Source: Gigascience. 2020 Aug 18;9(8):giaa084. doi: 10.1093/gigascience/giaa084 (PMC7433187; doi:10.1093/gigascience/giaa084)
Supplement: giaa084_Reviewer_2_Report_Original_Submission — Debasis Chattopadhyay -- 4/17/2020 Reviewed [file giaa084_reviewer_2_report_original_submission.pdf]

**Reviewer Report**

**Title: The chromosome-level draft genome of Dalbergia odorifera**

**Version: Original Submission** **Date:** 4/17/2020

**Reviewer name: Debasis Chattopadhyay**

**Reviewer Comments to Author:**

The manuscript describes genome assembly, annotation, Transcript sequence of D. odorifera and very preliminary analysis of genome and some of the medicinally important genes. The quality of genome assembly and annotation is very high. However, the other analysis are routine. A big problem for me to assess this manuscript is I could not find the legends of the figures in the main text. The genome assembly statistics table must represent the step-by-step improvement for each sequencing technique. The approximate time of WGD is not mentioned. I did not find any comparative structure genomic analysis between D. odorifera and nearest species (MCScanX?). At the same time, what is the speciality in the sequence of medicinally important genes of D. odorifera vis-a-vis the nearest species that allowed it to produce novel compounds. To publish in Gigascience, I believe some useful biological analysis and information are required. A more informative and biologically oriented manuscript would be suitable for this journal.

**Level of Interest**

Please indicate how interesting you found the manuscript: Choose an item.

**Quality of Written English**

Please indicate the quality of language in the manuscript: Choose an item.

**Declaration of Competing Interests**

Please complete a declaration of competing interests, considering the following questions:

- Have you in the past five years received reimbursements, fees, funding, or salary from an organisation that may in any way gain or lose financially from the publication of this manuscript, either now or in the future?
- Do you hold any stocks or shares in an organisation that may in any way gain or lose financially from the publication of this manuscript, either now or in the future?
- Do you hold or are you currently applying for any patents relating to the content of the manuscript?
- Have you received reimbursements, fees, funding, or salary from an organization that holds or has applied for patents relating to the content of the manuscript?
- Do you have any other financial competing interests?

- Do you have any non-financial competing interests in relation to this paper?

If you can answer no to all of the above, write 'I declare that I have no competing interests' below. If your reply is yes to any, please give details below.

I declare that I have no competing interest.

I agree to the open peer review policy of the journal. I understand that my name will be included on my report to the authors and, if the manuscript is accepted for publication, my named report including any attachments I upload will be posted on the website along with the authors' responses. I agree for my report to be made available under an Open Access Creative Commons CC-BY license (http://creativecommons.org/licenses/by/4.0/). I understand that any comments which I do not wish to be included in my named report can be included as confidential comments to the editors, which will not be published.

Choose an item.

To further support our reviewers, we have joined with Publons, where you can gain additional credit to further highlight your hard work (see: https://publons.com/journal/530/gigascience). On publication of this paper, your review will be automatically added to Publons, you can then choose whether or not to claim your Publons credit. I understand this statement.

Yes Choose an item.
